# Asymmetric Mannich reactions of (*S*)-*N*-*tert*-butylsulfinyl-3,3,3-trifluoroacetaldimines with yne nucleophiles

**DOI:** 10.3762/bjoc.16.217

**Published:** 2020-10-29

**Authors:** Ziyi Li, Li Wang, Yunqi Huang, Haibo Mei, Hiroyuki Konno, Hiroki Moriwaki, Vadim A Soloshonok, Jianlin Han

**Affiliations:** 1 Jiangsu Co-Innovation Center of Efficient Processing and Utilization of Forest Resources, College of Chemical Engineering, Nanjing Forestry University, Nanjing 210037, Jiangsu, China; 2 Department of Biological Engineering, Graduate School of Science and Engineering, Yamagata University, Yonezawa, Yamagata 992-8510, Japan; 3 Hamari Chemical Ltd., 1-4-29 Kunijima, Higashi-Yodogawa-ku, Osaka 533-0024, Japan; 4 Department of Organic Chemistry I, Faculty of Chemistry, University of the Basque Country UPV/EHU, Paseo Manuel Lardizábal 3, 20018 San Sebastián, Spain; 5 IKERBASQUE, Basque Foundation for Science, Alameda Urquijo 36-5, Plaza Bizkaia, 48011 Bilbao, Spain

**Keywords:** arylethynes, asymmetric Mannich reaction, C-nucleophile, CF_3_-aldimine, fluorinated propargylamine

## Abstract

In the present work, arylethynes were studied as new C-nucleophiles in the asymmetric Mannich addition reactions with (*S*)-*N*-*tert*-butylsulfinyl-3,3,3-trifluoroacetaldimine. The reactions were conducted under operationally convenient conditions affording the corresponding Mannich adducts with up to 87% yield and 70:30 diastereoselectivity. The isomeric products can be separated using regular column chromatography to afford diastereomerically pure compounds. The purified Mannich addition products were deprotected to give the target enantiomerically pure trifluoromethylpropargylamines. A mechanistic rationale for the observed stereochemical outcome is discussed.

## Introduction

**Figure 1:**
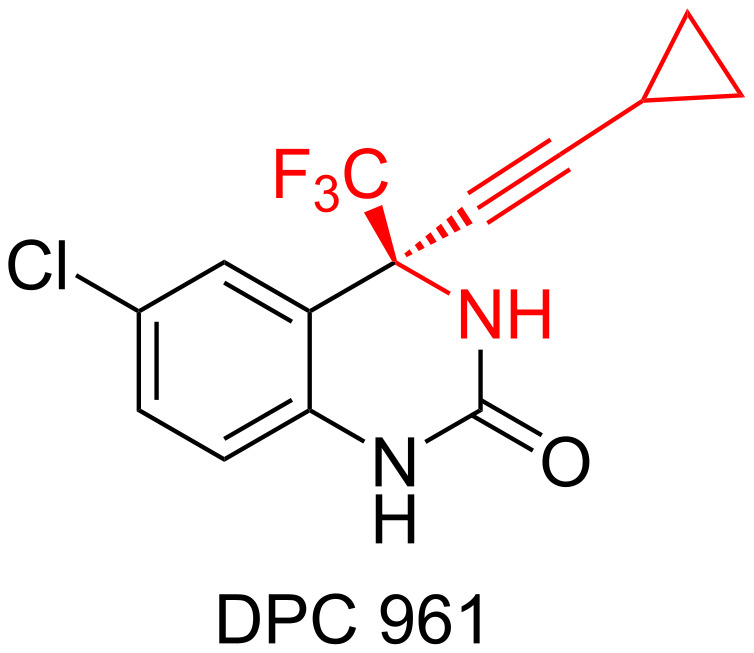
Anti-HIV compound containing a trifluoromethylpropargylamine moiety.

In recent years, substitution of hydrogen by fluorine atoms or fluorine-containing groups usually provides unexpected biological and physicochemical properties, which thus has become an established approach for the development of pharmaceuticals [1–9], agrochemicals [10–14], and advanced materials [15–19]. On the other hand, chiral propargylamine represents a very important type of organic intermediates, which has been successfully used in the synthesis of natural products and biologically relevant heterocyclic compounds [20–24]. Thus, fluorinated propargylamine, in particular, chiral trifluoromethylpropargylamine, should be considered of great research interest due to the apparently advantageous pharmaceutical profile of CF_3_-containing drugs [25–26]. For example, DPC 961 contains the trifluoromethylpropargylamine moiety and has been developed as the inhibitor against non-nucleoside reverse transcriptase for the treatment of human immunodeficiency virus [27] (Figure 1).

**Scheme 1:**
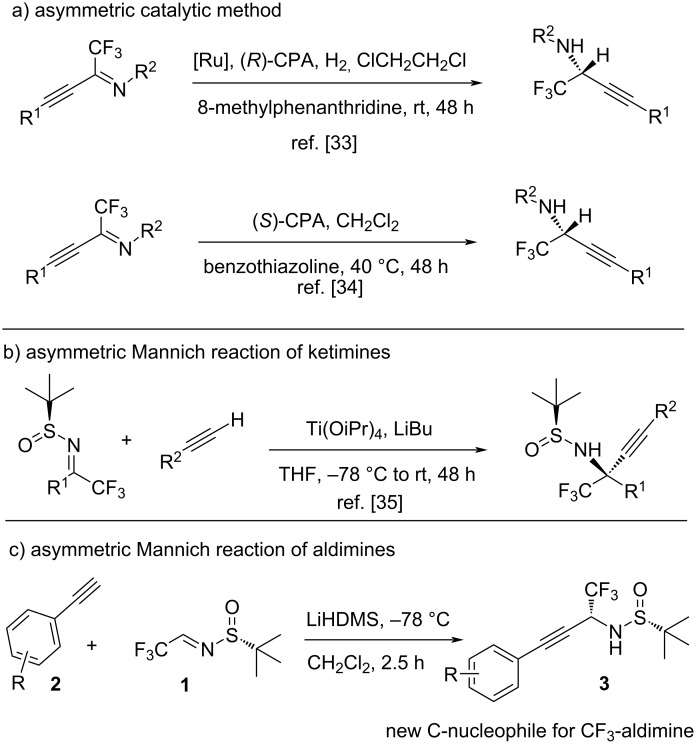
Literature-known methods (a and b) and the here reported (c) approach for the synthesis of α-trifluoromethylated α-propargylamines.

Thus, the development of synthetic methods for the preparation of these compounds, featuring trifluoromethylpropargylamine is of general research interest [28–32]. The asymmetric Ru/(*R*)-CPA-catalyzed chemoselectivie biomimetic reduction [33] and the organocatalytic transfer hydrogenation [34] of fluorinated alkynylketimines have been developed for the synthesis of fluorinated propargylamines in good yields and high enantioselectivities by the groups of Zhou and Peng, respectively (Scheme 1a). It should be mentioned that the Qing group also reported a method for the synthesis of α-trifluoromethylated α-propargylamines via a Ti-promoted addition reaction between acetylide and chiral CF_3_-ketimines (Scheme 1b) [35]. Based on our experience in the preparation of trifluoromethylated amino compounds [36–40] and the chemistry of *N*-*tert*-butylsulfinyl-3,3,3-trifluoroacetaldimine (**1)** [41–54], we noticed that the reactions of chiral imine **1** with yne nucleophiles have never been reported thus far. Accordingly, intrigued by this methodological deficiency, we decided to dedicate a special research project to this objective; herein, we report Mannich reactions between yne nucleophiles and aldimine **1** (Scheme 1c). The results reported in this work expand our knowledge of the reactivity of imines and the origins of stereocontrol, as well as provide synthetic access to a series of trifluoromethylpropargylamine of high biological interest.

## Results and Discussion

Drawing from our previous studies on the chemistry of trifluoromethylated sulfinylimine **1** [41–54], the initial reaction conditions focused on the use of 1.3 equiv of 1-ethynyl-4-methoxybenzene (**2a**) in the presence of *n*-BuLi with CH_2_Cl_2_ as a solvent at −78 °C. The desired product **3a** was obtained after 2.5 hours in 46% yield as a mixture of two diastereomers (55:45 dr, Table 1, entry 1). Fortunately, the diastereomeric products can be quite easily separated by regular column chromatography using hexane/EtOAc (4:1, v/v) as an eluent. Further optimization of the reaction conditions was then carried out to improve both the yield and diastereoselectivity. First, a series of pilot experiments in dichloromethane were performed to scan the base in the reaction. When using LDA and LiHMDS instead of *n*-BuLi, the yields were noticeably increased to 83% and 87%, respectively (Table 1, entries 2 and 3). In particular, the use of LiHMDS resulted in improved diastereoselectivity (69:31 dr, Table 1, entry 3). On the other hand, the solvent was found to show a significant effect on this transformation (Table 1, entries 4–6) and the results indicated dichloromethane was the best choice. Variation of the loading amount of 1-ethynyl-4-methoxybenzene (**2a**) did not provide any improvement on the chemical yield and the stereochemical outcome (Table 1, entries 7 and 8). We also observed a noticeable temperature effect on the reaction yield (Table 1, entries 9 and 10) and −78 °C was proved to be the best choice. Screening of the reaction time revealed that 2.5 hours were necessary for the completion of this transformation (Table 1, entries 11 and 12). Finally, we found that the use of Lewis acids BF_3_·Et_2_O and Ti(OiPr)_4_ as additives for this reaction was unsuccessful and the same level of chemical yield and diastereoselectivity was observed (Table 1, entries 13 and 14).

**Table 1:** Optimization of reaction conditions.^a^

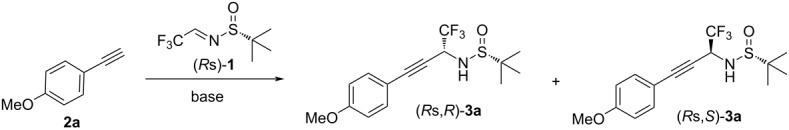							

Entry	Base	Solvent	**2a** (equiv)	*T* (°C)	Time (h)	Yield^b^ (%)	dr^c^

1	*n*-BuLi	CH_2_Cl_2_	1.3	−78	2.5	46	55:45
2	LDA	CH_2_Cl_2_	1.3	−78	2.5	83	66:34
3	LiHMDS	CH_2_Cl_2_	1.3	−78	2.5	87	69:31
4	LiHMDS	THF	1.3	−78	2.5	57	56:44
5	LiHMDS	CHCl_3_	1.3	−60	2.5	trace	–
6	LiHMDS	PhCH_3_	1.3	−78	2.5	45	63:37
7	LiHMDS	CH_2_Cl_2_	1.1	−78	2.5	85	66:34
8	LiHMDS	CH_2_Cl_2_	1.6	−78	2.5	79	68:32
9	LiHMDS	CH_2_Cl_2_	1.3	0	2.5	52	68:32
10	LiHMDS	CH_2_Cl_2_	1.3	rt	2.5	38	63:37
11	LiHMDS	CH_2_Cl_2_	1.3	−78	1.0	62	67:33
12	LiHMDS	CH_2_Cl_2_	1.3	−78	1.5	82	67:33
13^d^	LiHMDS	CH_2_Cl_2_	1.3	−78	2.5	65	66:34
14^e^	LiHMDS	CH_2_Cl_2_	1.3	−78	2.5	66	70:30

^a^Reaction conditions: **1** (0.3 mmol), base (1.3 equiv of **2a**), solvent (3 mL), under nitrogen. ^b^Isolated yield of two isomers. ^c^Determined by ^19^F NMR. ^d^0.5 equiv of BF_3_·Et_2_O was added. ^e^0.5 equiv of Ti(OiPr)_4_ was added.

**Scheme 2:**
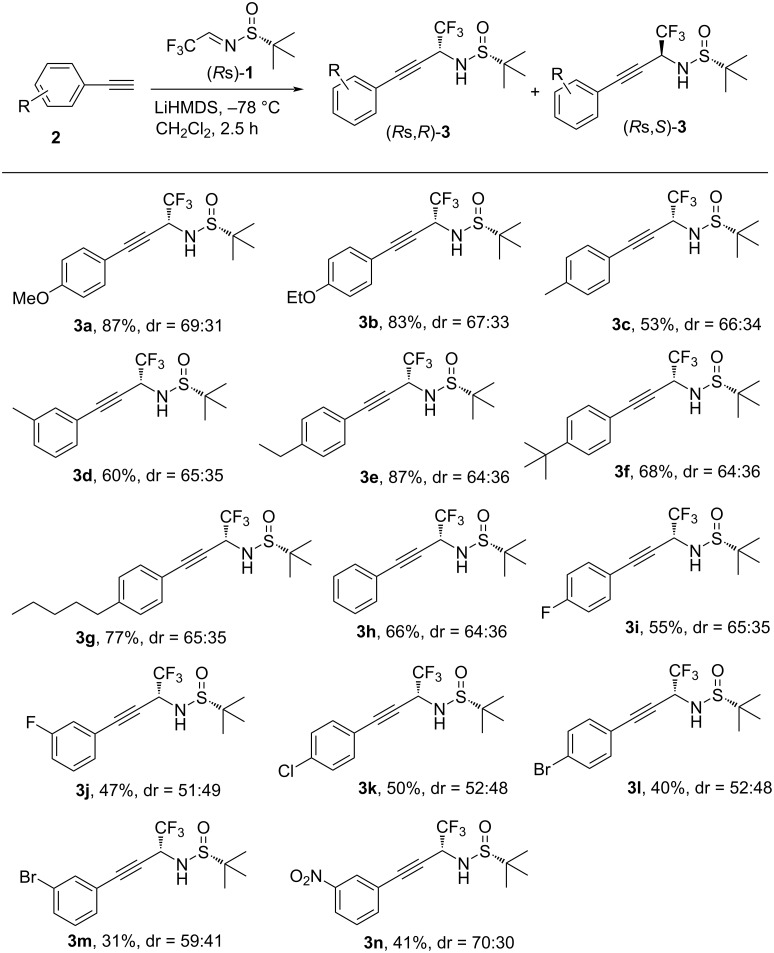
Substrate scope study. Reaction conditions: arylethyne **2** (0.39 mmol), imine **1** (0.3 mmol), LiHMDS (0.51 mmol), CH_2_Cl_2_ (3 mL), −78 °C, under nitrogen, 2.5 h. Isolated yields of mixture of isomers. Diastereoselectivities were determined by ^19^F NMR.

With the optimized reaction conditions in hand, we examined the generality of these asymmetric Mannich reactions by using various arylethynes **2** (Scheme 2). Under standard reaction conditions, all the tested substrates worked well to generate the corresponding Mannich adducts in moderate to good yields. In particular, ethynylbenzenes bearing electron-donating substituents on the aromatic ring were more compatible with this reaction, resulting in higher chemical yields (53–87%) and better diastereoselectivities (**3a**–**g**). The ethynylbenzene **2g** featuring a long alkyl chain on *para*-position was also a suitable substrate, which was converted into product **3g** in 77% yield. On the other hand, arylethynes bearing electron-withdrawing groups on the aromatic ring showed lower reactivity along with lower yields (31–55%) as well as poorer diastereoselectivities (**3i**–**m**), except for **3n** which gave 70:30 dr. It should be mentioned that the position of substituents showed almost no effects on the reaction efficiency (**3c** vs **3d**, **3i** vs **3j**, **3l** vs **3m**). Also, importantly, two diastereomers, in all the cases **3a**–**n**, can be readily obtained in diastereomerically pure form by routine column chromatography, underscoring the value of this strategy for the synthesis of chiral α-trifluoromethylated propargylamines.

**Figure 2:**
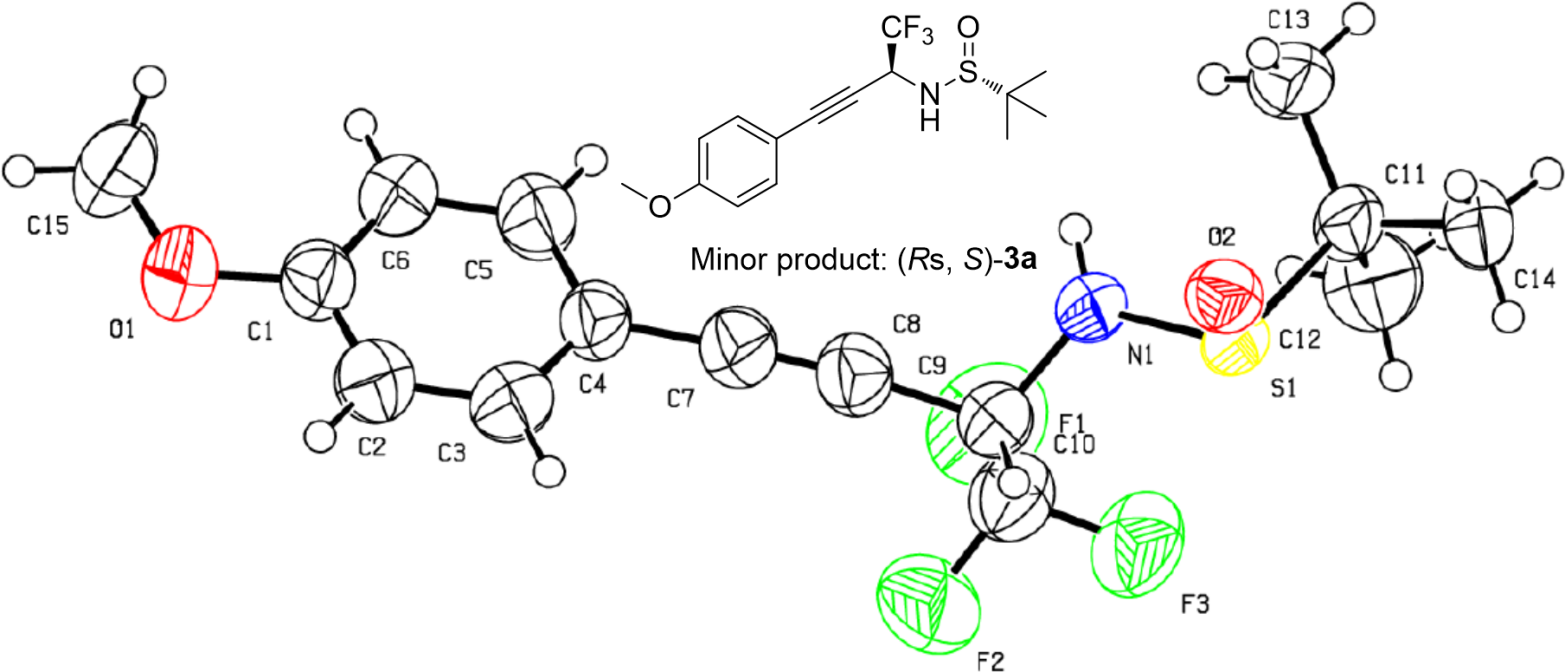
ORTEP diagram showing of the minor product of **3a**.

In order to determine the absolute configuration of the chiral addition products, we successfully performed a crystallographic analysis of the minor product **3a**. The structure is shown in Figure 2, the absolute configuration of the newly generated chiral center of the minor product **3a** is *S*, which demonstrates accordingly that the major one is *R*. The absolute configurations of other corresponding products were assigned by analogy.

**Figure 3:**
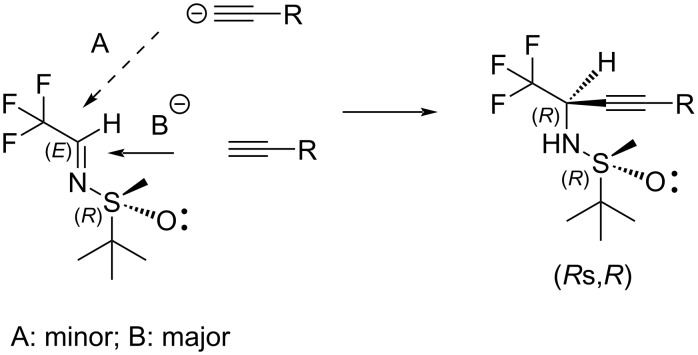
Mode of nucleophilic attacks A and B.

In general, the low-to-moderate diastereoselectivity observed in this study was unexpected. In fact, considering the excellent level (>90% de) of stereocontrol reported for the Mannich additions of aldimine **1** with sp^3^ [42,55] and sp^2^ [56] nucleophiles, including lithiated aromatics [57] one would not anticipate such a dramatic drop in the selectivity in the reactions of sp nucleophiles. Accordingly, we considered the mode of nucleophilic attack on the imine double bond in **1**. As presented in Figure 3, imine **1** might react in the most stable conformation with the bulky *tert*-butyl group pointing away from the imine double bond. In this case the only stereocontrolling factor in play is the difference between the sulfinyl oxygen and the oxygen electron lone pair. The latter presents a lesser steric obstacle rendering the corresponding nucleophilic attack the more probable event.

These ever-unexpected results strongly suggest that the origin of the stereocontrol in the reactions of imine **1** is not just the bulk of the *tert*-butyl group but, as a major factor, the corresponding chelated transition states.

**Scheme 3:**
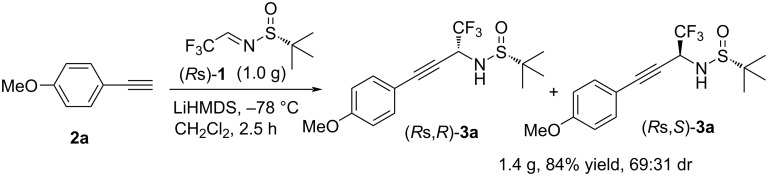
Large-scale application of the reaction.

**Scheme 4:**
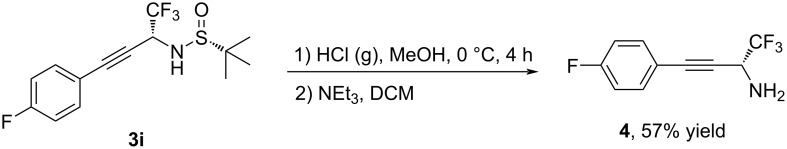
Removal of the chiral auxiliary.

As all diastereomers **3** can be easily isolated in optical pure form by routine column chromatography, this reaction should be of certain value for biological studies. In this regard, we decided to demonstrate the reproducibility of this method for large-scale synthesis (Scheme 3) and the removal of the sulfinyl auxiliary to give the free amine (Scheme 4). To our delight, 1.4 g of the desired product **3a** was obtained when the amount of sulfinylimine **1** was raised to 1.0 g (5.0 mmol). Comparing with reaction of 0.3 mmol scale, only a slight decrease in the yield was observed (84%) along with the same stereochemical outcome. Also, the chiral sulfinyl auxiliary can be easily removed under acidic conditions to give the free amine. Treating **3i** with HCl gas in methanol at 0 °C, the *tert*-butylsulfinyl group was cleaved under mild conditions and the target primary amine **4** was obtained with 57% yield.

## Conclusion

In conclusion, we have explored arylethynes as new nucleophiles for the Mannich reactions of (*R*)-*N*-*tert*-butylsulfinyl-3,3,3-trifluoroacetaldimine. Quite unexpectedly, the diastereoselectivity of the reactions were noticeably lower when compared with the corresponding reactions of sp^3^ and sp^2^ nucleophiles. Nevertheless, the two diastereomers can be easily isolated in diastereomerically pure state by regular column chromatography. This method provides access to chiral trifluoromethylpropargylamines. The application of these compounds for the synthesis of biologically relevant targets and their self-disproportionation of enantiomers (SDE) properties [58–60] are currently under study and will be reported in due course.

## Experimental

**General procedure for the Mannich reaction of arylethynes with sulfinylimine:** Into an oven-dried reaction vial flushed with N_2_ were taken the respective arylethyne **2** (0.39 mmol) and anhydrous CH_2_Cl_2_ (2.0 mL). The reaction vial was cooled to −78 °C and LiHMDS (1 M in THF, 0.51 mmol) was added dropwise with stirring. After 1 h at −78 °C, sulfinylimine **1** (0.3 mmol), dissolved in anhydrous CH_2_Cl_2_ (1.0 mL), was added dropwise. Stirring was continued at −78 °C for 2.5 h, then the reaction was quenched with saturated NH_4_Cl (2.0 mL), followed by H_2_O (5.0 mL) and the mixture was brought to room temperature. The organic layer was taken and the aqueous layer was extracted with CH_2_Cl_2_ (3 × 15 mL). The combined organic layers were dried with anhydrous Na_2_SO_4_, filtered and the solvent was removed to give the crude product **3**, which was purified by column chromatography using hexane/EtOAc (4:1, v/v) as eluent.

## Supporting Information

File 1 Experimental details and spectral data.
