# Supplementary material for: Asymmetric Mannich reactions of (S)-N-tert-butylsulfinyl-3,3,3-trifluoroacetaldimines with yne nucleophiles
Source: Beilstein J Org Chem. 2020 Oct 29;16:2671–8. doi: 10.3762/bjoc.16.217 (PMC7607431; doi:10.3762/bjoc.16.217)

BEILSTEIN JOURNAL OF ORGANIC CHEMISTRY

Supporting Information

for

Asymmetric Mannich reactions of (*S*)-*N*-*tert*-butylsulfinyl-3,3,3-trifluoroacetaldimines with yne nucleophiles

Ziyi Li, Li Wang, Yunqi Huang, Haibo Mei, Hiroyuki Konno, Hiroki Moriwaki, Vadim A. Soloshonok and Jianlin Han

Beilstein J. Org. Chem. **2020,** *16,* 2671–2678. doi:10.3762/bjoc.16.217

Experimental details and spectral data

License and Terms: This is a supporting information file under the terms of the Creative Commons Attribution License (https://creativecommons.org/licenses/by/4.0). Please note that the reuse, redistribution and reproduction in particular requires that the authors and source are credited.
The license is subject to the *Beilstein Journal of Organic Chemistry* terms and conditions: (https://www.beilstein-journals.org/bjoc)

Table of Contents

1. General information……………………………………………………………..S2

2. Experiment procedures…………………………………………………………..S2

3. Characterization data of compounds 3 and 4…..………………………………S3

4. X-ray crystallography of (*R*$_s$,*S*)-3a……………………………………………..S13

5. ^{1}H, ^{13}C and ^{19}F NMR spectra of compounds 3 and 4…………..………………S14

1. General information

All commercial reagents, including solvents were used directly without further purification. All the experiments were monitored by thin-layer chromatography (TLC) with UV light. The TLC employed 0.25 mm silica gel coated on glass plates. Column chromatography was performed with silica gel 60 (300-400 mesh). NMR spectra were recorded on Bruker 400 MHz and 600 MHz spectrometers. High resolution mass spectra (HRMS) were measured on an Agilent 6210 ESI/TOF MS instrument. Values of optical rotation were measured on an automatic polarimeter SGW-531.

2. Experiment procedures

2.1 Reaction of various phenylacetylenes with sulfinylimine

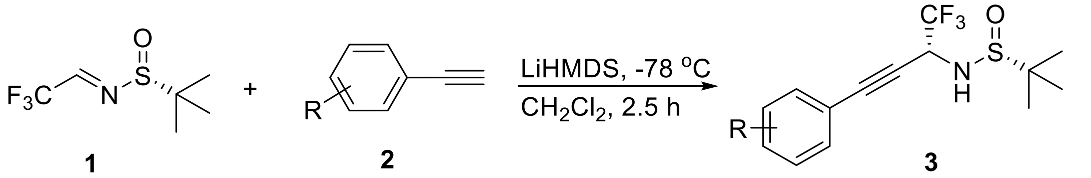

In a similar manner as described in [45] into an oven-dried reaction vial flushed with N_2 were taken compound **2** (0.39 mmol) and anhydrous CH_2Cl_2 (2.0 mL). The reaction vial was cooled to −78 °C and LiHMDS (1 M in THF, 0.51 mmol) was added dropwise with stirring. After 1 h at −78 °C, sulfinylimine **1** (0.3 mmol) dissolved in anhydrous CH_2Cl_2 (1.0 mL) was added dropwise. Stirring was continued at −78 °C for 2.5 h. Then the reaction was quenched with saturated NH_4Cl (2.0 mL), followed by H_2O (5.0 mL) and the mixture was brought to room temperature. The organic layer was taken and the aqueous layer was extracted with CH_2Cl_2 (3 × 15 mL). The combined organic layers were dried with anhydrous Na_2SO_4, filtered and the solvent was removed to give the crude product, which was purified by column chromatography using hexane/EtOAc (4:1, v/v) as eluent.

2.2 Large-scale synthesis

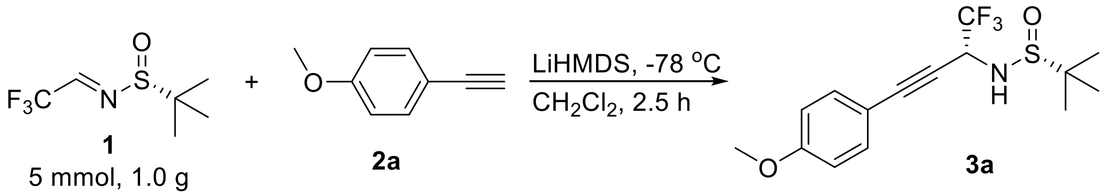

In a similar manner as described in [43] into an oven-dried reaction vial flushed with N_2 were taken compound **2a** (6.5 mmol) and anhydrous CH_2Cl_2 (25 mL). The reaction vial was cooled to −78 °C

and LiHMDS (1 M in THF, 8.5 mmol) was added dropwise with stirring. After 1 h at −78 °C, sulfinylimine **1** (5.0 mmol) dissolved in anhydrous CH_2Cl_2 (10 mL) was added dropwise. Stirring was continued at −78 °C for 2.5 h. Then the reaction was quenched with saturated NH_4Cl (15 mL), followed by H_2O (20 mL) and the mixture was brought to room temperature. The organic layer was taken and the aqueous layer was extracted with CH_2Cl_2 (3 × 30 mL). The combined organic layers were dried with anhydrous Na_2SO_4, filtered and the solvent was removed to give the crude product, which was purified by column chromatography using hexane/EtOAc (4:1, v/v) as eluent.

2.3 Cleavage of the chiral auxiliary

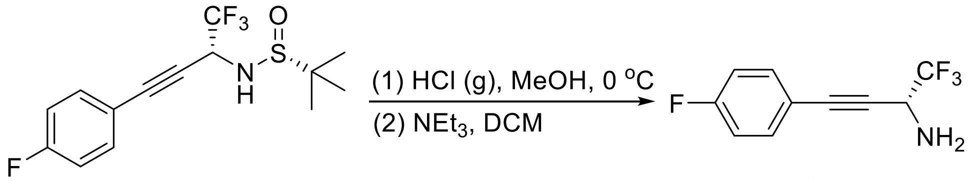

3i **4**

In a similar manner as described in [43] compound **3i** (0.3 mmol) and MeOH (10 mL) were placed in a three-necked flask and cooled to 0 °C. HCl gas was bubbled into the mixture for 10 minutes and the reaction was stirred at 0 °C for 4 h. Volatiles were removed under reduced pressure. The residue was dissolved in CH_2Cl_2 (10 mL), followed by Et_3N to adjust the pH > 9. Then, H_2O (10 mL) was added. The organic layer was taken, washed with H_2O (3 × 10 mL), dried with anhydrous Na_2SO_4, filtered and the solvent was removed to give the crude product, which was purified by column chromatography using hexane/EtOAc (8:1, v/v) as eluent.

3. Characterization data of compounds 3 and 4

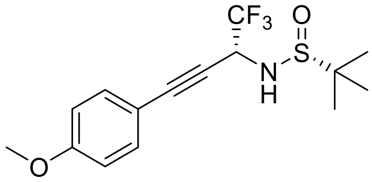

Compound (R_s, R)-**3a**: white solid, mp = 88-89 °C, $[\alpha]_D^{25}$ = -50.9 (c = 0.06, CH_2Cl_2). ^{1}H NMR (600 MHz, $CDCl_3$): δ = 7.47 (d, J = 8.82 Hz, 2H), 6.88 (d, J = 8.82 Hz, 2H), 4.89-4.85 (m, 1H), 4.44 (d, J = 7.32 Hz, 1H), 3.83 (s, 3H), 1.29 (s, 9H). ^{13}C NMR (100 MHz, $CDCl_3$): δ = 160.4, 133.6, 127.3 (q, J = 279.7 Hz), 114.0, 113.1, 89.2, 77.3, 56.8, 55.3, 51.2 (q, J = 34.6 Hz), 22.4. ^{19}F NMR (376 MHz, $CDCl_3$): δ = -76.0. HRMS (ESI): calculated for $C_{15}H_{19}F_3NO_2S^+$ $[M+H]^+$ 334.1083, found

334.1082.

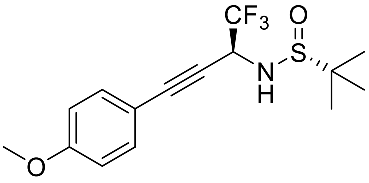

Compound (R_s, S)-**3a**: white solid, mp = 123-125 °C, $[\alpha]_D^{25}$ = -16.8 (c = 0.06, CH_2Cl_2). ^{1}H NMR (600 MHz, $CDCl_3$): δ = 7.44 (d, J = 8.58 Hz, 2H), 6.86 (d, J = 8.52 Hz, 2H), 4.79-4.75 (m, 1H), 3.83 (s, 3H), 3.78 (d, J = 7.32 Hz, 1H), 1.28 (s, 9H). ^{13}C NMR (100 MHz, $CDCl_3$): δ = 160.4, 133.6, 127.3 (q, J = 279.4 Hz), 114.0, 113.0, 87.8, 78.3, 57.3, 55.3, 52.2 (q, J = 34.7 Hz), 22.4. ^{19}F NMR (376 MHz, $CDCl_3$): δ = -76.4.

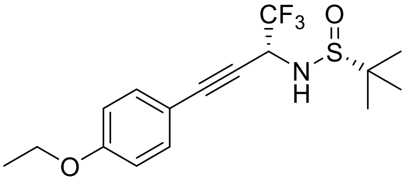

Compound (R_s, R)-**3b**: white solid, mp = 95-96 °C, $[\alpha]_D^{25}$ = -77.3 (c = 0.08, CH_2Cl_2). ^{1}H NMR (600 MHz, $CDCl_3$): δ = 7.42 (d, J = 8.82 Hz, 2H), 6.85 (d, J = 8.82 Hz, 2H), 4.79-4.75 (m, 1H), 4.06 (q, J = 6.96 Hz, 2H), 3.80 (d, J = 7.26 Hz, 1H), 1.44 (q, J = 6.96 Hz, 3H), 1.28 (s, 9H). ^{13}C NMR (150 MHz, $CDCl_3$): δ = 159.8, 133.6, 125.9 (q, J = 279.3 Hz), 114.5, 112.7, 87.9, 78.2 (d, J = 2.4 Hz), 63.5, 57.3, 52.0 (q, J = 34.8 Hz), 22.4, 14.7. ^{19}F NMR (565 MHz, $CDCl_3$): δ = -76.4. HRMS (ESI): calculated for $C_{16}H_{21}F_3NO_2S^+$ $[M+H]^+$ 348.1240, found 348.1241.

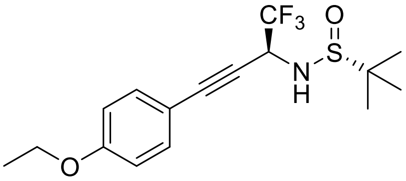

Compound (R_s, S)-**3b**: yellow solid, mp = 78-79 °C, $[\alpha]_D^{25}$ = -20.6 (c = 0.06, CH_2Cl_2). ^{1}H NMR (600 MHz, $CDCl_3$): δ = 7.42 (d, J = 8.76 Hz, 2H), 6.85 (d, J = 8.76 Hz, 2H), 4.79-4.75 (m, 1H), 4.06 (q, J = 6.96 Hz, 2H), 3.80 (d, J = 7.26 Hz, 1H), 1.44 (q, J = 7.02 Hz, 3H), 1.28 (s, 9H). ^{13}C NMR (150 MHz, $CDCl_3$): δ = 159.8, 133.6, 125.9 (q, J = 279.6 Hz), 114.5, 112.9, 89.3, 63.6, 56.8, 53.4, 51.0 (q, J = 34.8 Hz), 22.5, 14.7. ^{19}F NMR (565 MHz, $CDCl_3$): δ = -76.4.

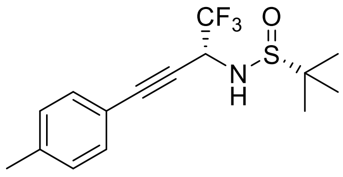

Compound (R_s, R)-**3c**: yellow solid, mp = 91-92 °C, $[\alpha]_D^{25}$ = -48.1 (c = 0.06, CH_2Cl_2). ^{1}H NMR (400 MHz, $CDCl_3$): δ = 7.43 (d, J = 8.2 Hz, 2H), 7.17 (d, J = 7.88 Hz, 2H), 4.90-4.84 (m, 1H), 4.32 (b, 1H), 2.38 (s, 3H), 1.29 (s, 9H). ^{13}C NMR (100 MHz, $CDCl_3$): δ = 139.7, 132.0, 129.1, 127.3 (q, J = 279.7 Hz), 118.0, 89.3, 77.9, 56.9, 51.2 (q, J = 34.6 Hz), 22.4, 21.6. ^{19}F NMR (565 MHz, $CDCl_3$): δ = -75.9. HRMS (ESI): calculated for $C_{15}H_{19}F_3NOS^+$ $[M+H]^+$ 318.1134, found 318.1133.

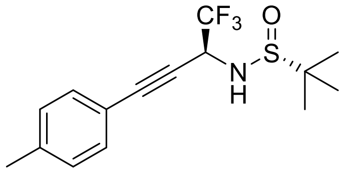

Compound (R_s, S)-**3c**: white solid, mp = 121-123 °C, $[\alpha]_D^{25}$ = -28.5 (c = 0.06, CH_2Cl_2). ^{1}H NMR (400 MHz, $CDCl_3$): δ = 7.40 (d, J = 8.16 Hz, 2H), 7.16 (d, J = 7.88 Hz, 2H), 4.81-4.75 (m, 1H), 3.86 (d, J = 7.4 Hz, 1H), 2.37 (s, 3H), 1.29 (s, 9H). ^{13}C NMR (100 MHz, $CDCl_3$): δ = 139.7, 132.0, 129.1, 127.3 (q, J = 279.3 Hz), 117.9, 88.0, 78.9 (d, J = 2.3 Hz), 57.4, 52.2 (q, J = 34.8 Hz), 22.4, 21.6. ^{19}F NMR (565 MHz, $CDCl_3$): δ = -76.4.

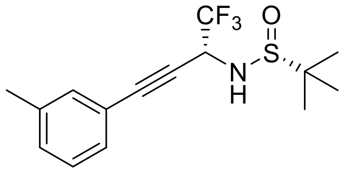

Compound (R_s, R)-**3d**: white solid, mp = 90-91 °C, $[\alpha]_D^{25}$ = -58.1 (c = 0.07, CH_2Cl_2). ^{1}H NMR (600 MHz, $CDCl_3$): δ = 7.36-7.33 (m, 2H), 7.25 (t, J = 7.56 Hz, 1H), 7.21-7.20 (m, 1H), 4.91-4.87 (m, 1H), 4.36 (d, J = 7.38 Hz, 1H), 2.35 (s, 3H), 1.29 (s, 9H). ^{13}C NMR (150 MHz, $CDCl_3$): δ = 138.2, 132.5, 130.3, 129.1, 128.3, 124.0 (d, J = 279.6 Hz), 120.9, 100.0, 89.3, 56.9, 50.9 (q, J = 34.7 Hz), 22.4, 21.1. ^{19}F NMR (565 MHz, $CDCl_3$): δ = -76.0. HRMS (ESI): calculated for $C_{15}H_{19}F_3NOS^+$ $[M+H]^+$ 318.1134, found 318.1134.

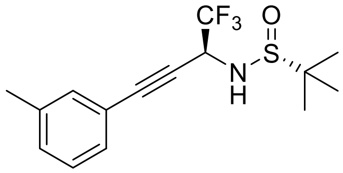

Compound (R_s, S)-**3d**: white solid, mp = 70-71 °C, $[\alpha]_D^{25}$ = -24.9 (c = 0.06, CH_2Cl_2). ^{1}H NMR (600 MHz, $CDCl_3$): δ = 7.33-7.30 (m, 2H), 7.25 (t, J = 7.56 Hz, 1H), 7.21-7.19 (m, 1H), 4.80-4.76 (m, 1H), 3.79 (d, J = 7.44 Hz, 1H), 2.35 (s, 3H), 1.29 (s, 9H). ^{13}C NMR (150 MHz, $CDCl_3$): δ = 138.1, 132.6, 130.3, 129.1, 128.2, 125.9 (q, J = 279.9 Hz), 120.7, 88.0, 79.2, 57.4, 52.0 (q, J = 35.0 Hz), 22.4, 21.1. ^{19}F NMR (565 MHz, $CDCl_3$): δ = -76.4.

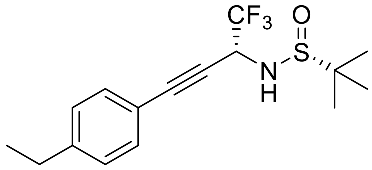

Compound (R_s, R)-**3e**: white solid, mp = 75-76 °C, $[\alpha]_D^{25}$ = -79.1 (c = 0.06, CH_2Cl_2). ^{1}H NMR (400 MHz, $CDCl_3$): δ = 7.46 (d, J = 8.16 Hz, 2H), 7.19 (d, J = 8.32 Hz, 2H), 4.92-4.85 (m, 1H), 4.41-4.35 (m, 1H), 2.70 (q, J = 7.56 Hz, 2H), 1.29 (s, 9H), 1.26 (t, J = 7.56 Hz, 3H). ^{13}C NMR (100 MHz, $CDCl_3$): δ = 146.0, 132.1, 128.0, 127.3 (q, J = 279.8 Hz), 118.3, 89.3, 78.0, 56.9, 51.2 (q, J = 34.7 Hz), 28.9, 22.4, 15.3. ^{19}F NMR (376 MHz, $CDCl_3$): δ = -76.0. HRMS (ESI): calculated for $C_{16}H_{21}F_3NOS^+$ $[M+H]^+$ 332.1290, found 332.1297.

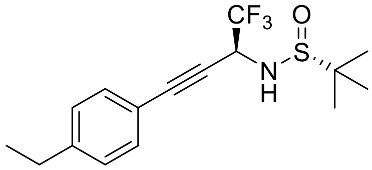

Compound (R_s, S)-**3e**: white solid, mp = 120-121 °C, $[\alpha]_D^{25}$ = -16.5 (c = 0.07, CH_2Cl_2). ^{1}H NMR (400 MHz, $CDCl_3$): δ = 7.43 (d, J = 8.24 Hz, 2H), 7.18 (d, J = 8.16 Hz, 2H), 4.82-4.75 (m, 1H), 3.84 (d, J = 7.32 Hz, 1H), 2.69 (q, J = 7.56 Hz, 2H), 1.29 (s, 9H), 1.26 (t, J = 7.6 Hz, 3H). ^{13}C NMR (100 MHz, $CDCl_3$): δ = 146.0, 132.1, 127.9, 127.3 (q, J = 279.4 Hz), 118.1, 88.0, 78.9 (d, J = 2.2 Hz), 57.4, 52.2 (q, J = 34.7 Hz), 28.9, 22.4, 15.3. ^{19}F NMR (376 MHz, $CDCl_3$): δ = -76.4.

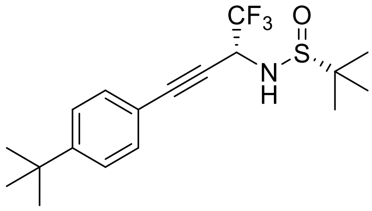

Compound (R_s, R)-**3f**: yellow solid, mp = 101-102 °C, $[\alpha]_D^{25}$ = -50.3 (c = 0.07, CH_2Cl_2). ^{1}H NMR (400 MHz, $CDCl_3$): δ = 7.49-7.45 (m, 2H), 7.39-7.36 (m, 2H), 4.92-4.85 (m, 1H), 4.44-4.36 (m, 1H), 1.33 (s, 9H), 1.29 (s, 9H). ^{13}C NMR (150 MHz, $CDCl_3$): δ = 152.8, 131.8, 125.4, 124.0 (d, J =

279.1 Hz), 118.1, 89.3 (d, J = 3.2 Hz), 78.0, 56.9, 51.0 (q, J = 34.3 Hz), 34.9, 31.1, 22.4. ^{19}F NMR (565 MHz, $CDCl_3$): δ = -76.0. HRMS (ESI): calculated for $C_{18}H_{25}F_3NOS^+$ $[M+H]^+$ 360.1603, found 360.1602.

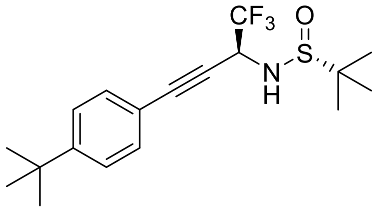

Compound (R_s, S)-**3f**: white solid, mp = 157-158 °C, $[\alpha]_D^{25}$ = -15.8 (c = 0.06, CH_2Cl_2). ^{1}H NMR (400 MHz, $CDCl_3$): δ = 7.45-7.42 (m, 2H), 7.38-7.35 (m, 2H), 4.82-4.75 (m, 1H), 3.77 (d, J = 7.24 Hz, 1H), 1.33 (s, 9H), 1.29 (s, 9H). ^{13}C NMR (150 MHz, $CDCl_3$): δ = 152.9, 131.8, 125.4, 124.0 (d, J = 279.6 Hz), 117.9, 87.9, 78.9, 57.3, 51.9 (q, J = 35.0 Hz), 34.9, 31.1, 22.4. ^{19}F NMR (565 MHz, $CDCl_3$): δ = -76.4.

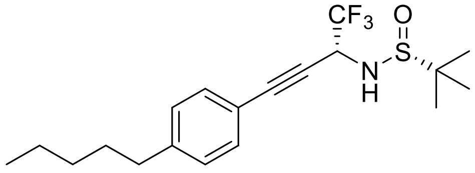

Compound (R_s, R)-**3g**: yellow solid, mp = 81-82 °C, $[\alpha]_D^{25}$ = -91.4 (c = 0.07, CH_2Cl_2). ^{1}H NMR (400 MHz, $CDCl_3$): δ = 7.46 (d, J = 8.16 Hz, 2H), 7.17 (d, J = 8.16 Hz, 2H), 4.93-4.86 (m, 1H), 4.43 (d, J = 7.32 Hz, 1H), 2.64 (t, J = 7.6 Hz, 2H), 1.65-1.58 (m, 2H), 1.36-1.30 (m, 4H), 1.29 (s, 9H), 0.92 (t, J = 6.84 Hz, 3H). ^{13}C NMR (150 MHz, $CDCl_3$): δ = 144.7, 132.0, 128.5, 125.9 (q, J = 279.5 Hz), 118.2, 89.4, 78.0, 56.8, 51.0 (q, J = 34.6 Hz), 35.9, 31.4, 30.9, 22.5, 22.4, 14.0. ^{19}F NMR (376 MHz, $CDCl_3$): δ = -76.0. HRMS (ESI): calculated for $C_{19}H_{27}F_3NOS^+$ $[M+H]^+$ 374.1760, found 374.1762.

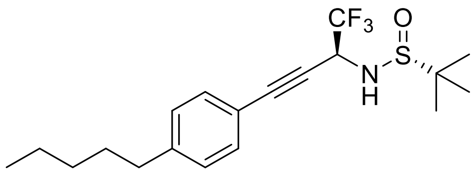

Compound (R_s, S)-**3g**: white solid, mp = 109-110 °C, $[\alpha]_D^{25}$ = -40.1 (c = 0.08, CH_2Cl_2). ^{1}H NMR (400 MHz, $CDCl_3$): δ = 7.42 (d, J = 8.2 Hz, 2H), 7.16 (d, J = 8.24 Hz, 2H), 4.82-4.75 (m, 1H), 3.78 (d, J = 7.36 Hz, 1H), 2.63 (t, J = 7.6 Hz, 2H), 1.65-1.58 (m, 2H), 1.39-1.30 (m, 4H), 1.29 (s, 9H), 0.92 (t, J = 6.8 Hz, 3H). ^{13}C NMR (150 MHz, $CDCl_3$): δ = 144.7, 132.0, 128.5, 125.9 (q, J = 279.2

Hz), 118.0, 88.0, 78.9 (d, J = 3.1 Hz), 57.3, 52.0 (q, J = 34.8 Hz), 35.9, 31.4, 30.8, 22.5, 22.4, 14.0. ^{19}F NMR (376 MHz, $CDCl_3$): δ = -76.4.

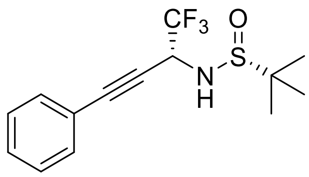

Compound (R_s, R)-**3h**: white solid, mp = 115-117 °C, $[\alpha]_D^{25}$ = -47.8 (c = 0.07, CH_2Cl_2). ^{1}H NMR (600 MHz, $CDCl_3$): δ = 7.54-7.52 (m, 2H), 7.40-7.38 (m, 1H), 7.36 (d, J = 7.68 Hz, 2H), 4.92-4.87 (m, 1H), 4.43 (d, J = 7.5 Hz, 1H), 1.29 (s, 9H). ^{13}C NMR (150 MHz, $CDCl_3$): δ = 132.0, 129.4, 128.4, 125.9 (q, J = 280.1 Hz), 121.1, 89.1, 78.6 (d, J = 2.5 Hz), 56.9, 50.9 (q, J = 34.7 Hz), 22.4. ^{19}F NMR (565 MHz, $CDCl_3$): δ = -75.9. HRMS (ESI): calculated for $C_{14}H_{17}F_3NOS^+$ [M+H]$^+$ 304.0977, found 304.0980.

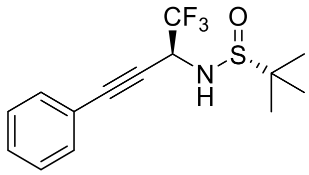

Compound (R_s, S)-**3h**: white solid, mp = 125-127 °C, $[\alpha]_D^{25}$ = -36.2 (c = 0.07, CH_2Cl_2). ^{1}H NMR (600 MHz, $CDCl_3$): δ = 7.51-7.49 (m, 2H), 7.41-7.38 (m, 1H), 7.36-7.33 (m, 2H), 4.82-4.77 (m, 1H), 3.83 (d, J = 7.44 Hz, 1H), 1.29 (s, 9H). ^{13}C NMR (150 MHz, $CDCl_3$): δ = 132.1, 129.4, 128.4, 125.8 (q, J = 279.2 Hz), 120.9, 87.6, 79.6 (d, J = 2.4 Hz), 57.4, 52.0 (q, J = 34.7 Hz), 22.4. ^{19}F NMR (565 MHz, $CDCl_3$): δ = -76.4.

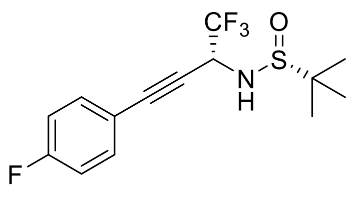

Compound (R_s, R)-**3i**: white solid, mp = 101-103 °C, $[\alpha]_D^{25}$ = -89.6 (c = 0.08, CH_2Cl_2). ^{1}H NMR (600 MHz, $CDCl_3$): δ = 7.53-7.51 (m, 2H), 7.07-7.04 (m, 2H), 4.88-4.84 (m, 1H), 4.27 (d, J = 7.26 Hz, 1H), 1.29 (s, 9H). ^{13}C NMR (150 MHz, $CDCl_3$): δ = 164.0 (d, J = 249.7 Hz), 134.1 (d, J = 8.7 Hz), 125.8 (q, J = 279.8 Hz), 117.1 (d, J = 3.4 Hz), 115.9 (d, J = 22.0 Hz), 88.1, 78.3, 56.9, 50.8 (q, J = 34.6 Hz), 22.4. ^{19}F NMR (565 MHz, $CDCl_3$): δ = -75.9, -108.9. HRMS (ESI): calculated for $C_{14}H_{16}F_4NOS^+$ [M+H]$^+$ 322.0883, found 322.0887.

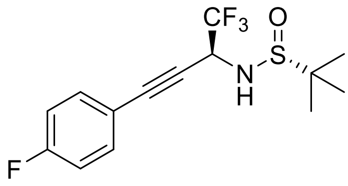

Compound (R_s, S)-**3i**: white solid, mp = 123-125 °C, $[\alpha]_D^{25}$ = -71.4 (c = 0.06, CH_2Cl_2). ^{1}H NMR (600 MHz, $CDCl_3$): δ = 7.51-7.47 (m, 2H), 7.06-7.03 (m, 2H), 4.79-4.75 (m, 1H), 3.80 (d, J = 7.62 Hz, 1H), 1.29 (s, 9H). ^{13}C NMR (150 MHz, $CDCl_3$): δ = 164.0 (d, J = 249.6 Hz), 134.2 (d, J = 8.4 Hz), 125.8 (q, J = 278.9 Hz), 117.0 (d, J = 3.6 Hz), 115.8 (d, J = 22.1 Hz), 86.8, 79.4, 57.4, 52.0 (q, J = 34.7 Hz), 22.4. ^{19}F NMR (565 MHz, $CDCl_3$): δ = -76.3, -108.9.

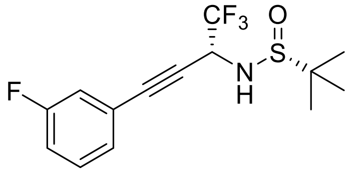

Compound (R_s, R)-**3j**: white solid, mp = 90-91 °C, $[\alpha]_D^{25}$ = -67.7 (c = 0.08, CH_2Cl_2). ^{1}H NMR (600 MHz, $CDCl_3$): δ = 7.35-7.30 (m, 2H), 7.23-7.21 (m, 1H), 7.13-7.09 (m, 1H), 4.88-4.84 (m, 1H), 4.19 (d, J = 7.32 Hz, 1H), 1.29 (s, 9H). ^{13}C NMR (150 MHz, $CDCl_3$): δ = 163.1 (d, J = 245.7 Hz), 130.1 (d, J = 8.7 Hz), 128.0 (d, J = 3.3 Hz), 123.9 (d, J = 279.7 Hz), 122.8 (d, J = 9.4 Hz), 119.0 (d, J = 23.1 Hz), 117.0 (d, J = 21.1 Hz), 100.0, 87.7 (d, J = 4.1 Hz), 57.0, 50.8 (q, J = 35.3 Hz), 22.4. ^{19}F NMR (565 MHz, $CDCl_3$): δ = -75.8, -112.4. HRMS (ESI): calculated for $C_{14}H_{16}F_4NOS^+$ $[M+H]^+$ 322.0883, found 322.0886.

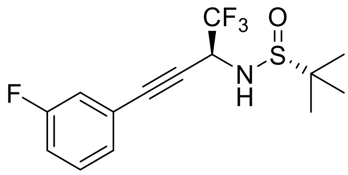

Compound (R_s, S)-**3j**: white solid, mp = 95-96 °C, $[\alpha]_D^{25}$ = -42.2 (c = 0.07, CH_2Cl_2). ^{1}H NMR (600 MHz, $CDCl_3$): δ = 7.35-7.29 (m, 2H), 7.21-7.19 (m, 1H), 7.12-7.09 (m, 1H), 4.81-4.76 (m, 1H), 3.83 (d, J = 7.74 Hz, 1H), 1.29 (s, 9H). ^{13}C NMR (150 MHz, $CDCl_3$): δ = 163.0 (d, J = 245.7 Hz), 130.1 (d, J = 8.3 Hz), 128.0 (d, J = 3.3 Hz), 123.9 (d, J = 279.6 Hz), 122.7 (d, J = 9.5 Hz), 119.0 (d, J = 23.1 Hz), 117.0 (d, J = 20.9 Hz), 86.4, 80.5, 57.4, 52.0 (q, J = 35.0 Hz), 22.4. ^{19}F NMR (565 MHz, $CDCl_3$): δ = -76.3, -112.4.

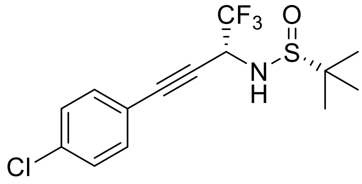

Compound (R_s, R)-**3k**: white solid, mp = 83-85 °C, $[\alpha]_D^{25}$ = -98.4 (c = 0.08, CH_2Cl_2). ^{1}H NMR (600 MHz, $CDCl_3$): δ = 7.47 (d, J = 8.46 Hz, 2H), 7.34 (d, J = 8.46 Hz, 2H), 4.88-4.84 (m, 1H), 4.23 (d, J = 7.32 Hz, 1H), 1.29 (s, 9H). ^{13}C NMR (150 MHz, $CDCl_3$): δ = 135.7, 133.3, 128.8, 123.9 (d, J = 279.4 Hz), 119.5, 88.0, 79.5 (d, J = 2.5 Hz), 57.0, 50.8 (q, J = 35.0 Hz), 22.4. ^{19}F NMR (565 MHz, $CDCl_3$): δ = -75.8. HRMS (ESI): calculated for $C_{14}H_{16}ClF_3NOS^+$ $[M+H]^+$ 338.0588, found 338.0592.

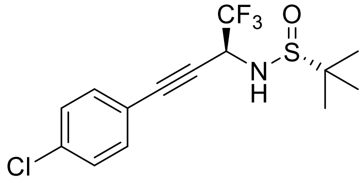

Compound (R_s, S)-**3k**: white solid, mp = 144-146 °C, $[\alpha]_D^{25}$ = -71.4 (c = 0.06, CH_2Cl_2). ^{1}H NMR (600 MHz, $CDCl_3$): δ = 7.44 (d, J = 8.52 Hz, 2H), 7.34 (d, J = 8.52 Hz, 2H), 4.80-4.75 (m, 1H), 3.82 (d, J = 7.74 Hz, 1H), 1.29 (s, 9H). ^{13}C NMR (150 MHz, $CDCl_3$): δ = 135.7, 133.3, 128.8, 123.9 (d, J = 279.2 Hz), 119.4, 100.0, 86.7, 57.4, 52.0 (q, J = 34.8 Hz), 22.4. ^{19}F NMR (565 MHz, $CDCl_3$): δ = -76.3.

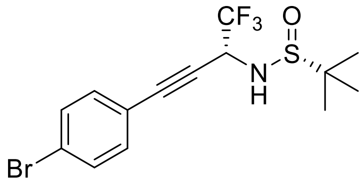

Compound (R_s, R)-**3l**: white solid, mp = 62-63 °C, $[\alpha]_D^{25}$ = -140.4 (c = 0.08, CH_2Cl_2). ^{1}H NMR (400 MHz, $CDCl_3$): δ = 7.51-7.48 (m, 2H), 7.40-7.37 (m, 2H), 4.88-4.81 (m, 1H), 4.22 (d, J = 7.32 Hz, 1H), 1.29 (s, 9H). ^{13}C NMR (150 MHz, $CDCl_3$): δ = 133.4, 131.7, 123.9, 123.8 (d, J = 279.9 Hz), 120.0, 88.0, 79.7, 57.0, 50.8 (q, J = 35.0 Hz), 22.4. ^{19}F NMR (565 MHz, $CDCl_3$): δ = -75.8. HRMS (ESI): calculated for $C_{14}H_{16}BrF_3NOS^+$ $[M+H]^+$ 382.0083, found 382.0077.

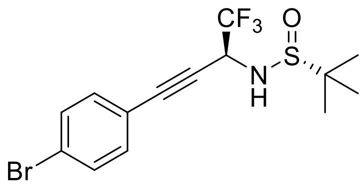

Compound (R_s, S)-**3l**: white solid, mp = 132-134 ºC, $[\alpha]_D^{25}$ = -33.3 (c = 0.06, CH_2Cl_2). ^1H NMR (400 MHz, $CDCl_3$): δ = 7.51-7.47 (m, 2H), 7.38-7.35 (m, 2H), 4.80-4.74 (m, 1H), 3.85 (d, J = 7.84 Hz, 1H), 1.28 (s, 9H). ^{13}C NMR (150 MHz, $CDCl_3$): δ = 133.5, 131.7, 123.9, 123.8 (d, J = 279.6 Hz), 119.9, 86.7, 80.7(d, J = 2.5 Hz), 57.4, 52.0 (q, J = 34.9 Hz), 22.4. ^{19}F NMR (565 MHz, $CDCl_3$): δ = -76.3.

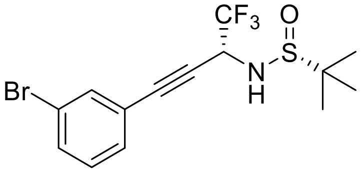

Compound (R_s, R)-**3m**: yellow oil, $[\alpha]_D^{25}$ = -14.2 (c = 0.08, CH_2Cl_2). ^1H NMR (400 MHz, $CDCl_3$): δ = 7.68-7.67 (m, 1H), 7.55-7.52 (m, 1H), 7.48-7.45 (m, 1H), 7.25 (t, J = 7.88 Hz, 1H), 4.89-4.82 (m, 1H), 4.20 (d, J = 7.28 Hz, 1H), 1.29 (s, 9H). ^{13}C NMR (150 MHz, $CDCl_3$): δ = 134.7, 132.7, 130.7. 129.9, 123.9 (d, J = 279.6 Hz), 123.0, 122.2, 87.4, 79.8, 57.0, 50.8 (q, J = 34.7 Hz), 22.4. ^{19}F NMR (565 MHz, $CDCl_3$): δ = -75.8. HRMS (ESI): calculated for $C_{14}H_{16}BrF_3NOS^+$ $[M+H]^+$ 382.0083, found 382.0076.

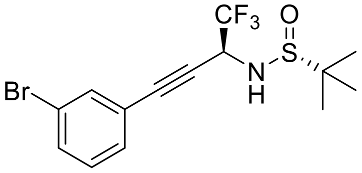

Compound (R_s, S)-**3m**: white solid, mp = 50-51 ºC, $[\alpha]_D^{25}$ = -82.1 (c = 0.04, CH_2Cl_2). ^1H NMR (400 MHz, $CDCl_3$): δ = 7.66-7.65 (m, 1H), 7.54-7.52 (m, 1H), 7.44-7.42 (m, 1H), 7.24 (t, J = 7.92 Hz, 1H), 4.81-4.74 (m, 1H), 3.86 (d, J = 7.84 Hz, 1H), 1.29 (s, 9H). ^{13}C NMR (150 MHz, $CDCl_3$): δ = 134.8, 132.7, 130.6. 129.8, 123.9 (d, J = 279.3 Hz), 122.9, 122.2, 86.2, 80.8, 57.5, 52.0 (q, J = 34.9 Hz), 22.4. ^{19}F NMR (565 MHz, $CDCl_3$): δ = -76.3.

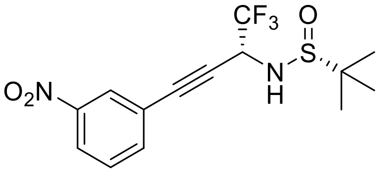

Compound (R_s, R)-**3n**: white solid, mp = 75-76 ºC, $[\alpha]_D^{25}$ = -88.6 (c = 0.08, CH_2Cl_2). ^1H NMR (600 MHz, $CDCl_3$): δ = 8.37-8.36 (m, 1H), 8.26-8.24 (m, 1H), 7.86-7.84 (m, 1H), 7.58 (t, J = 8.04 Hz, 1H), 4.93-4.89 (m, 1H), 4.37 (d, J = 7.38 Hz, 1H), 1.30 (s, 9H). ^{13}C NMR (150 MHz, $CDCl_3$): δ = 148.1, 137.8, 129.6. 126.9, 125.7 (q, J = 280.2 Hz), 124.2, 122.8, 86.4, 81.2, 57.1, 50.6 (q, J = 35.2

Hz), 22.4. ^{19}F NMR (565 MHz, CDCl$_3$): δ = -75.5. HRMS (ESI): calculated for $C_{14}H_{16}F_3N_2O_3S^+$ $[M+H]^+$ 349.0828, found 349.0832.

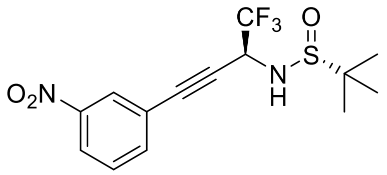

Compound (R_s, S)-**3n**: white solid, mp = 100-102 °C, $[\alpha]_D^{25}$ = -46.0 (c = 0.06, CH$_2$Cl$_2$). ^{1}H NMR (600 MHz, CDCl$_3$): δ = 8.36-8.35 (m, 1H), 8.27-8.25 (m, 1H), 7.83-7.81 (m, 1H), 7.58 (t, *J* = 8.04 Hz, 1H), 4.84-4.79 (m, 1H), 3.81 (d, *J* = 8.1 Hz, 1H), 1.31 (s, 9H). ^{13}C NMR (150 MHz, CDCl$_3$): δ = 148.0, 137.7, 129.5. 126.9, 124.2, 123.8 (d, *J* = 279.5 Hz), 122.7, 85.2, 82.2, 57.6, 52.0 (q, *J* = 35.0 Hz), 22.4. ^{19}F NMR (565 MHz, CDCl$_3$): δ = -76.1.

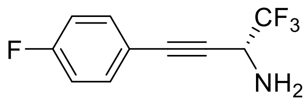

Compound (R)-**4**: colorless oil, $[\alpha]_D^{25}$ = +27.6 (c = 0.06, CH$_2$Cl$_2$). ^{1}H NMR (400 MHz, CDCl$_3$): δ = 7.48-7.45 (m, 2H), 7.06-7.02 (m, 2H), 4.32-4.28 (m, 1H), 1.81 (b, 2H). ^{13}C NMR (150 MHz, CDCl$_3$): δ = 163.7 (d, *J* = 248.9 Hz), 133.9 (d, *J* = 8.6 Hz), 126.9 (q, *J* = 279.1 Hz), 117.7 (d, *J* = 3.7 Hz), 115.8 (d, *J* = 22.0 Hz), 84.3, 81.9, 48.1 (q, *J* = 34.1 Hz). ^{19}F NMR (565 MHz, CDCl$_3$): δ = -78.2, -109.7. HRMS (ESI): calculated for $C_{10}H_8F_4N^+$ $[M+H]^+$ 218.0587, found 218.0589.

4. X-ray crystallography of (**_R_**s,**_S_**)-3a

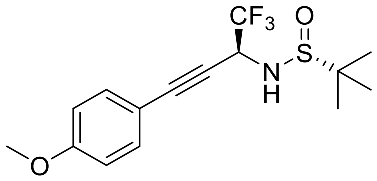

Minor product: (*R*s, *S*)-**3a**

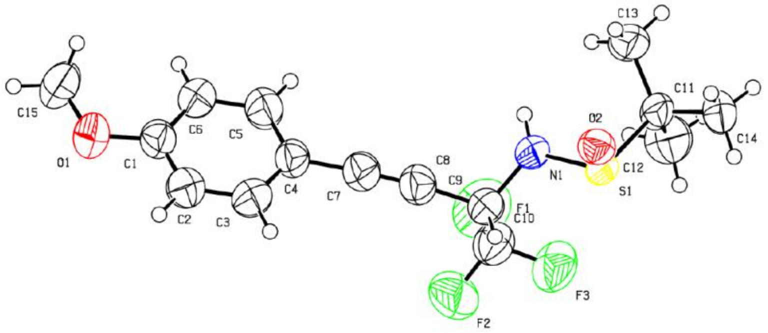

Figure S1. ORTEP diagram showing of (R_s, *S*)-**3a** (CCDC number 2010727)

5. ^{1}H, ^{13}C and ^{19}F NMR spectra of compounds 3 and 4

^{1}H NMR (600 MHz, $CDCl_3$) of (R_s, R)-**3a**:

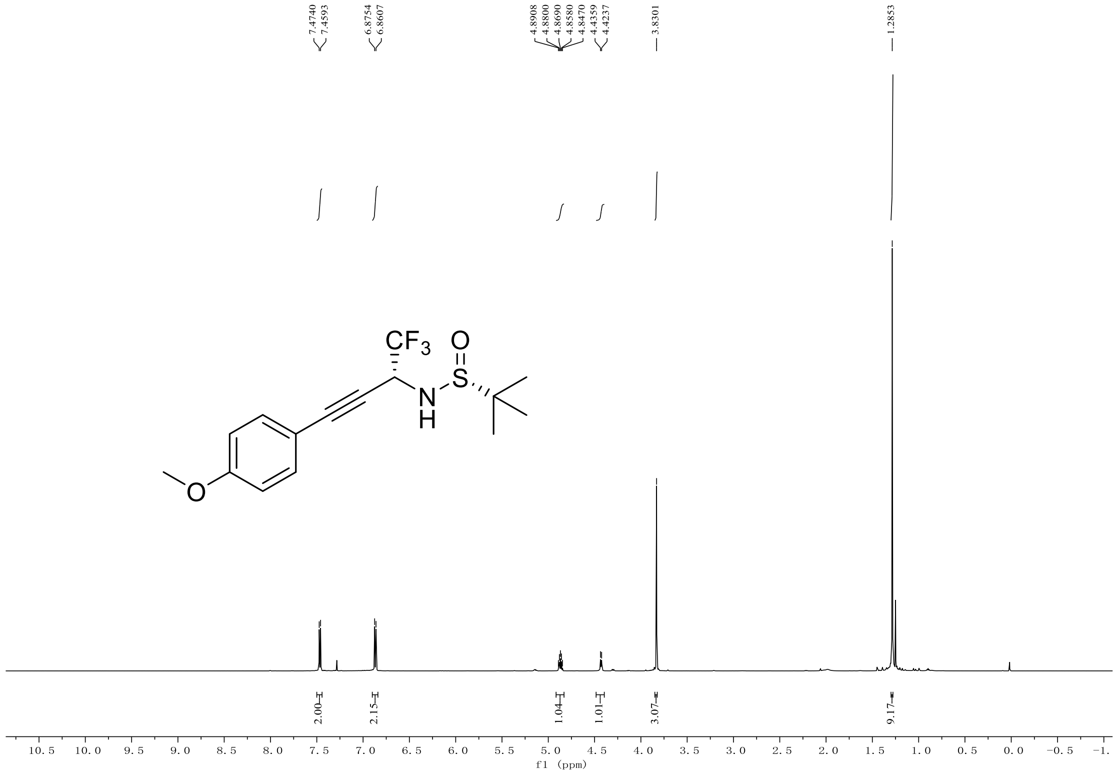

^{13}C NMR (100 MHz, $CDCl_3$) of (R_s, R)-**3a**:

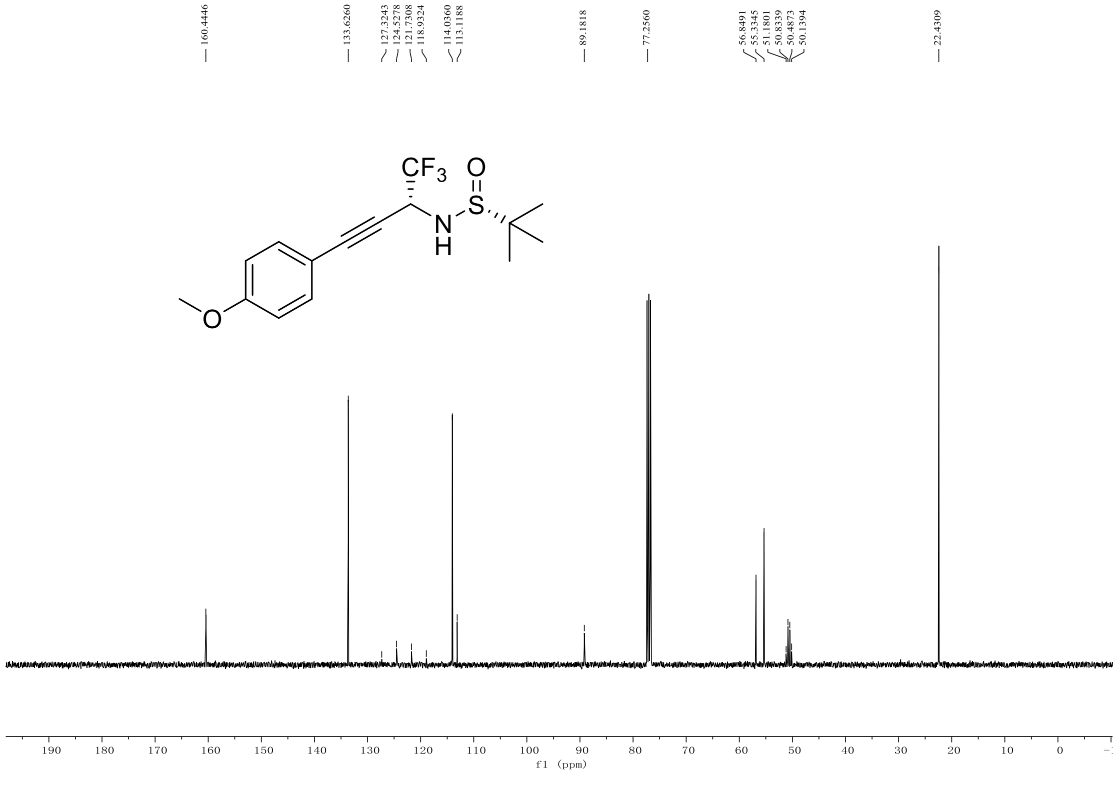

^{19}F NMR (376 MHz, $CDCl_3$) of (R_s, R)-**3a**:

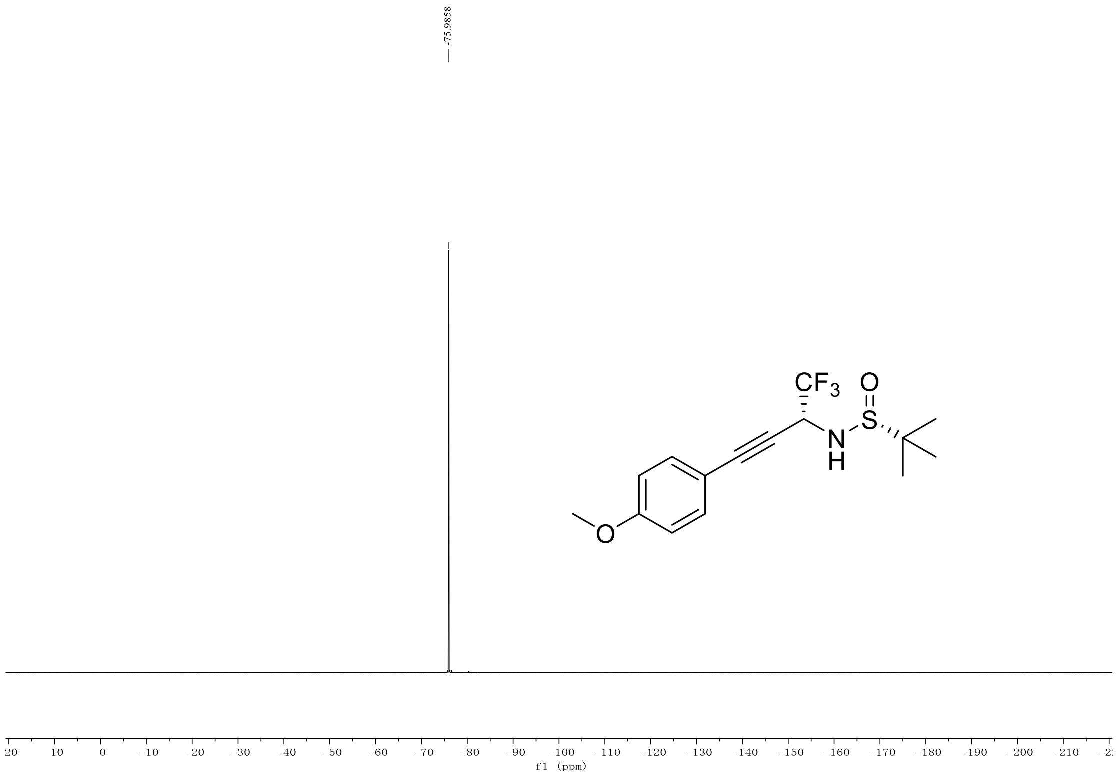

^{1}H NMR (600 MHz, $CDCl_3$) of (R_s, S)-**3a**:

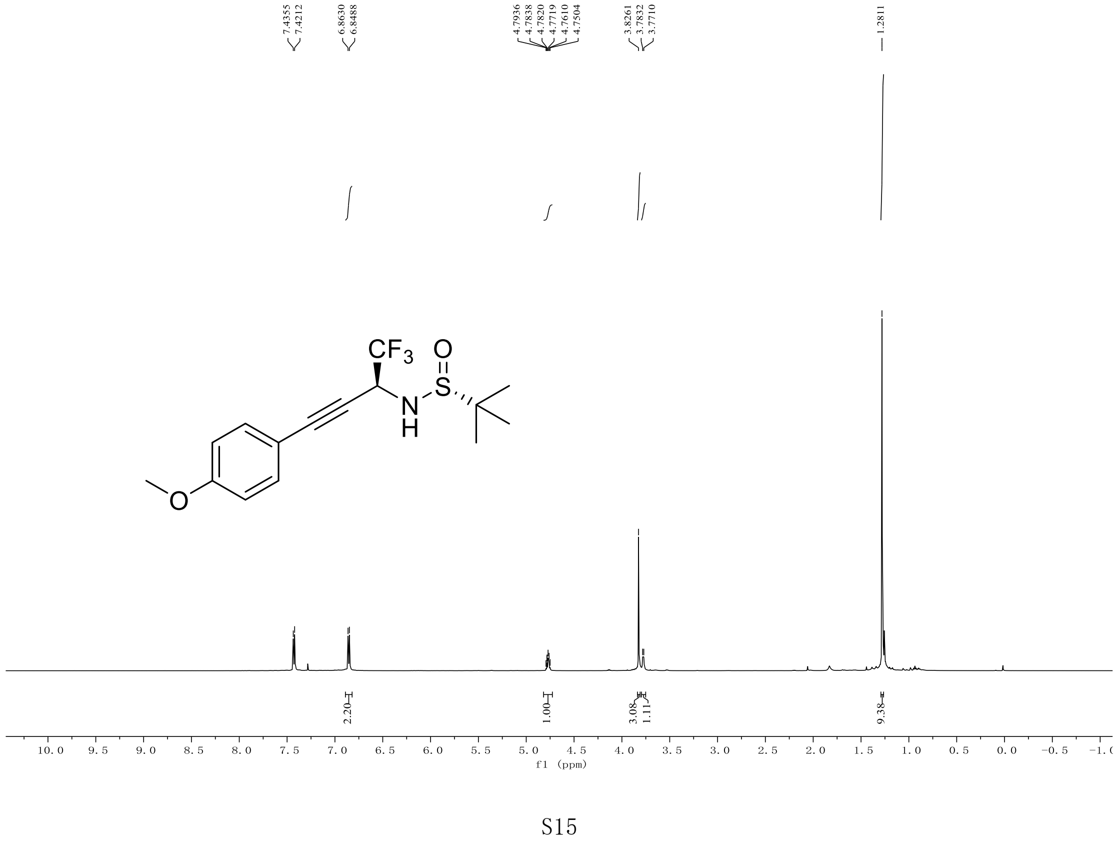

^{13}C NMR (100 MHz, CDCl$_3$) of (R_s, S)-**3a**:

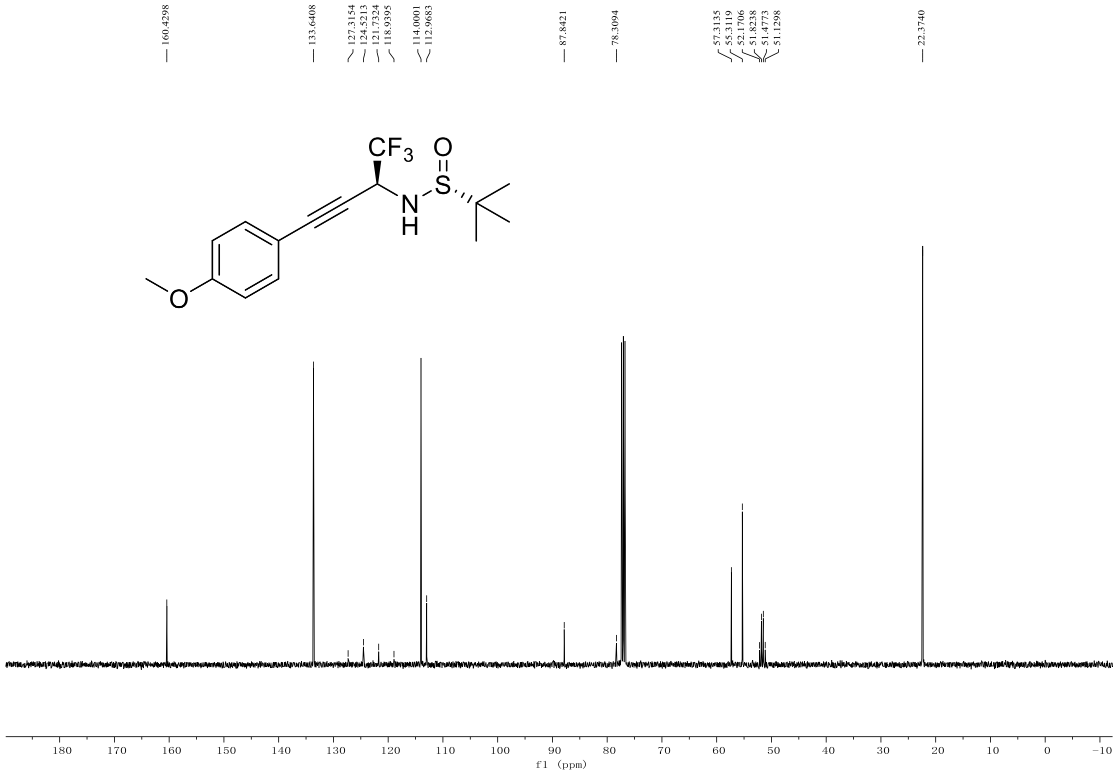

^{19}F NMR (376 MHz, CDCl$_3$) of (R_s, S)-**3a**:

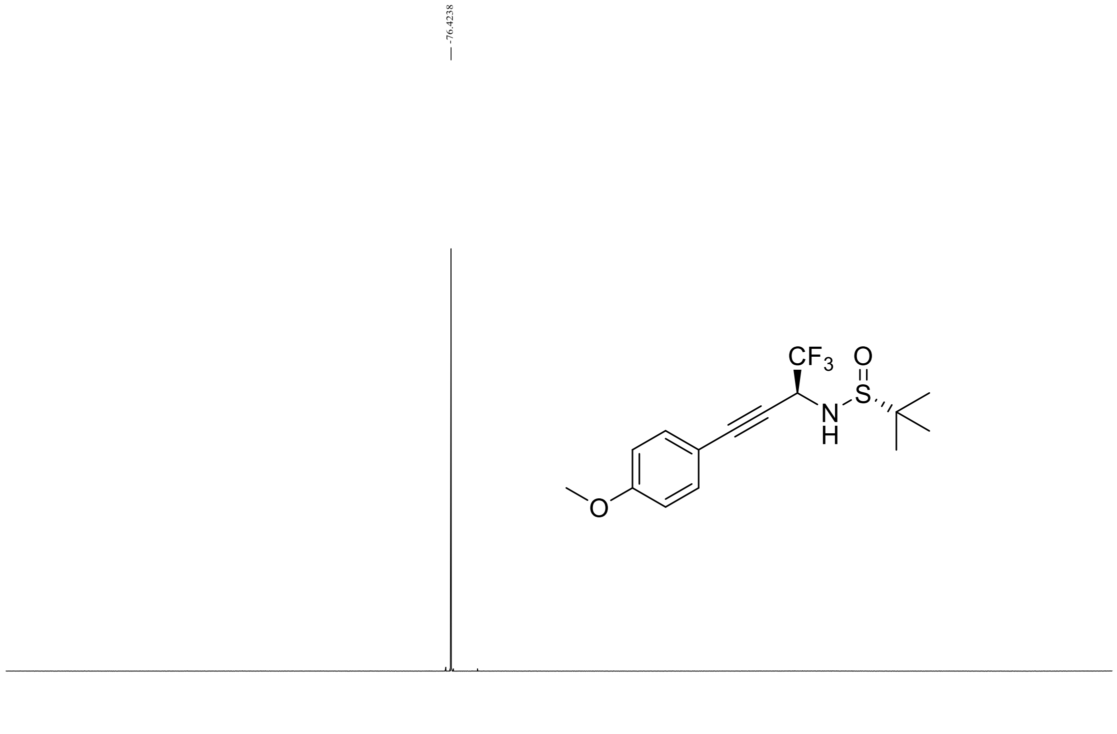

^{1}H NMR (600 MHz, $CDCl_3$) of (R_s, R)-**3b**:

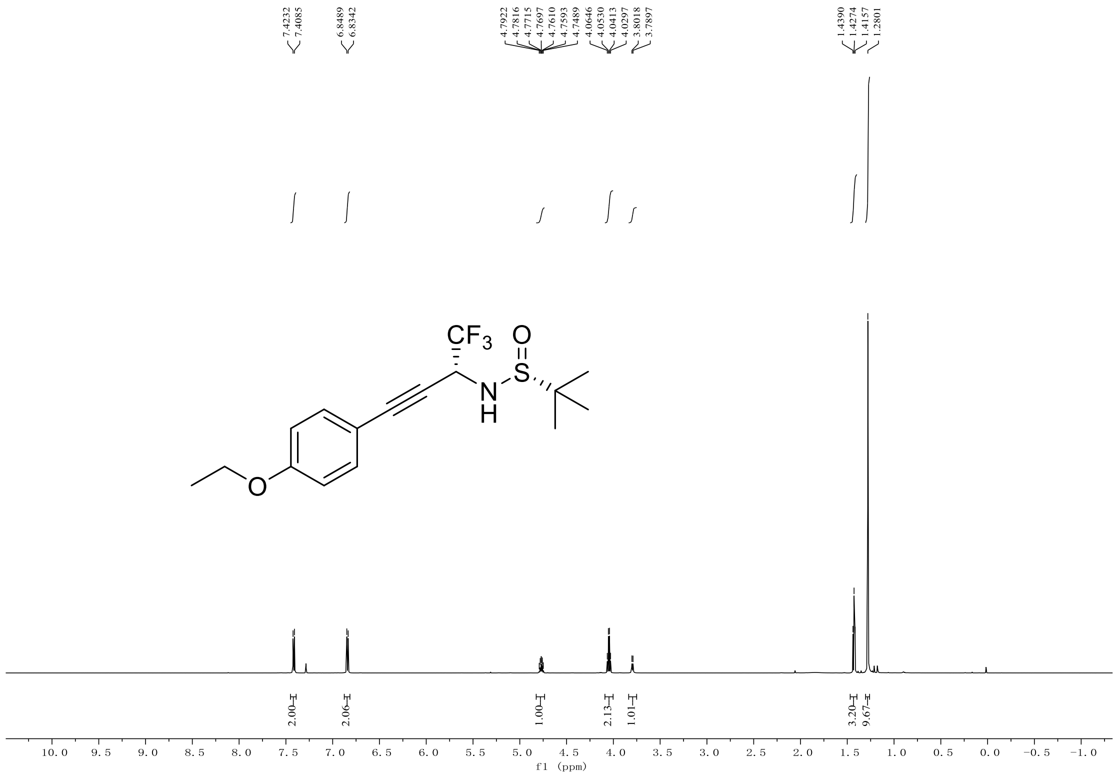

^{13}C NMR (150 MHz, $CDCl_3$) of (R_s, R)-**3b**:

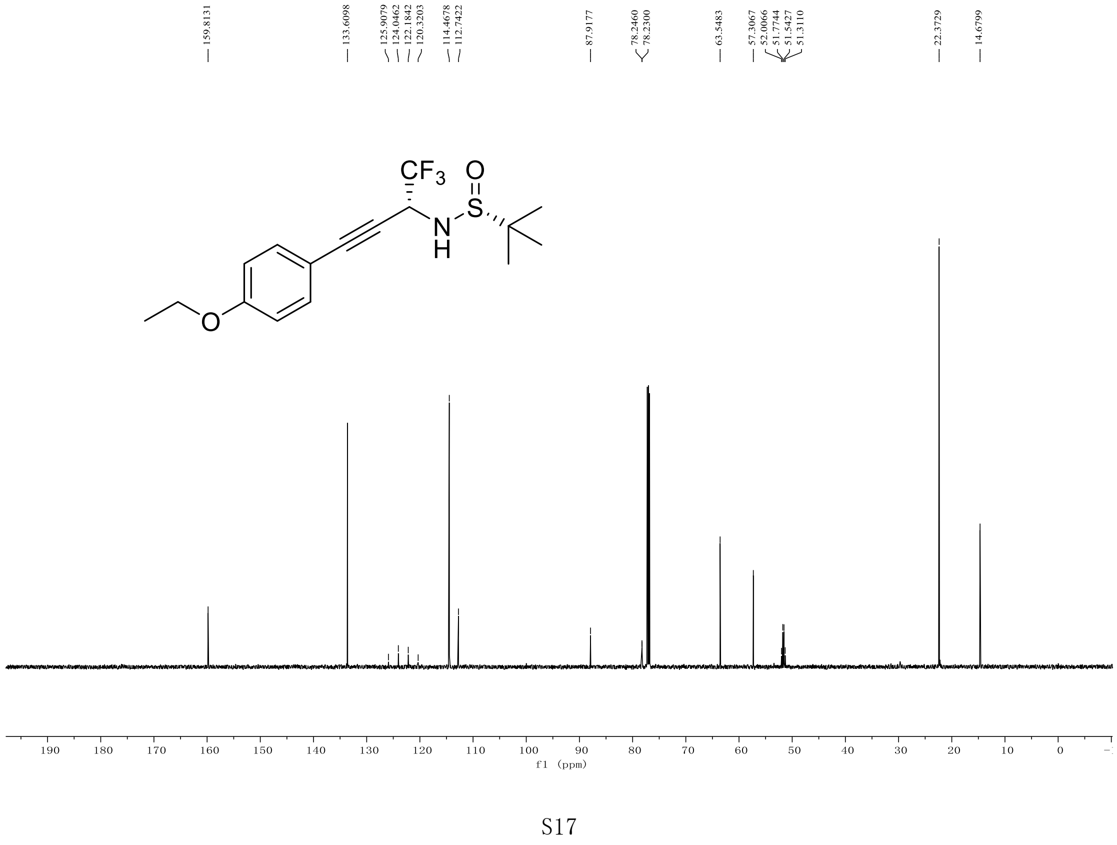

^{19}F NMR (565 MHz, $CDCl_3$) of (R_s, R)-**3b**:

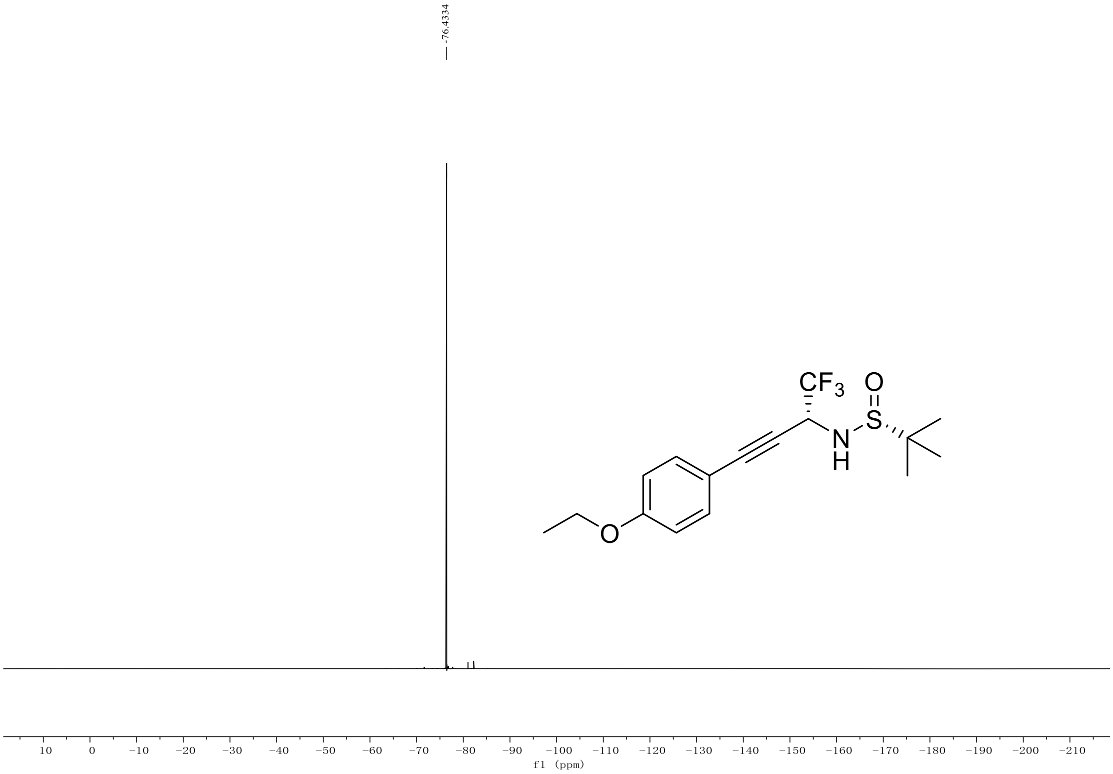

^{1}H NMR (600 MHz, $CDCl_3$) of (R_s, S)-**3b**:

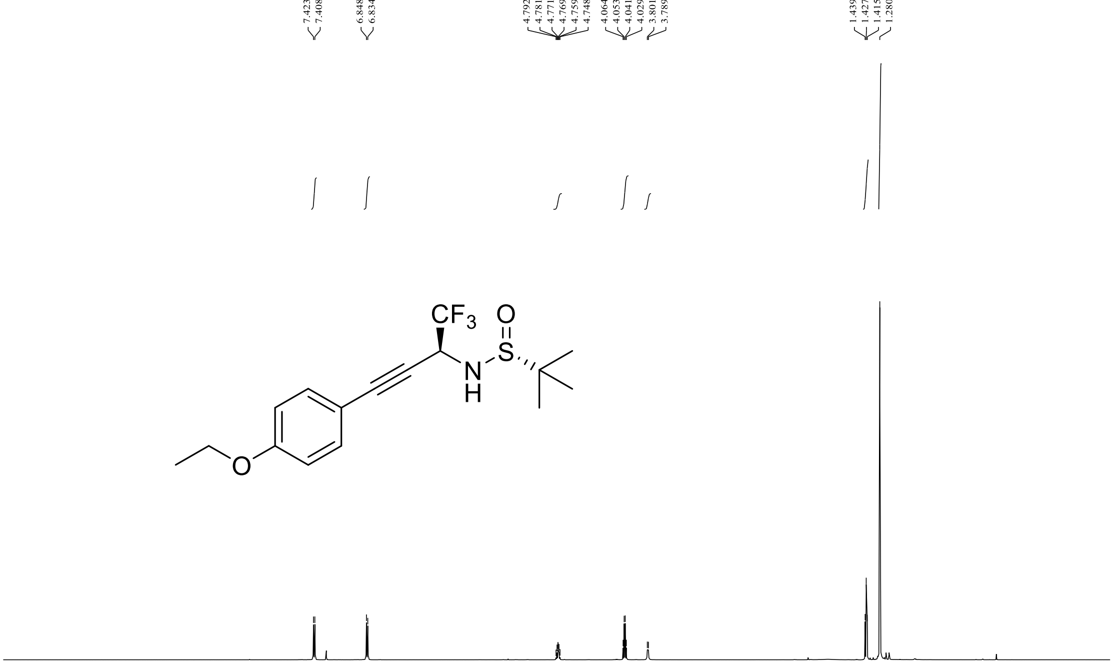

^{13}C NMR (150 MHz, $CDCl_3$) of (*R*s, *S*)-**3b**:

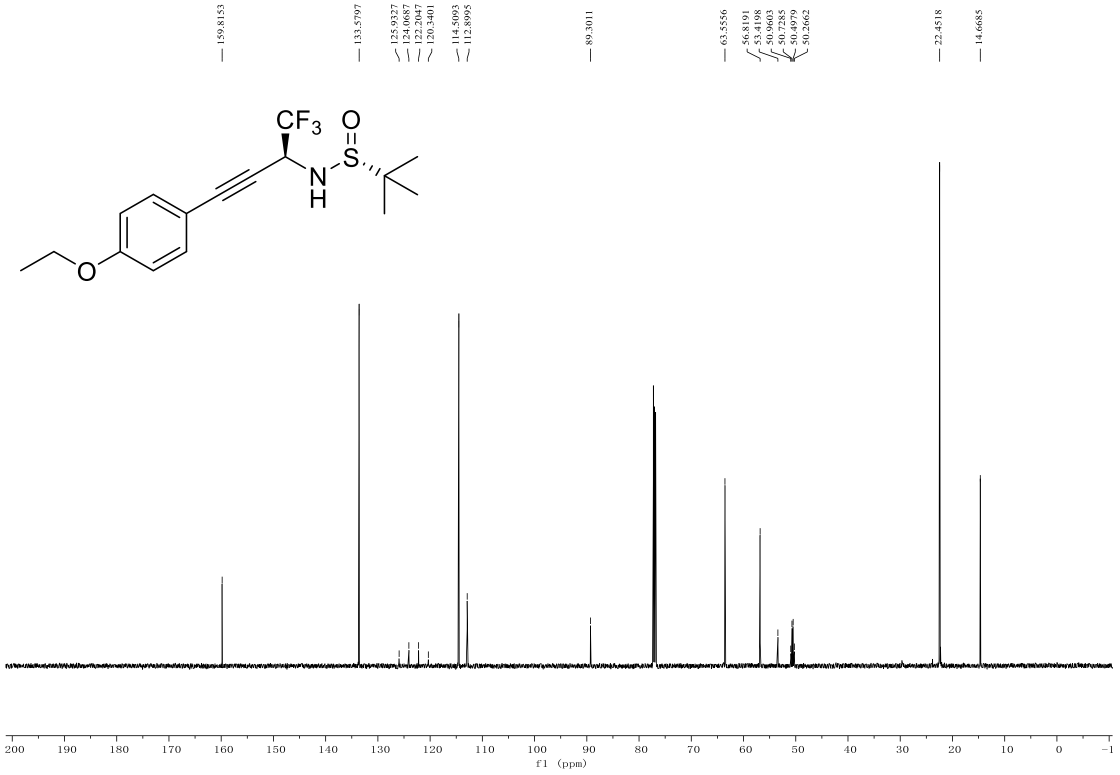

^{19}F NMR (565 MHz, $CDCl_3$) of (*R*s, *S*)-**3b**:

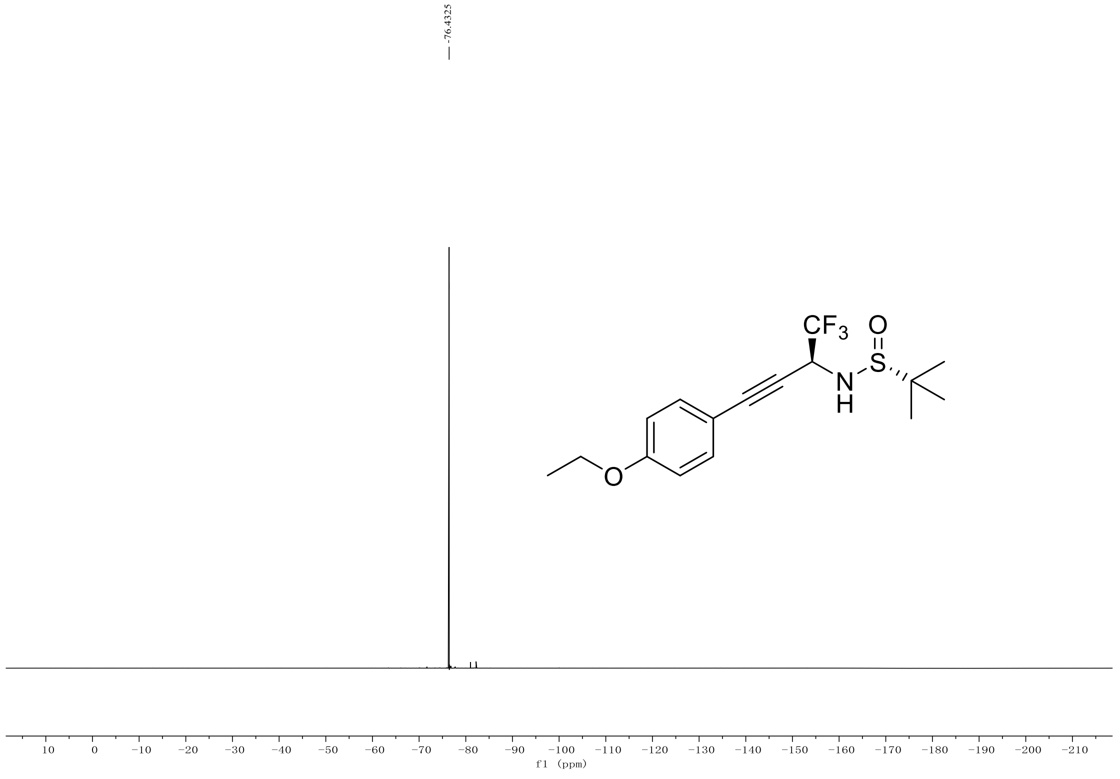

^{1}H NMR (400 MHz, $CDCl_3$) of (R_s, R)-**3c**:

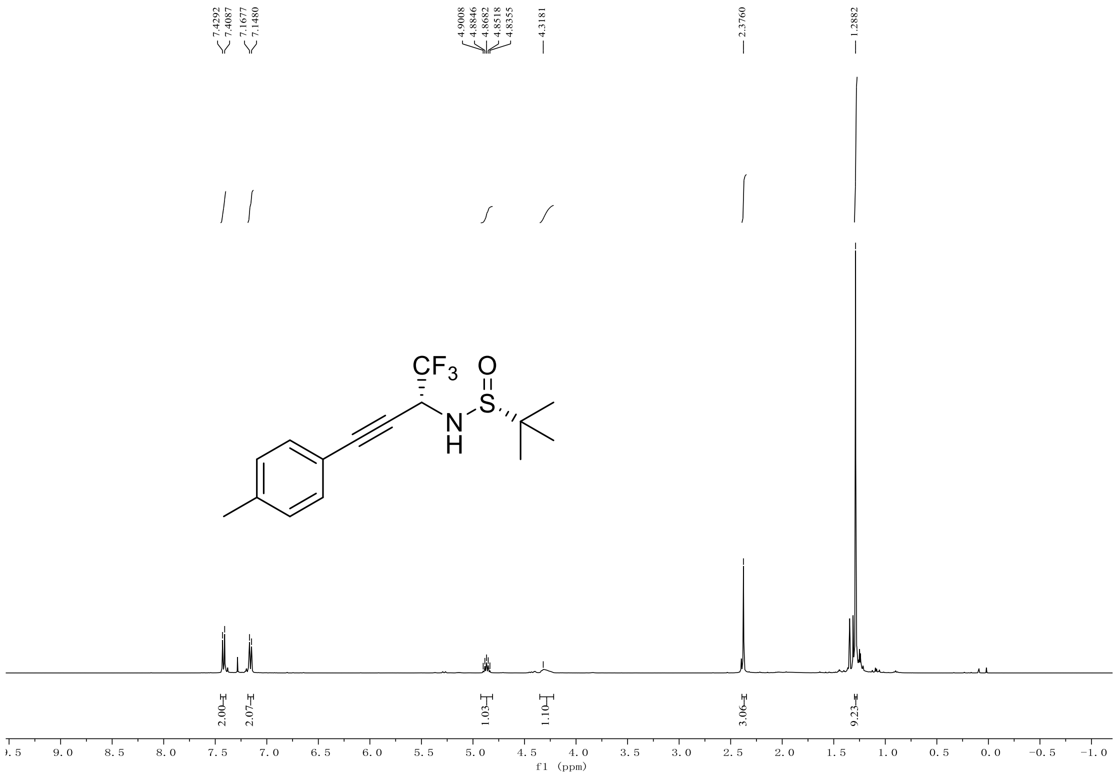

^{13}C NMR (100 MHz, $CDCl_3$) of (R_s, R)-**3c**:

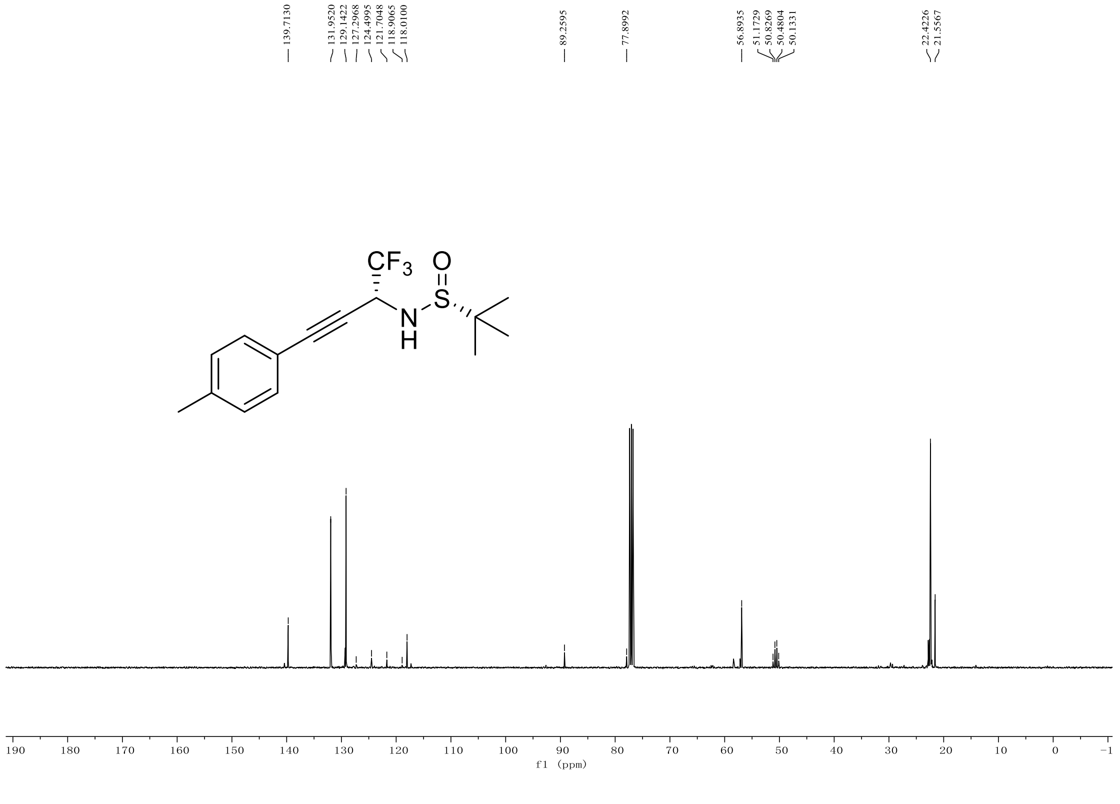

^{19}F NMR (565 MHz, $CDCl_3$) of (R_s, R)-**3c**:

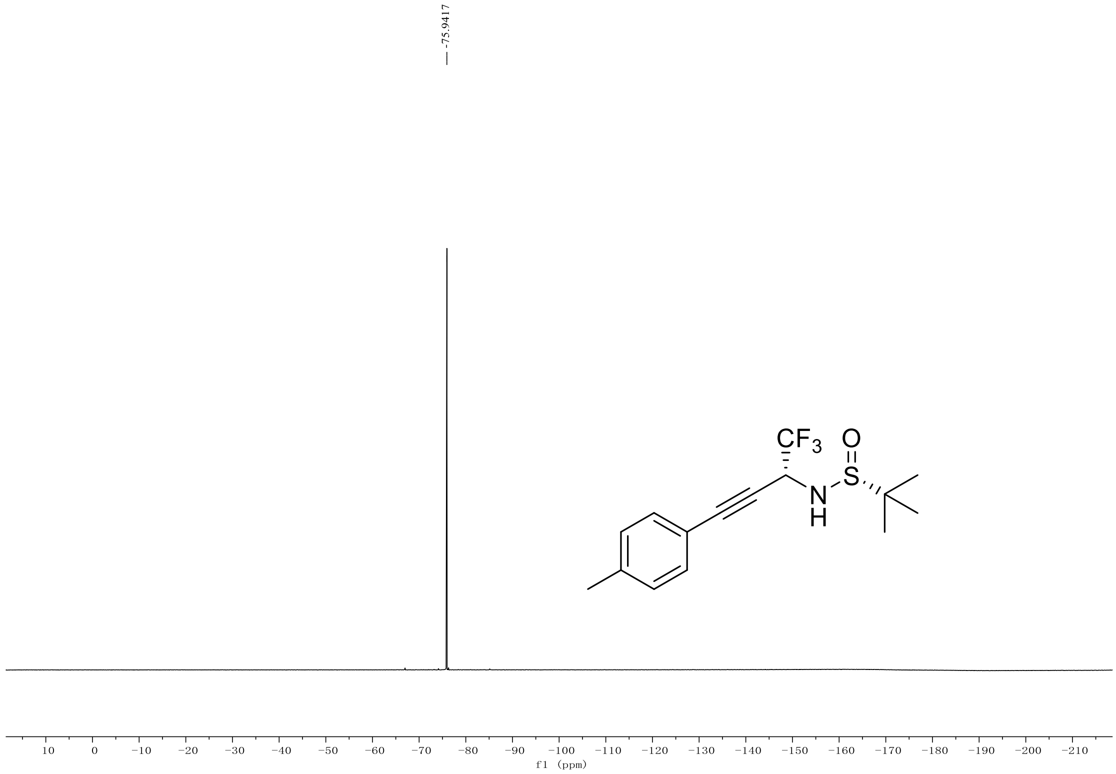

^{1}H NMR (400 MHz, $CDCl_3$) of (R_s, S)-**3c**:

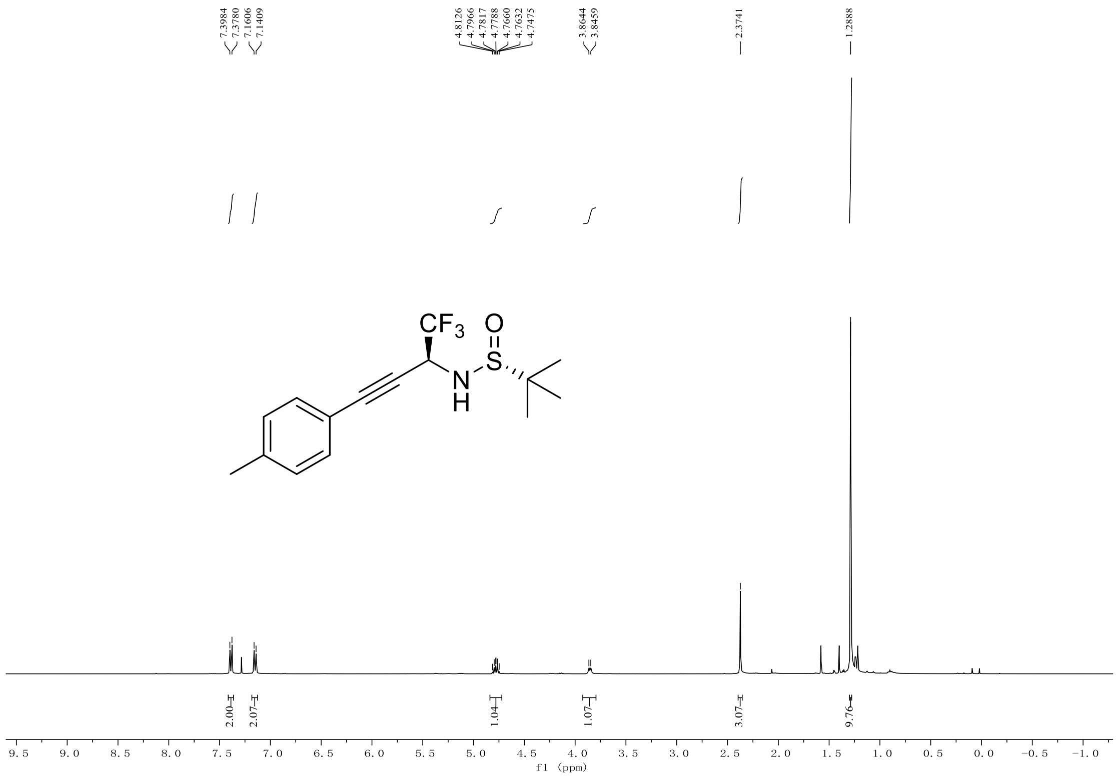

^{13}C NMR (100 MHz, $CDCl_3$) of (R_s, S)-**3c**:

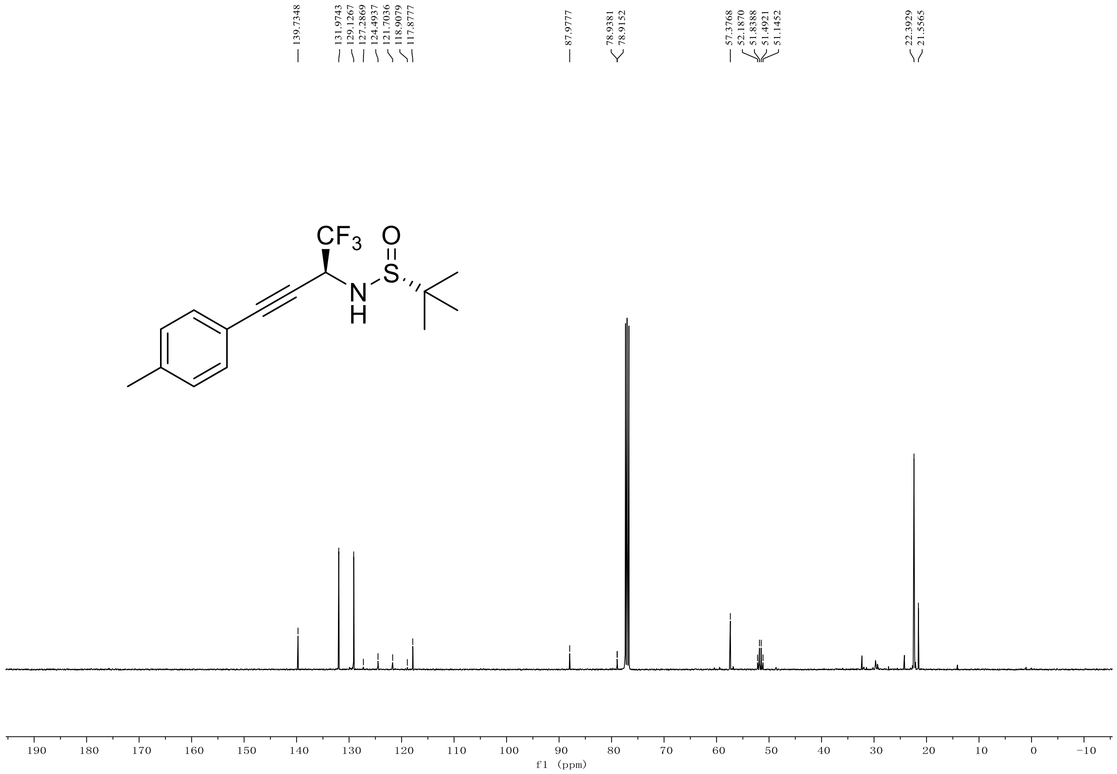

^{19}F NMR (565 MHz, $CDCl_3$) of (R_s, S)-**3c**:

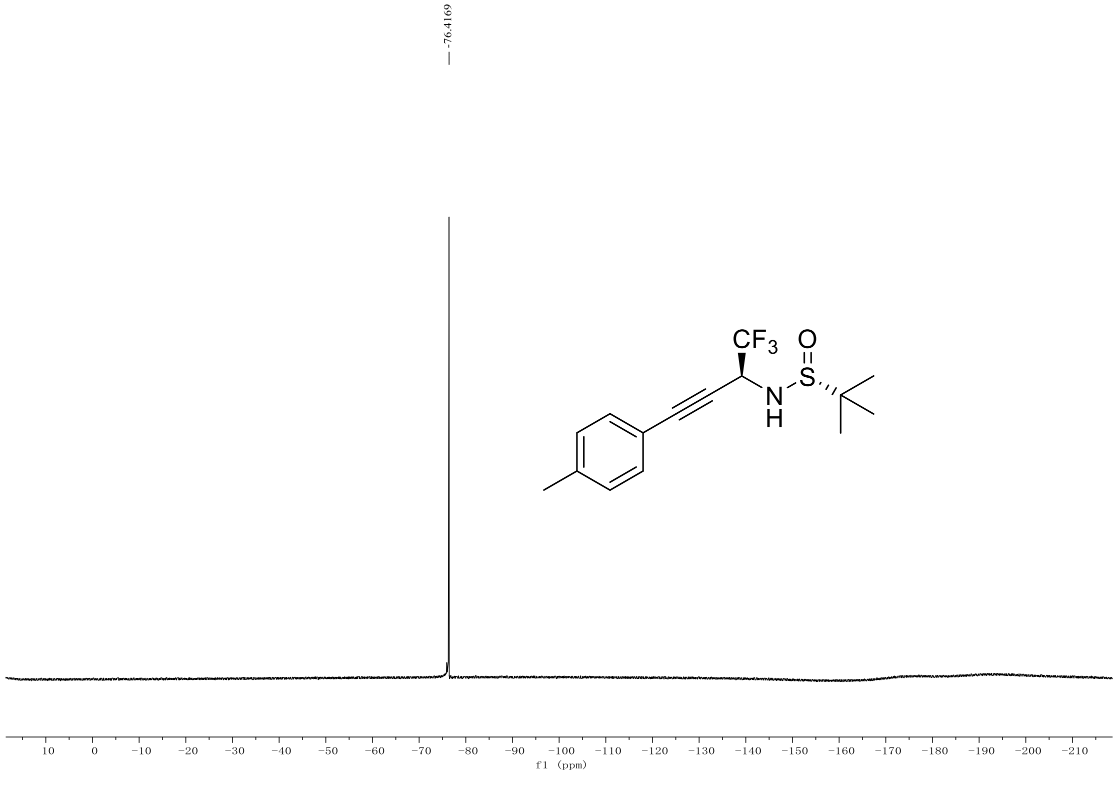

^{1}H NMR (600 MHz, $CDCl_3$) of (R_s, *R*)-**3d**:

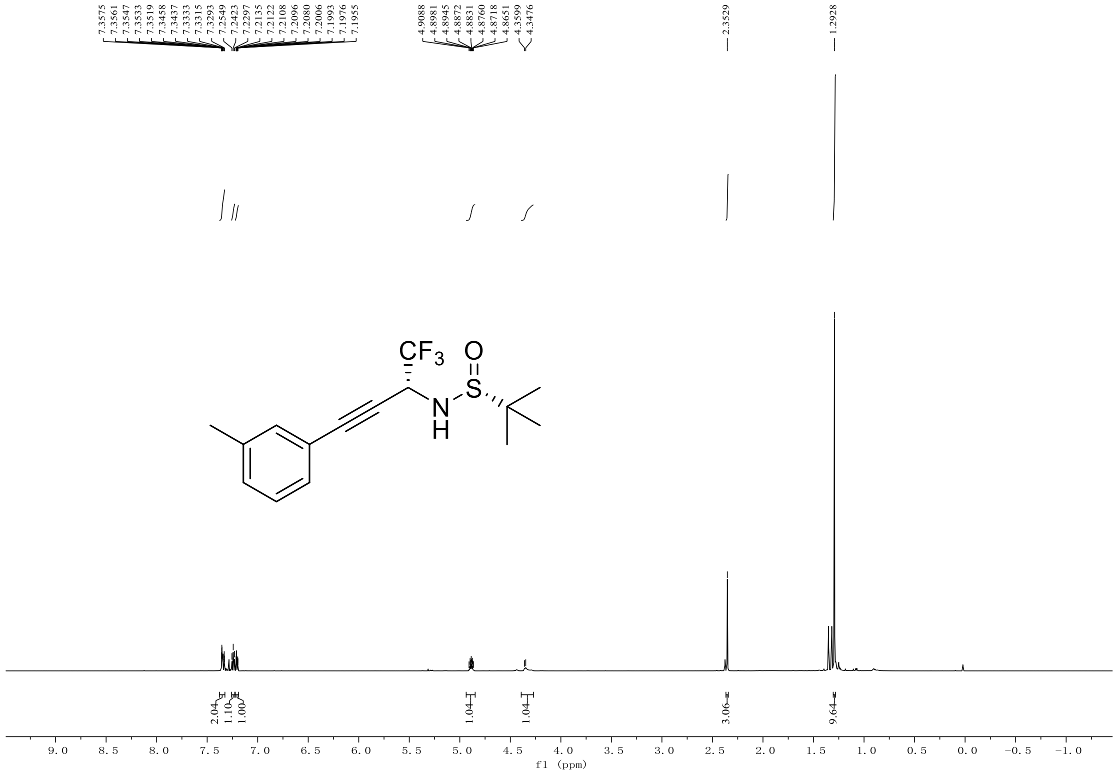

^{13}C NMR (150 MHz, $CDCl_3$) of (R_s, *R*)-**3d**:

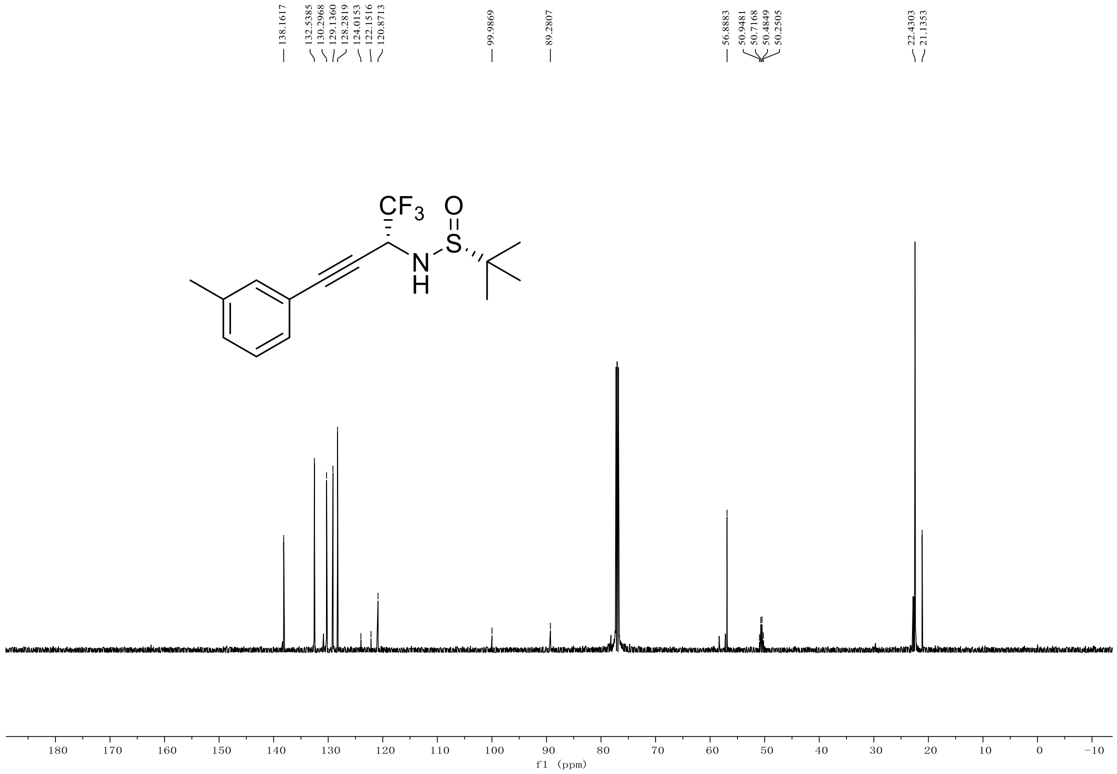

^{19}F NMR (565 MHz, $CDCl_3$) of (R_s, R)-**3d**:

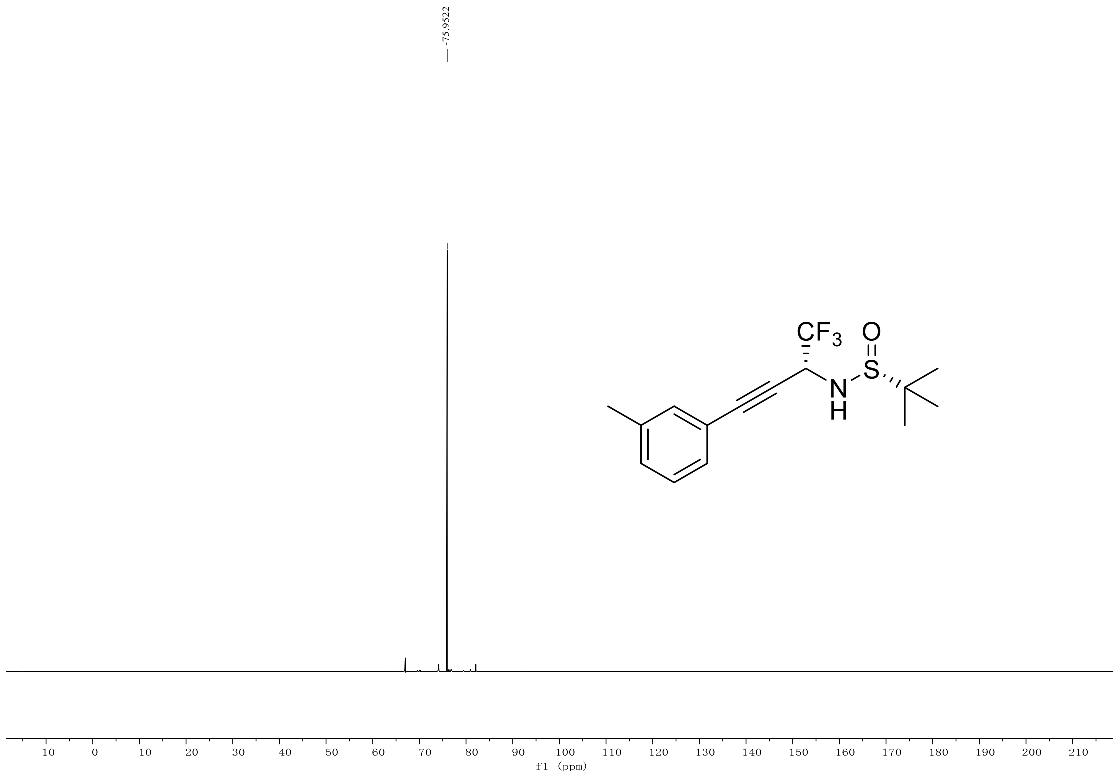

^{1}H NMR (600 MHz, $CDCl_3$) of (R_s, S)-**3d**:

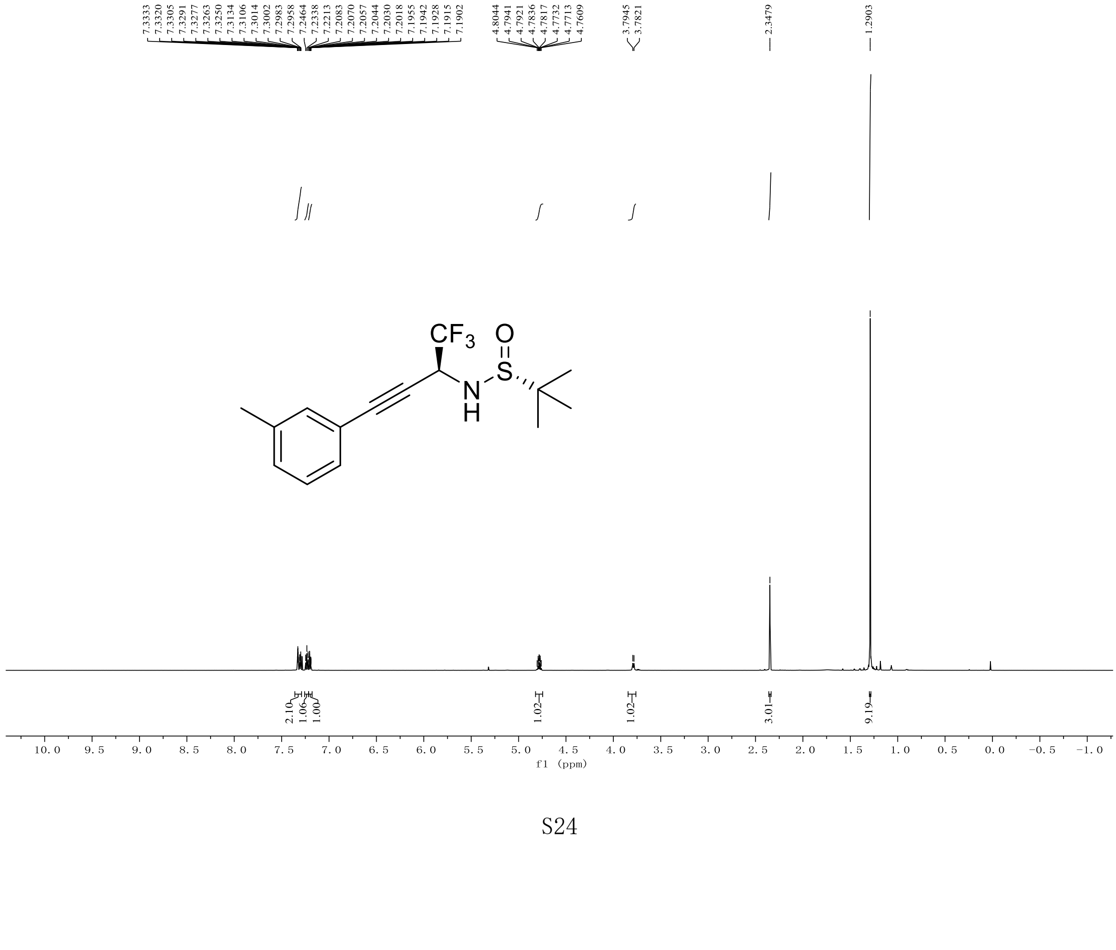

^{13}C NMR (150 MHz, $CDCl_3$) of (R_s, S)-**3d**:

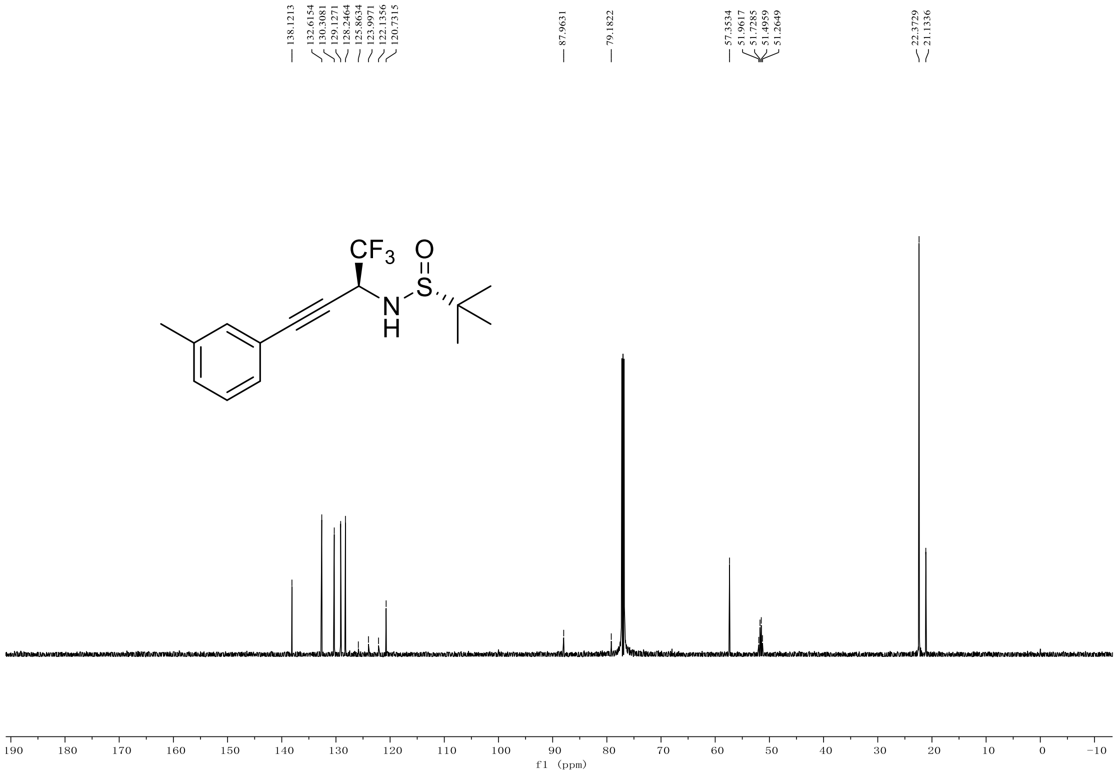

^{19}F NMR (565 MHz, $CDCl_3$) of (R_s, S)-**3d**:

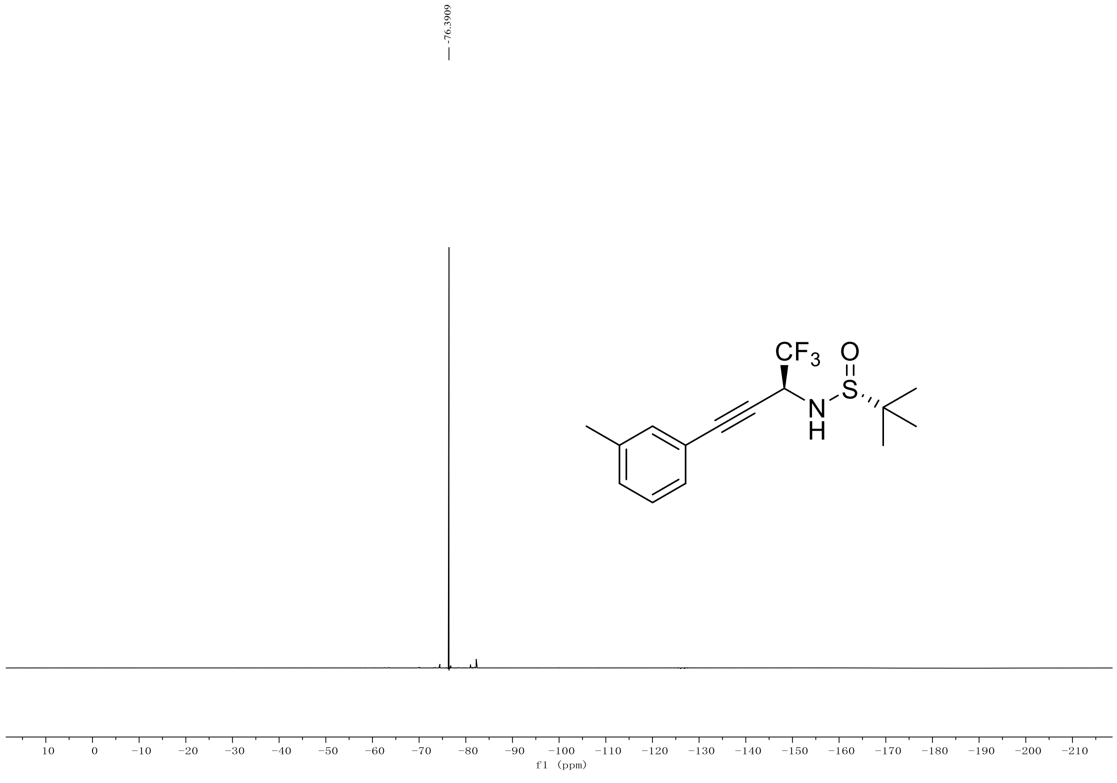

^{1}H NMR (400 MHz, $CDCl_3$) of (R_s, R)-**3e**:

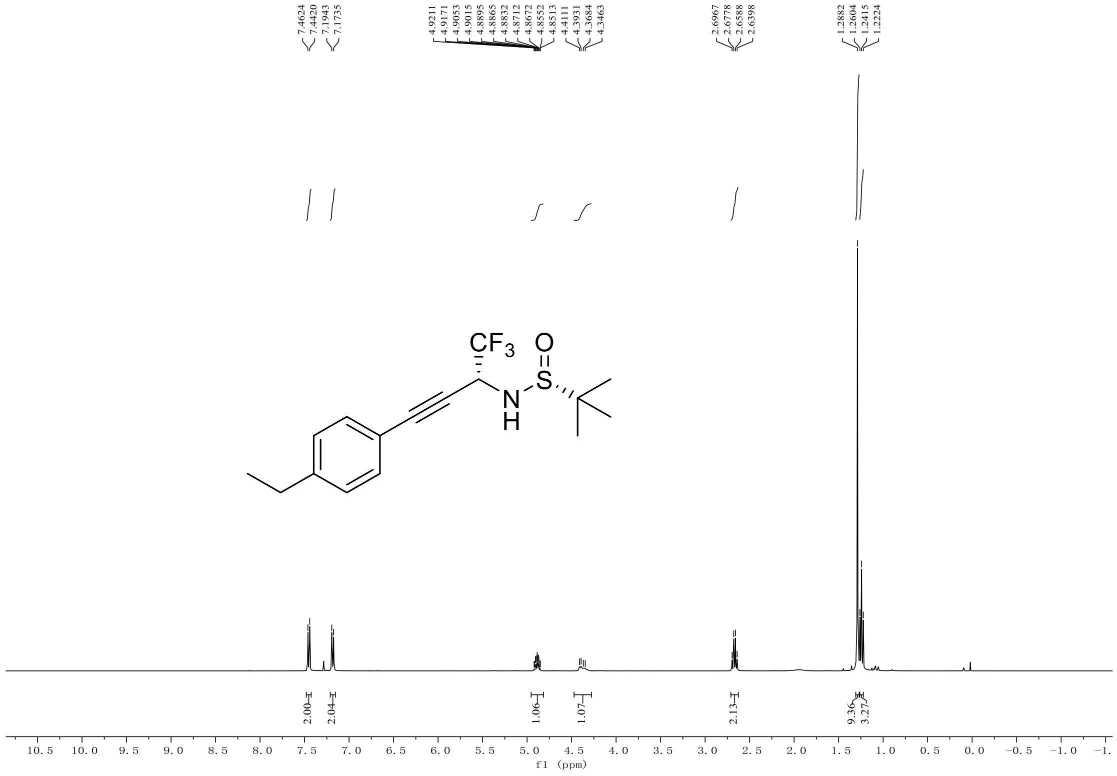

^{13}C NMR (100 MHz, $CDCl_3$) of (R_s, R)-**3e**:

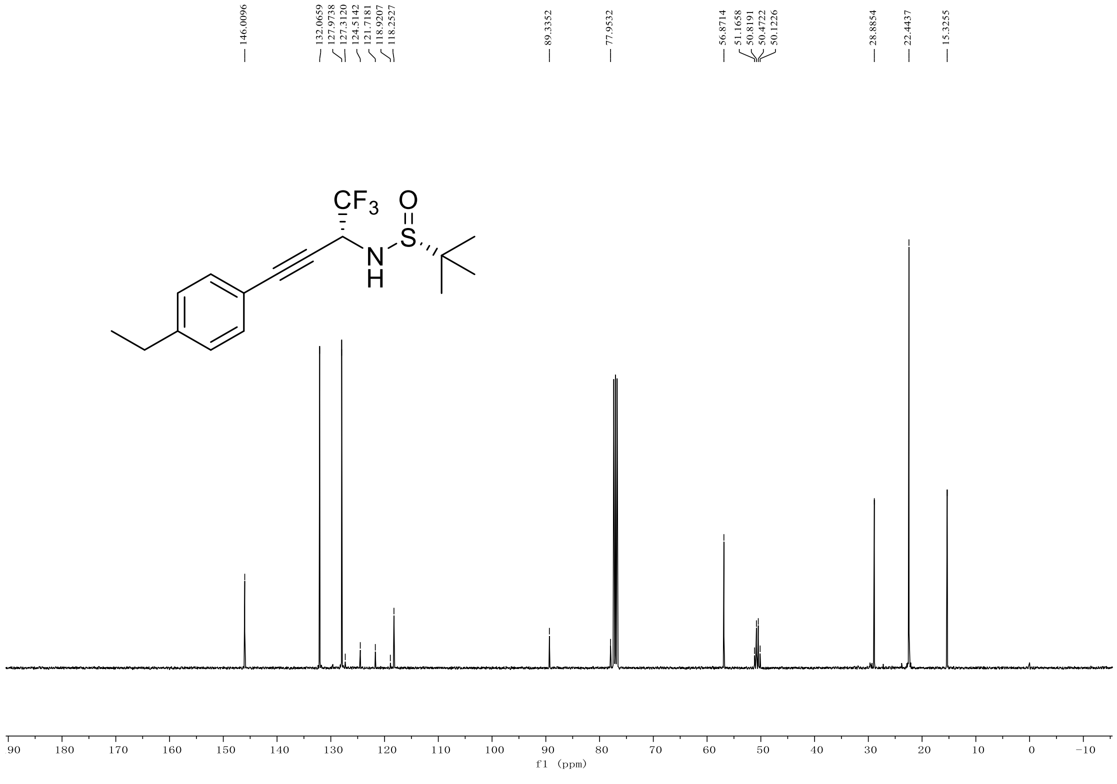

^{19}F NMR (376 MHz, $CDCl_3$) of (R_s, R)-**3e**:

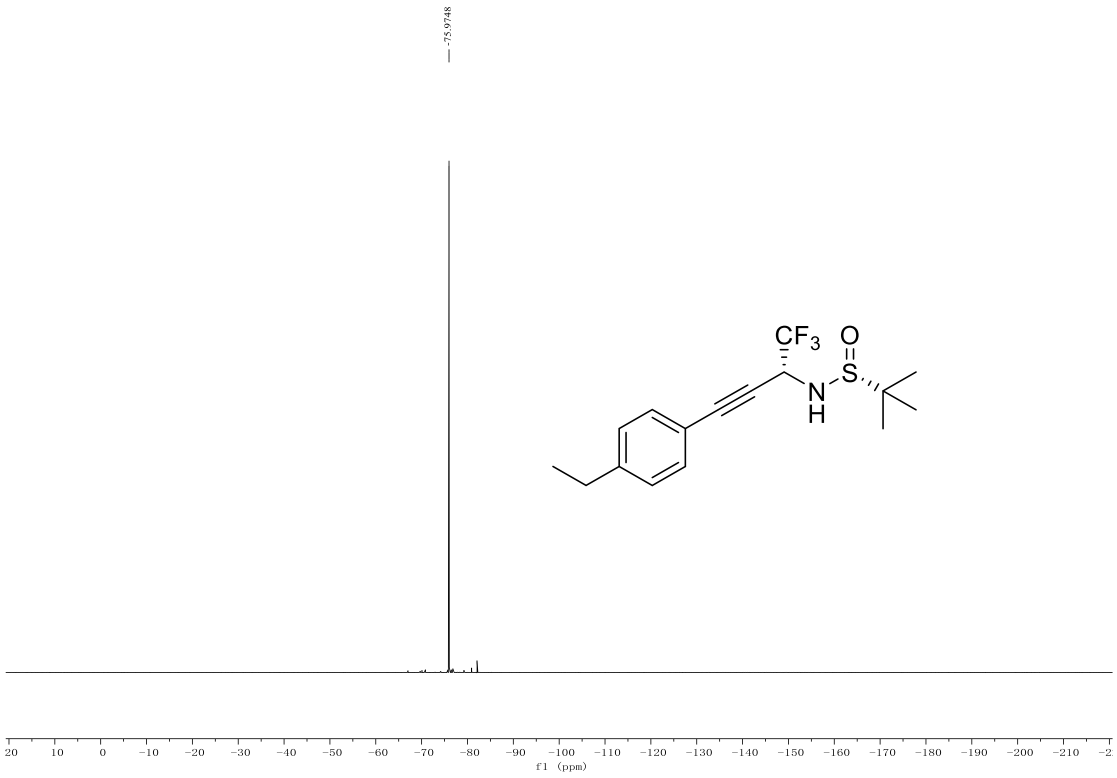

^{1}H NMR (400 MHz, $CDCl_3$) of (R_s, S)-**3e**:

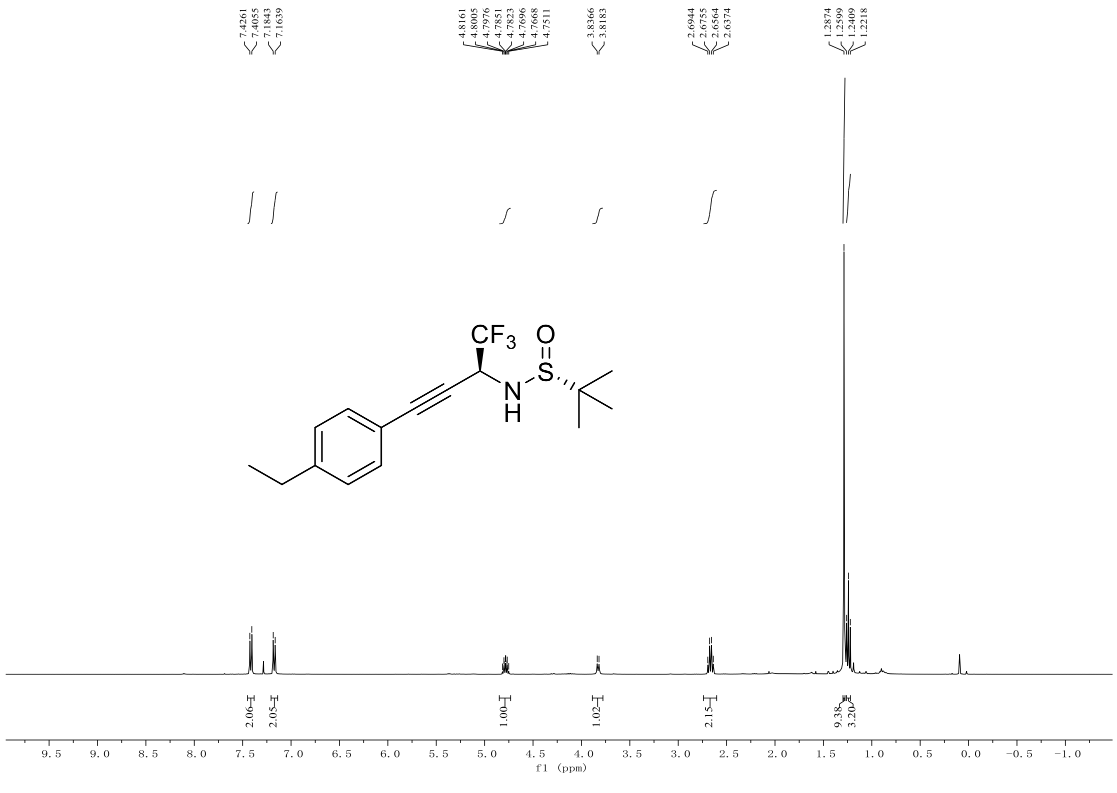

^{13}C NMR (100 MHz, $CDCl_3$) of (R_s, S)-**3e**:

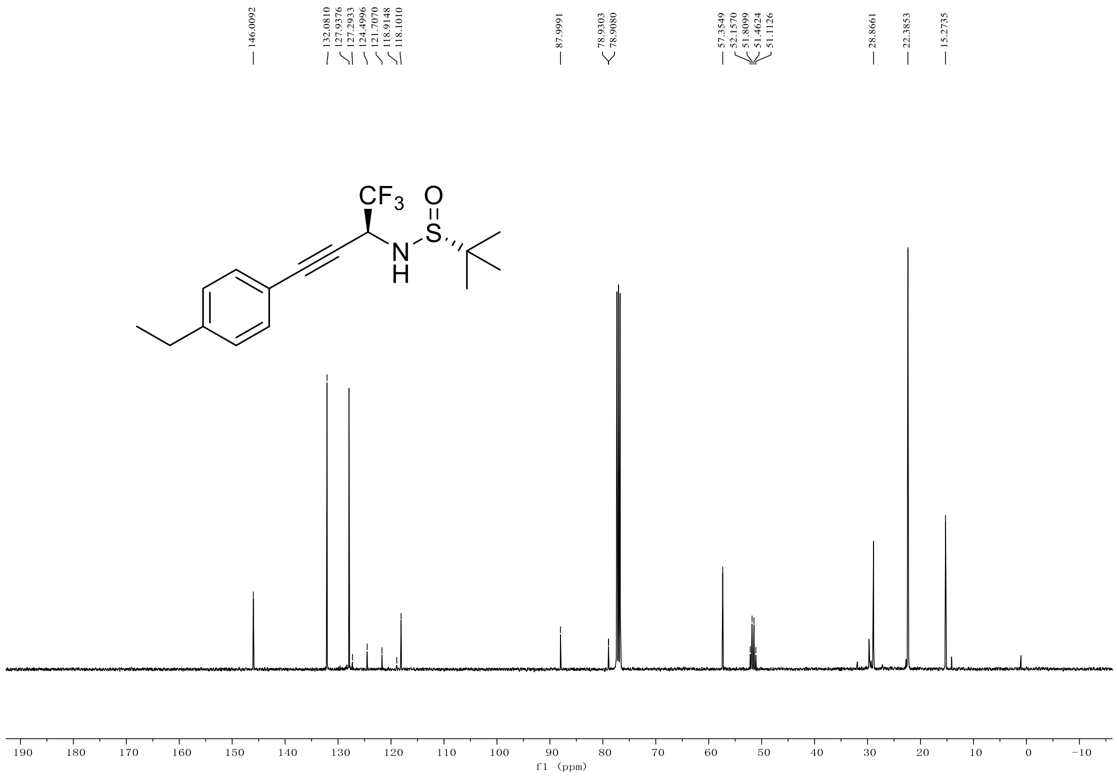

^{19}F NMR (376 MHz, $CDCl_3$) of (R_s, S)-**3e**:

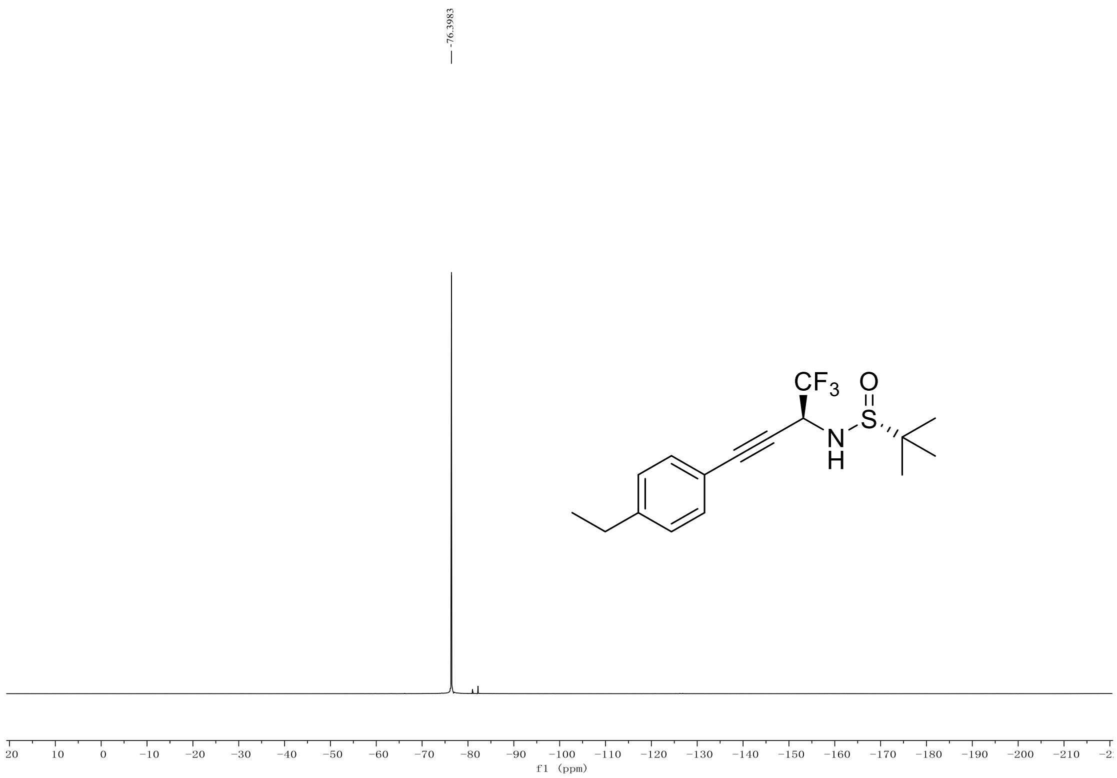

^{1}H NMR (400 MHz, $CDCl_3$) of (R_s, R)-**3f**:

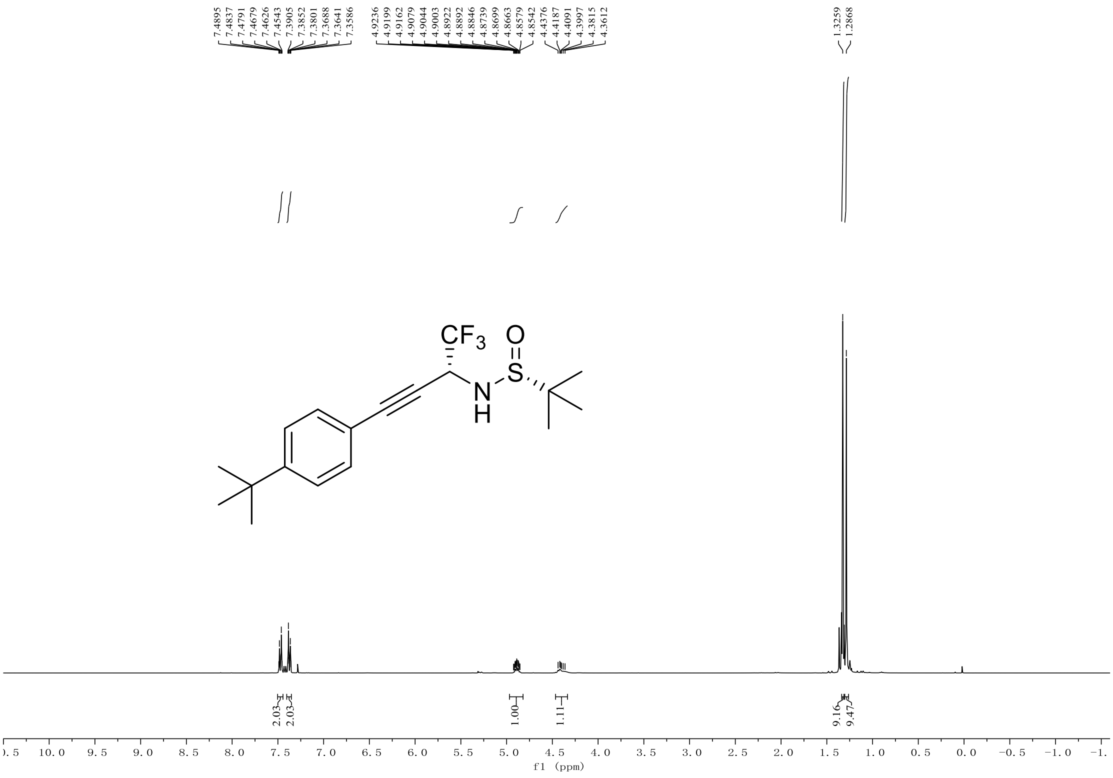

^{13}C NMR (150 MHz, $CDCl_3$) of (R_s, R)-**3f**:

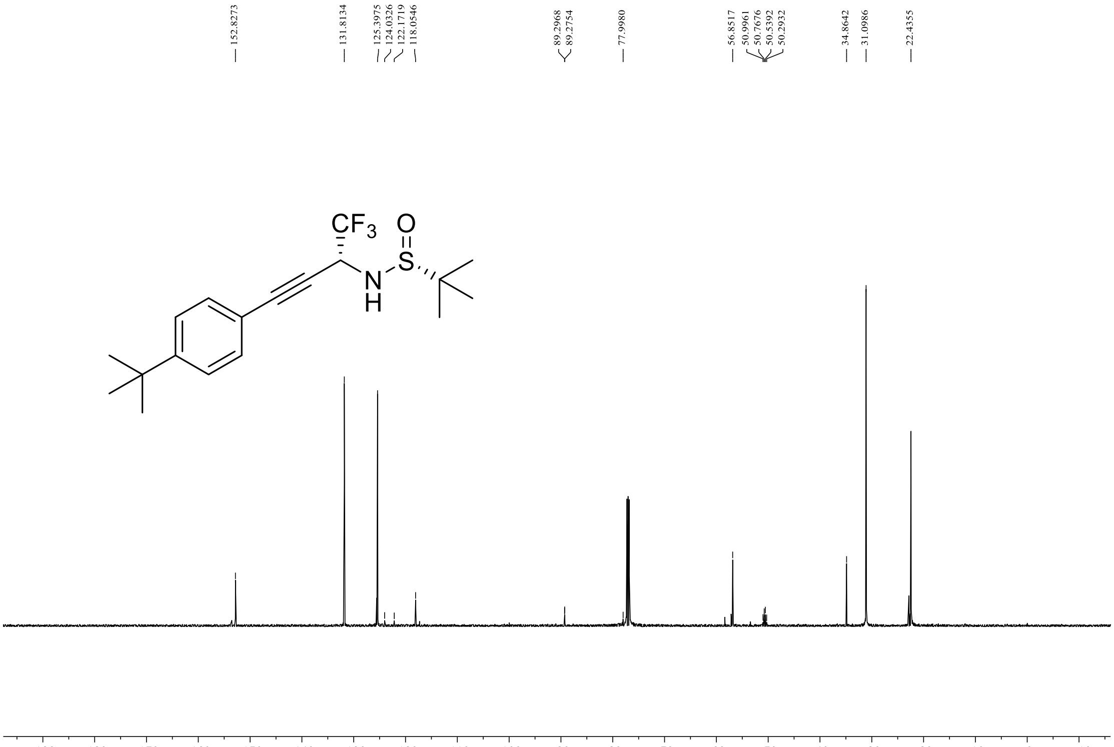

^{19}F NMR (565 MHz, CDCl$_3$) of (R_s, R)-**3f**:

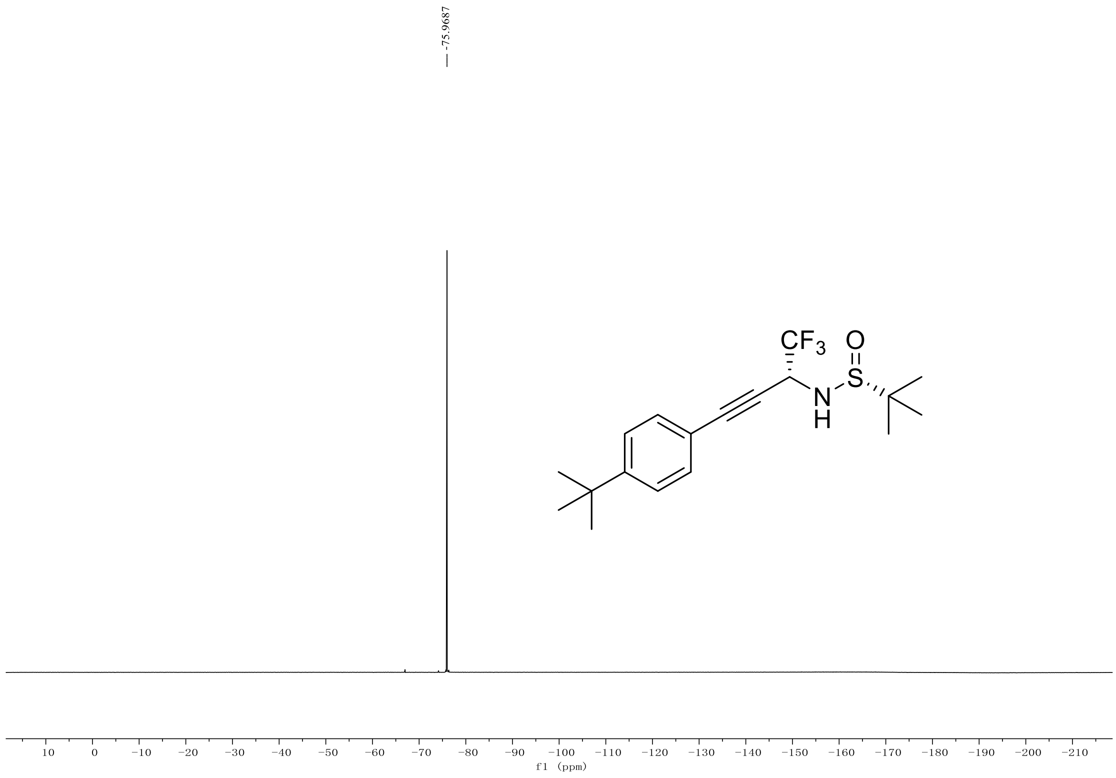

^{1}H NMR (400 MHz, CDCl$_3$) of (R_s, S)-**3f**:

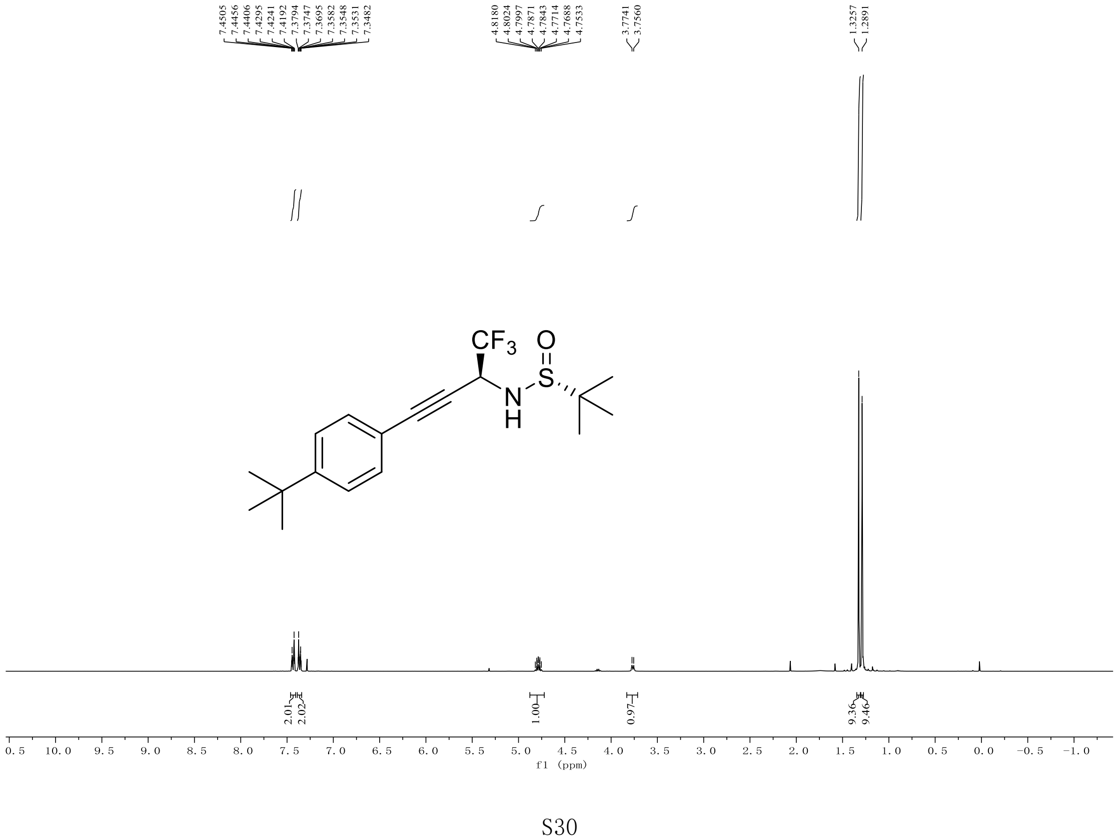

^{13}C NMR (150 MHz, $CDCl_3$) of (R_s, S)-**3f**:

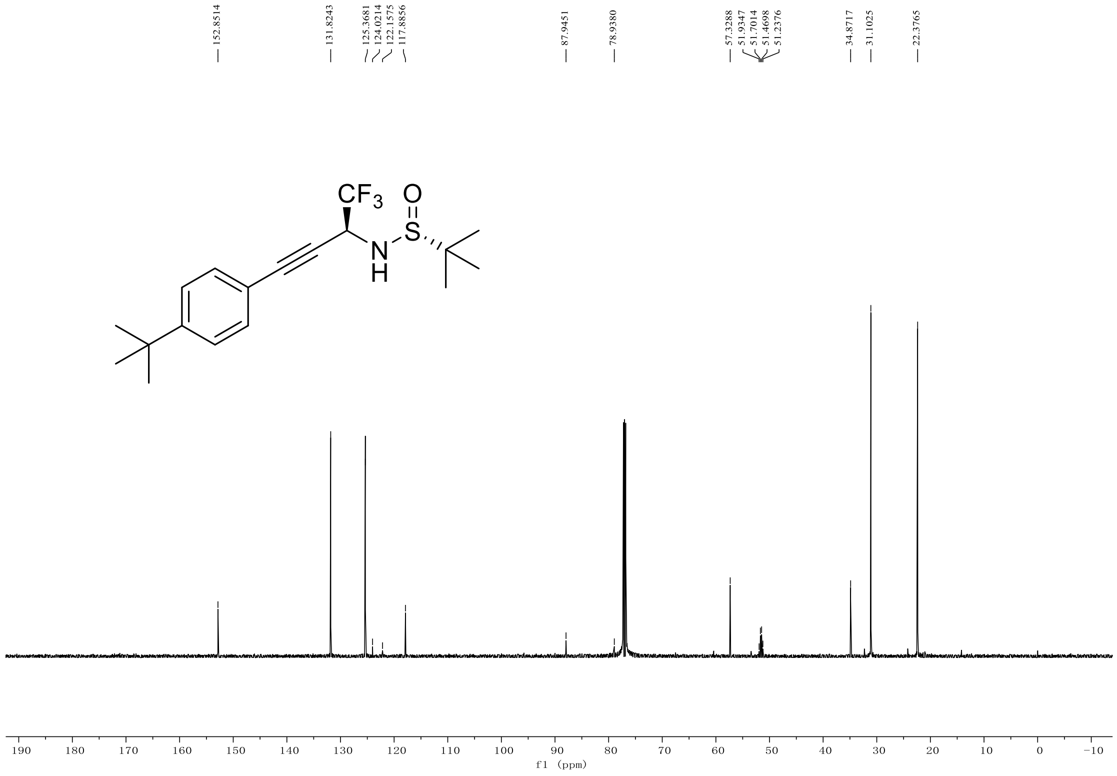

^{19}F NMR (565 MHz, $CDCl_3$) of (R_s, S)-**3f**:

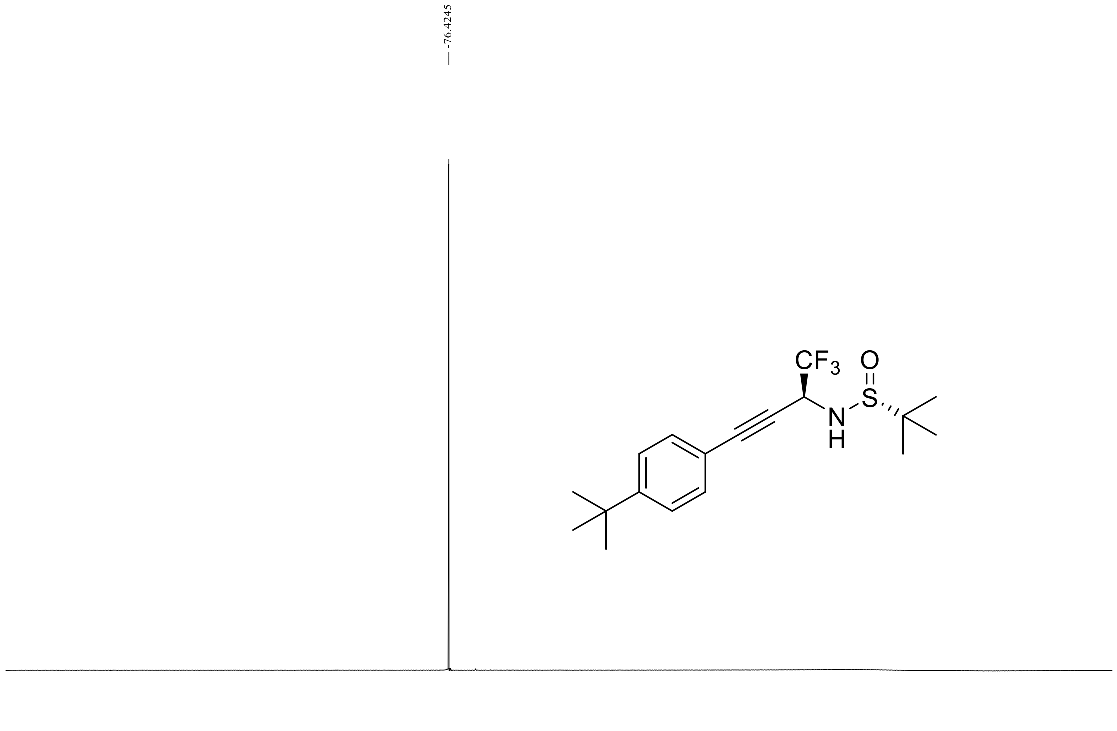

^{1}H NMR (400 MHz, $CDCl_3$) of (R_s, R)-**3g**:

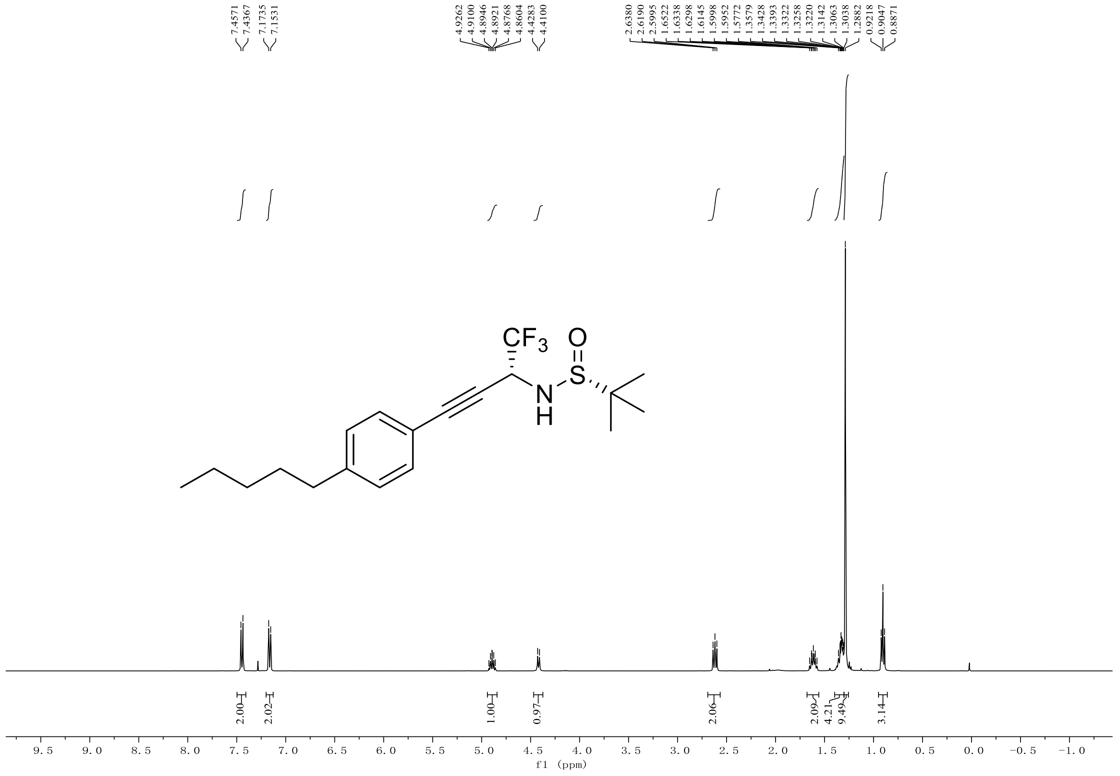

^{13}C NMR (150 MHz, $CDCl_3$) of (R_s, R)-**3g**:

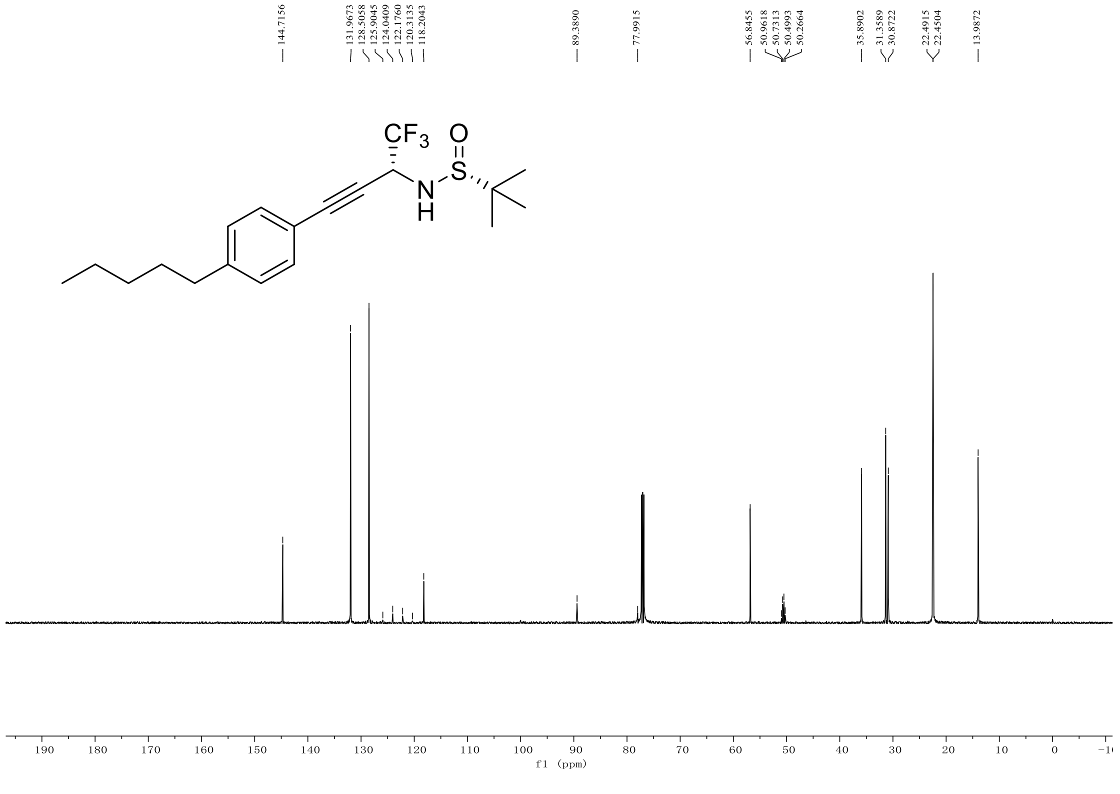

^{19}F NMR (376 MHz, $CDCl_3$) of (*R*$_s$, *R*)-**3g**:

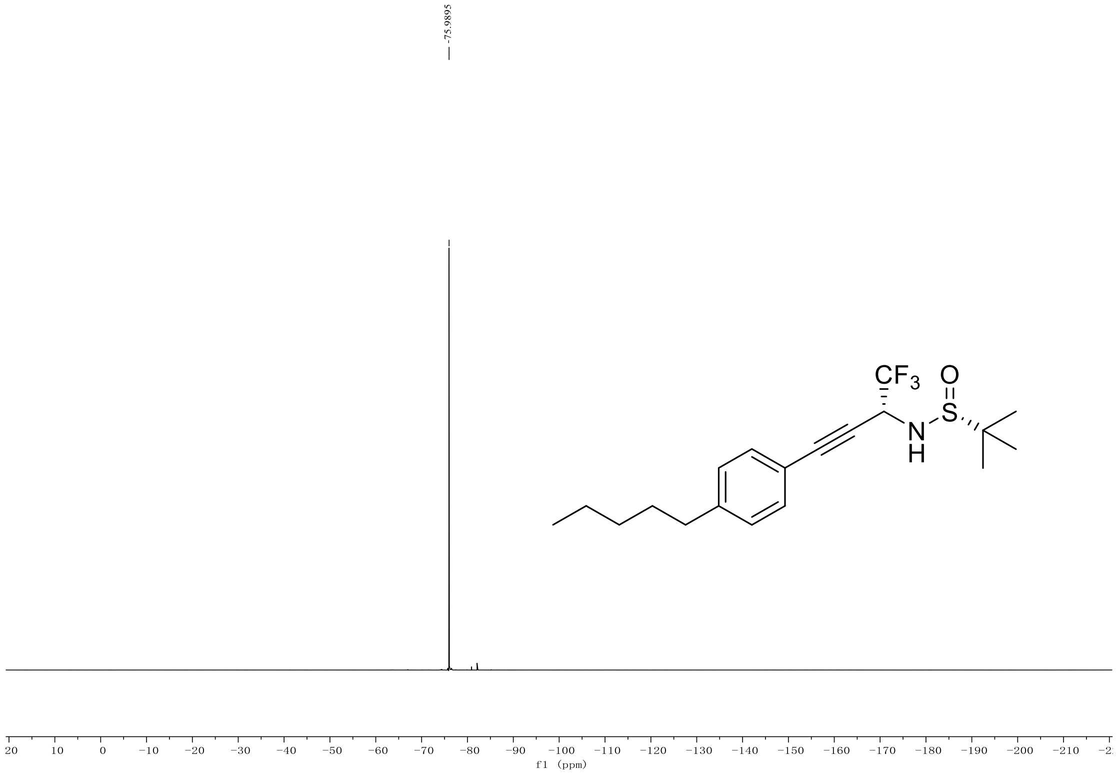

^{1}H NMR (400 MHz, $CDCl_3$) of (*R*$_s$, *S*)-**3g**:

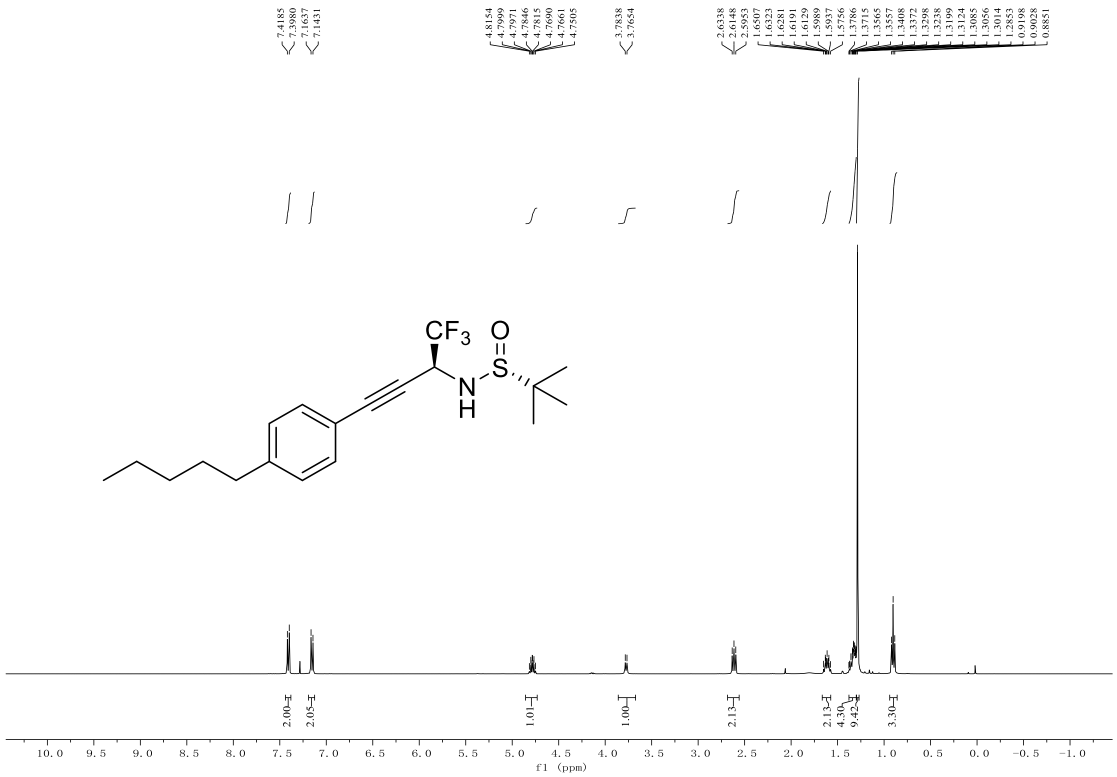

^{13}C NMR (150 MHz, $CDCl_3$) of (R_s, S)-**3g**:

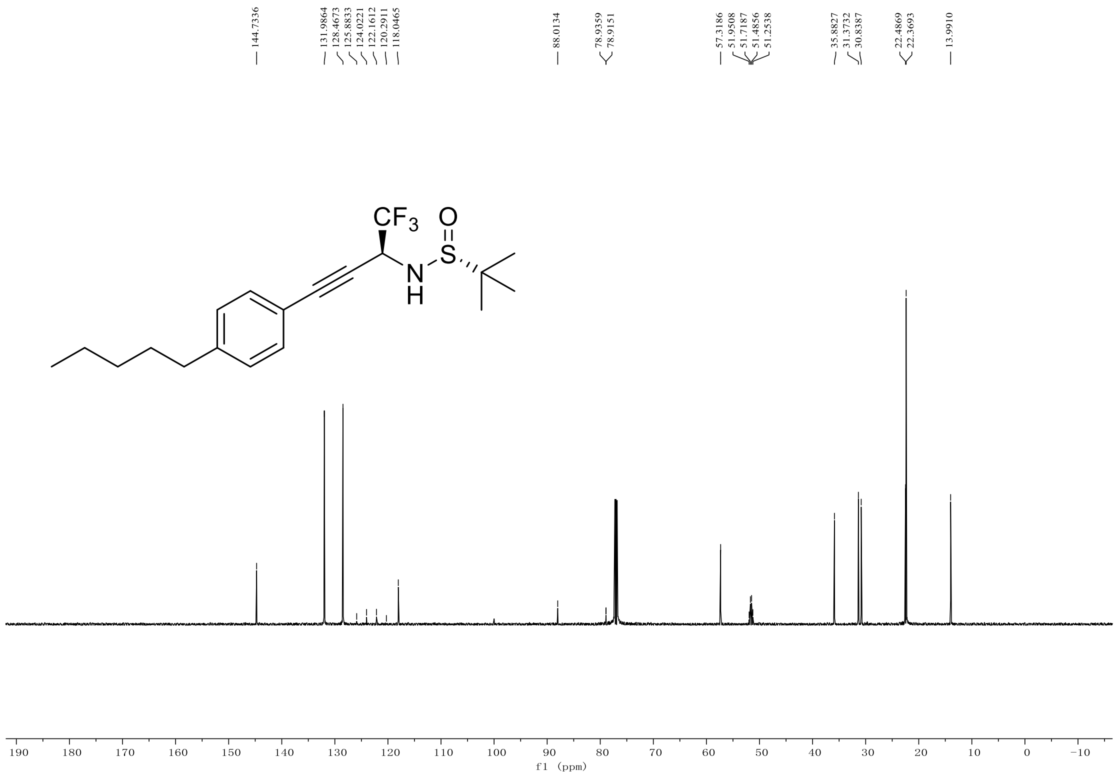

^{19}F NMR (376 MHz, $CDCl_3$) of (R_s, S)-**3g**:

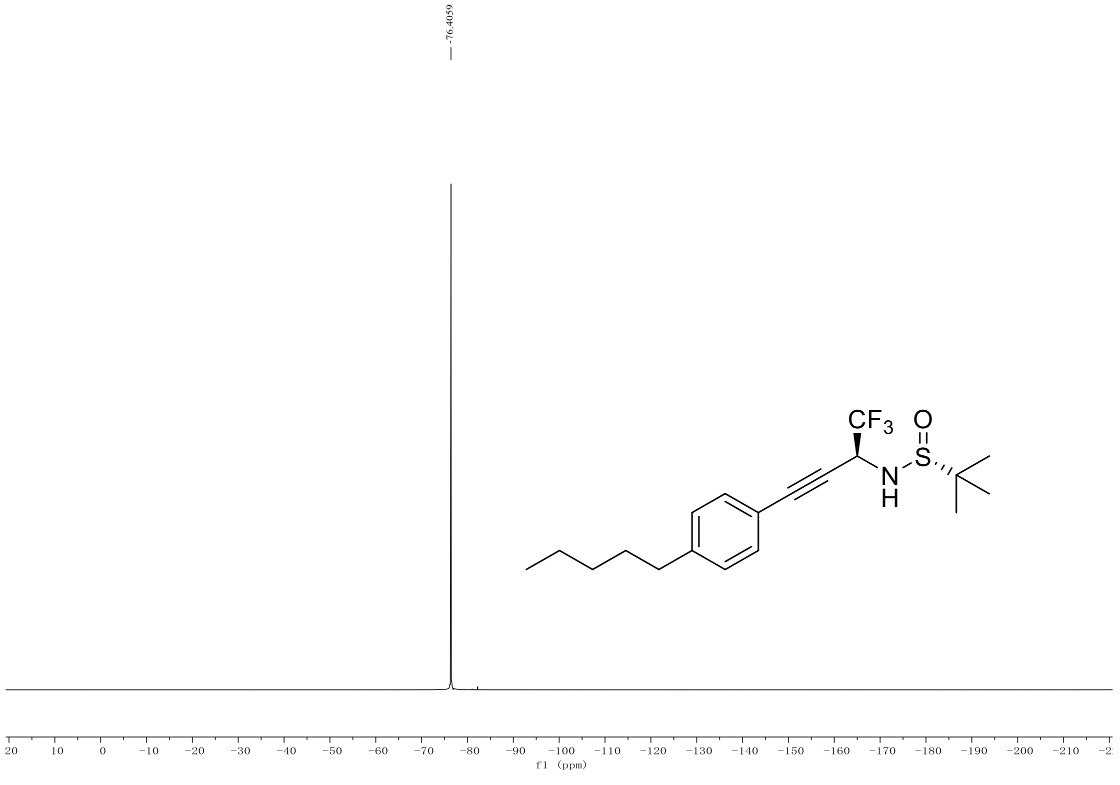

^{1}H NMR (600 MHz, $CDCl_3$) of (R_s, R)-**3h**:

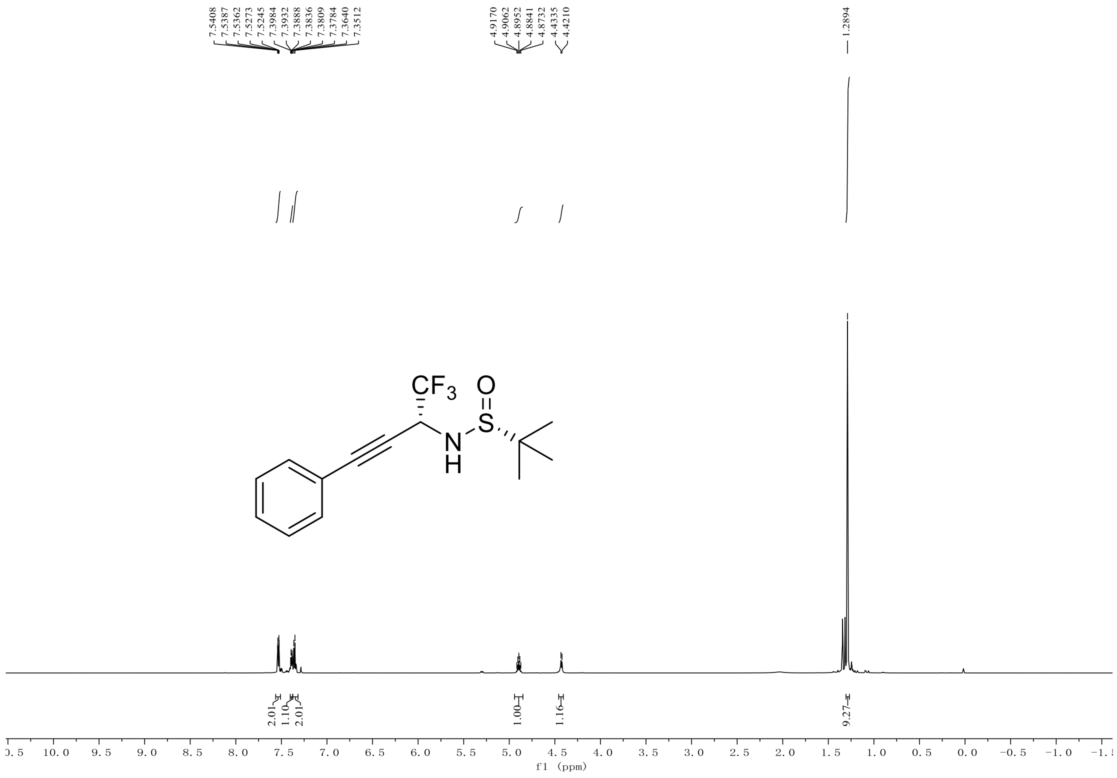

^{13}C NMR (150 MHz, $CDCl_3$) of (R_s, R)-**3h**:

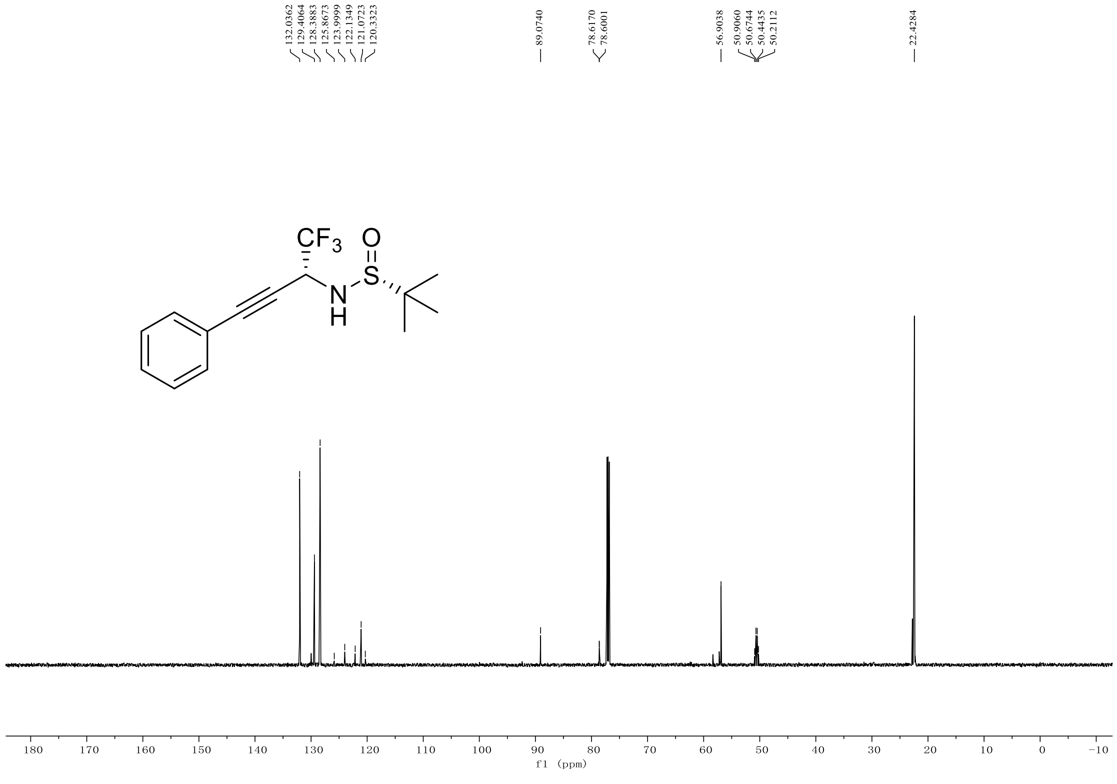

^{19}F NMR (565 MHz, $CDCl_3$) of (R_s, R)-**3h**:

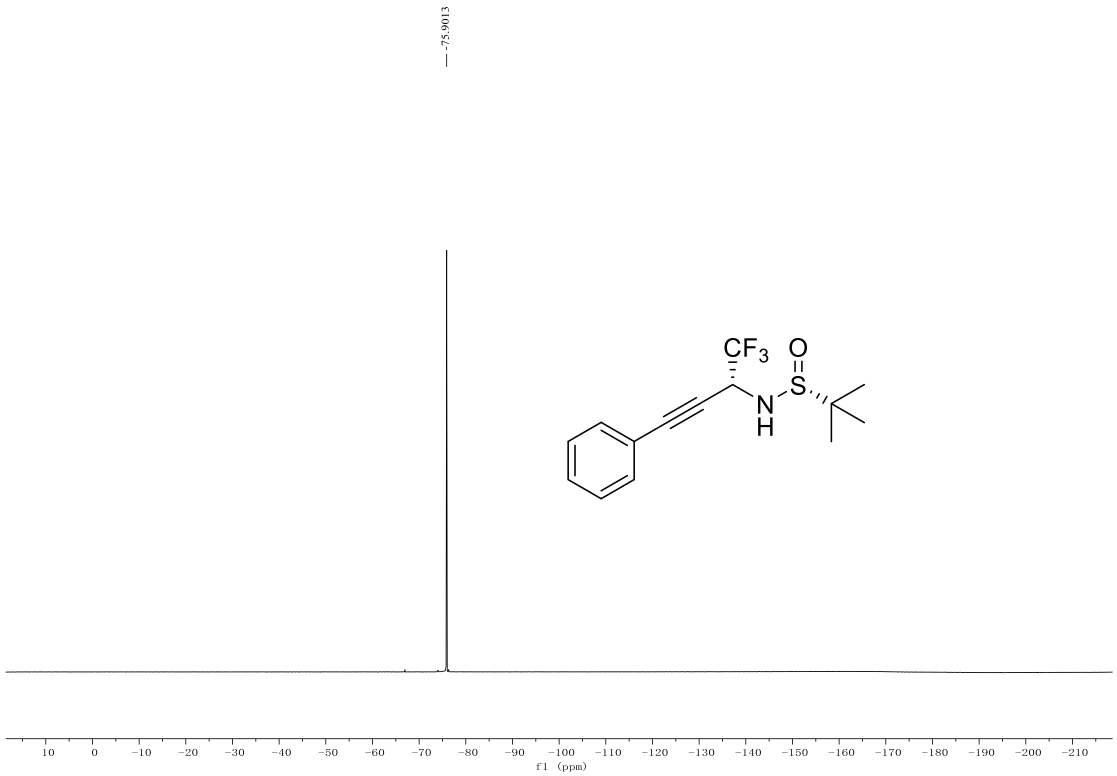

^{1}H NMR (600 MHz, $CDCl_3$) of (R_s, S)-**3h**:

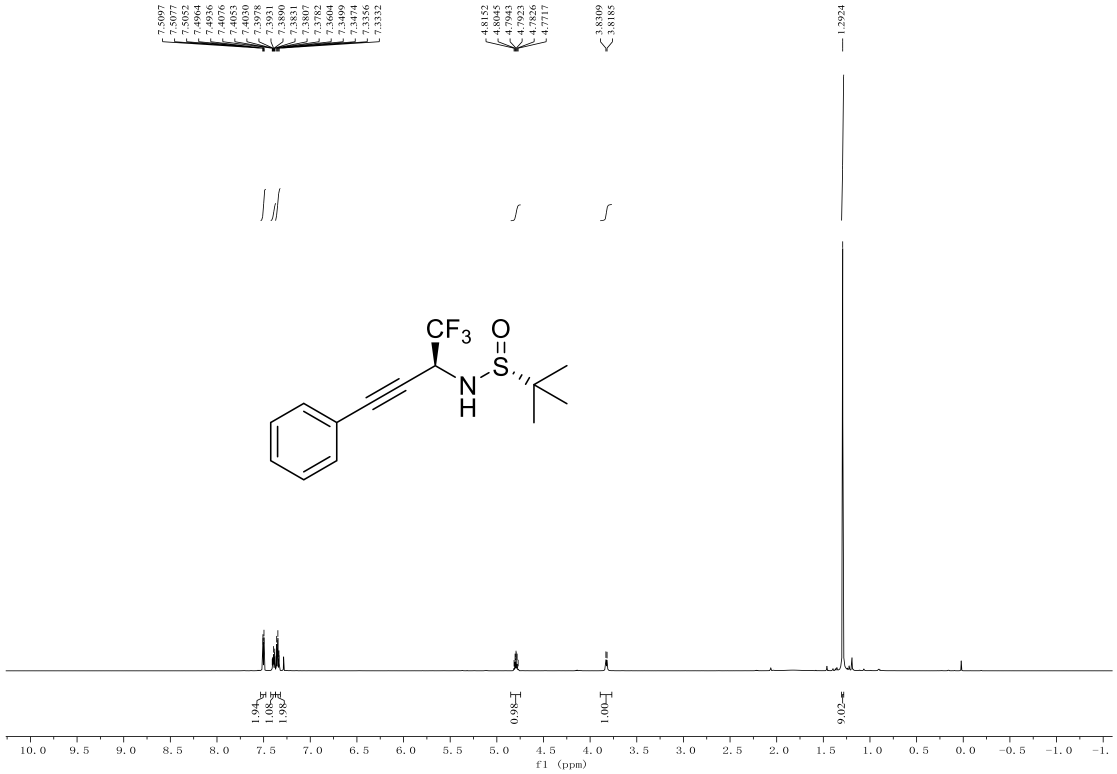

^{13}C NMR (150 MHz, $CDCl_3$) of (R_s, S)-**3h**:

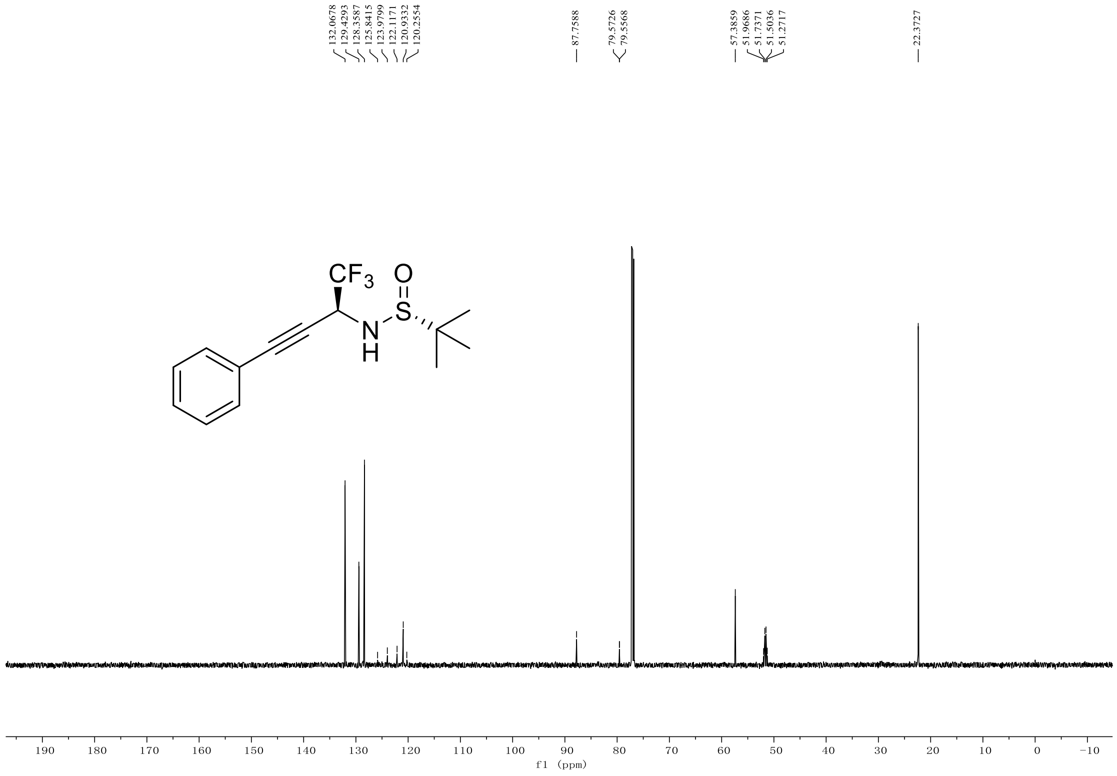

^{19}F NMR (565 MHz, $CDCl_3$) of (R_s, S)-**3h**:

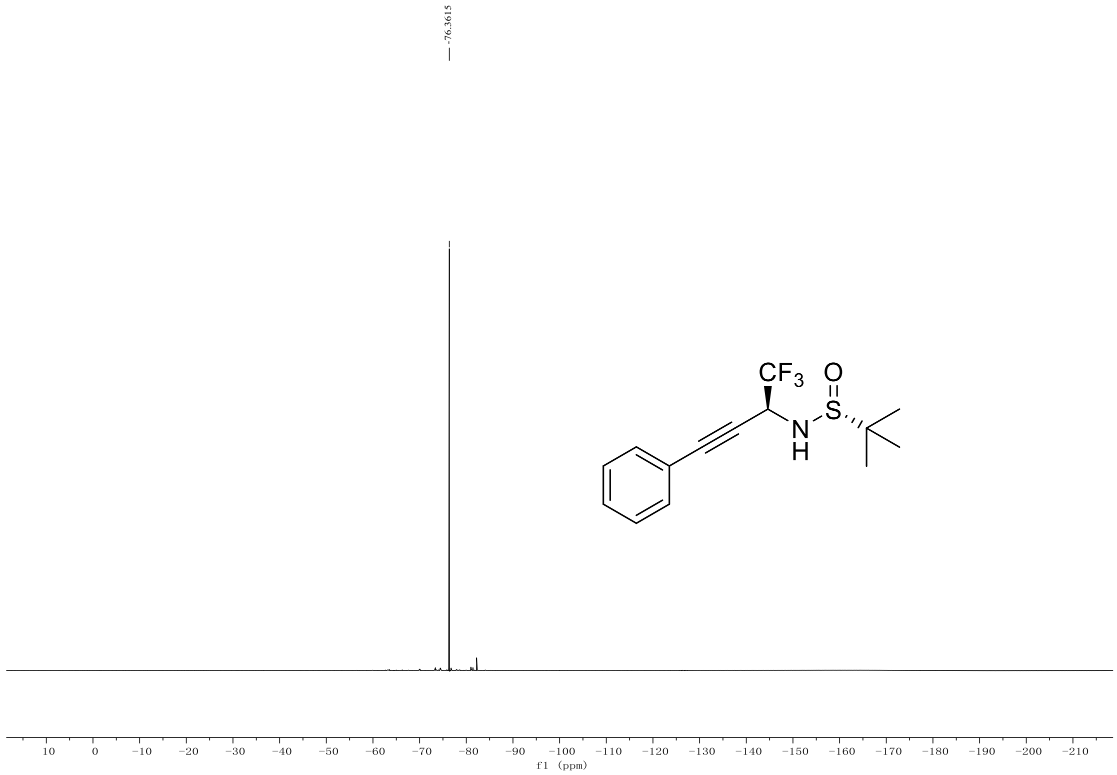

^{1}H NMR (600 MHz, $CDCl_3$) of (R_s, R)-**3i**:

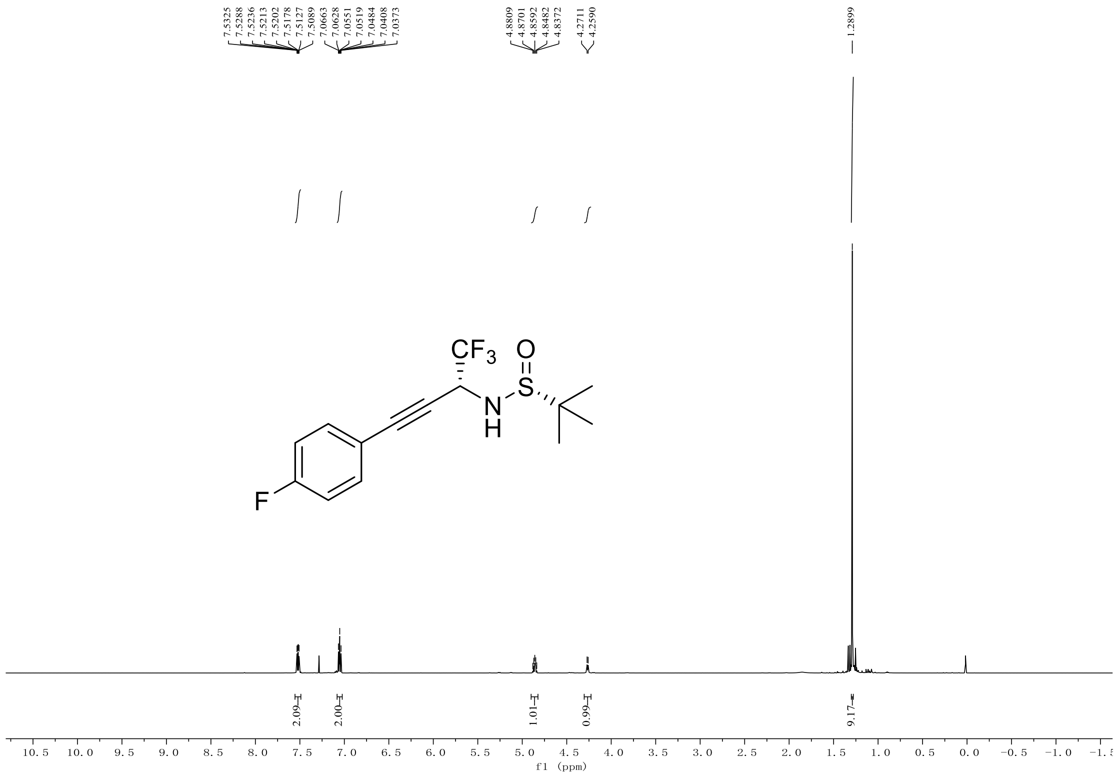

^{13}C NMR (150 MHz, $CDCl_3$) of (R_s, R)-**3i**:

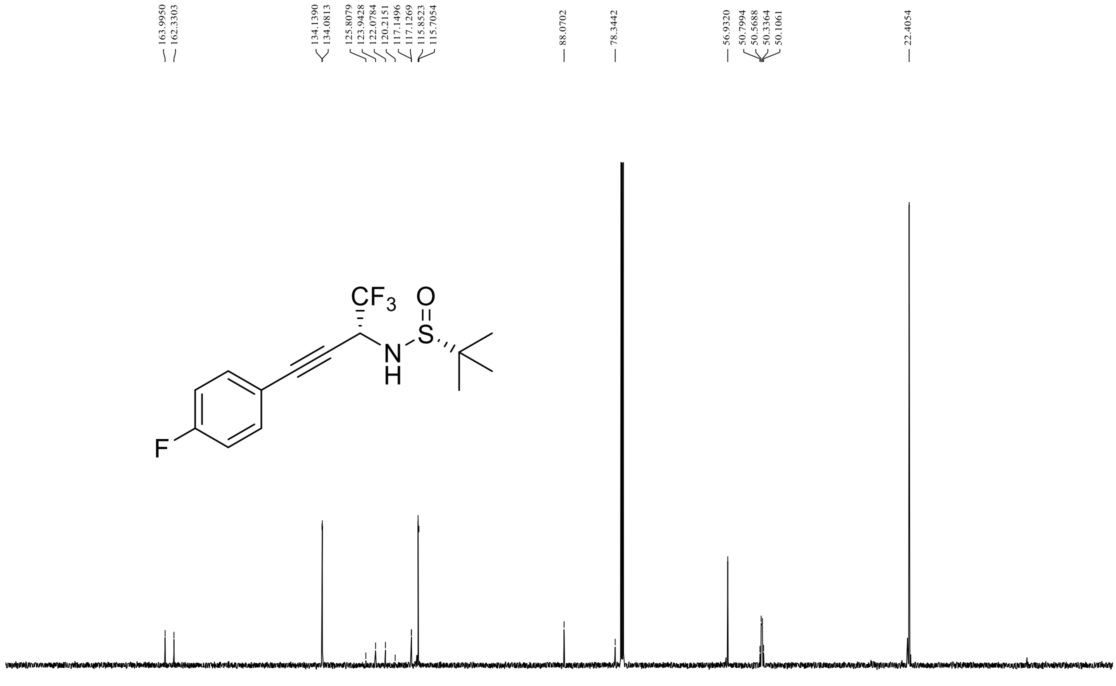

^{19}F NMR (565 MHz, $CDCl_3$) of (R_s, R)-**3i**:

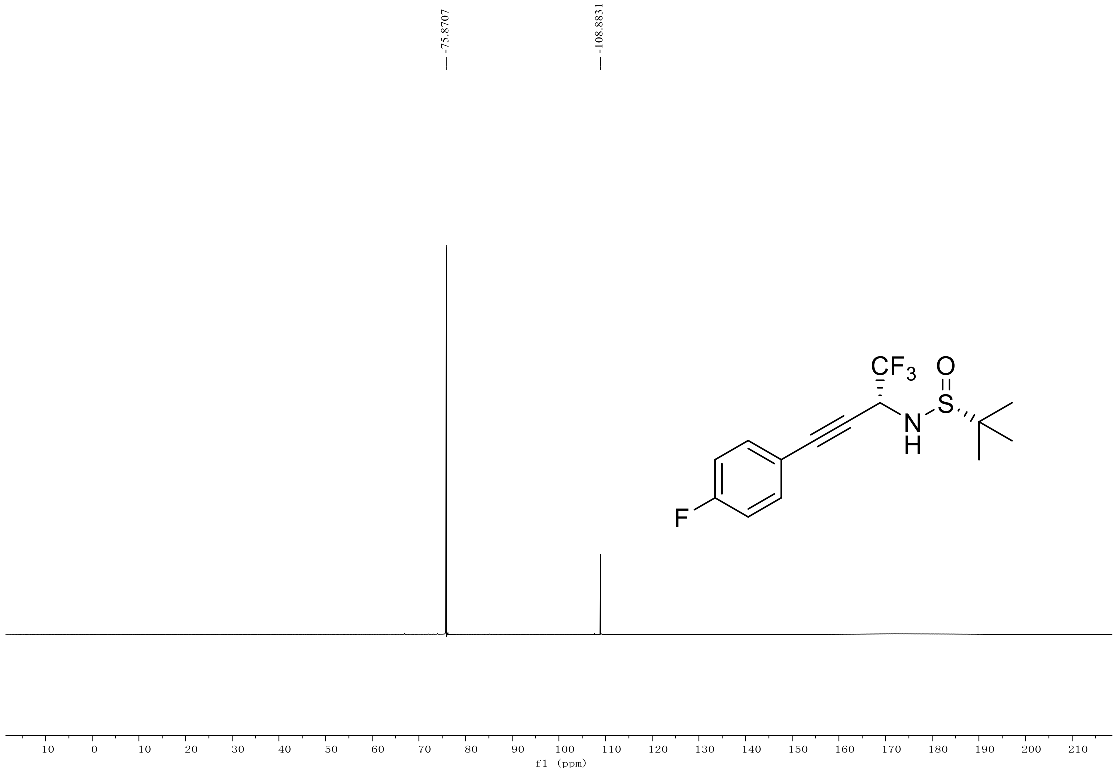

^{1}H NMR (600 MHz, $CDCl_3$) of (R_s, S)-**3i**:

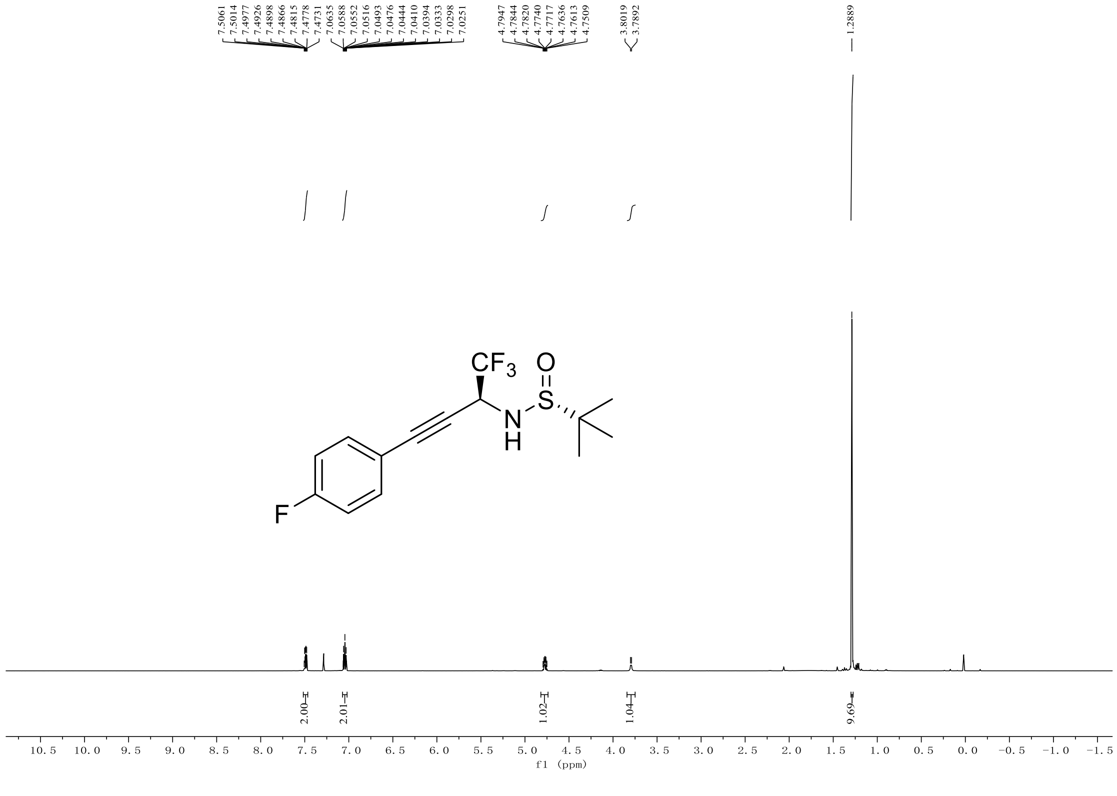

^{13}C NMR (150 MHz, $CDCl_3$) of (R_s, S)-**3i**:

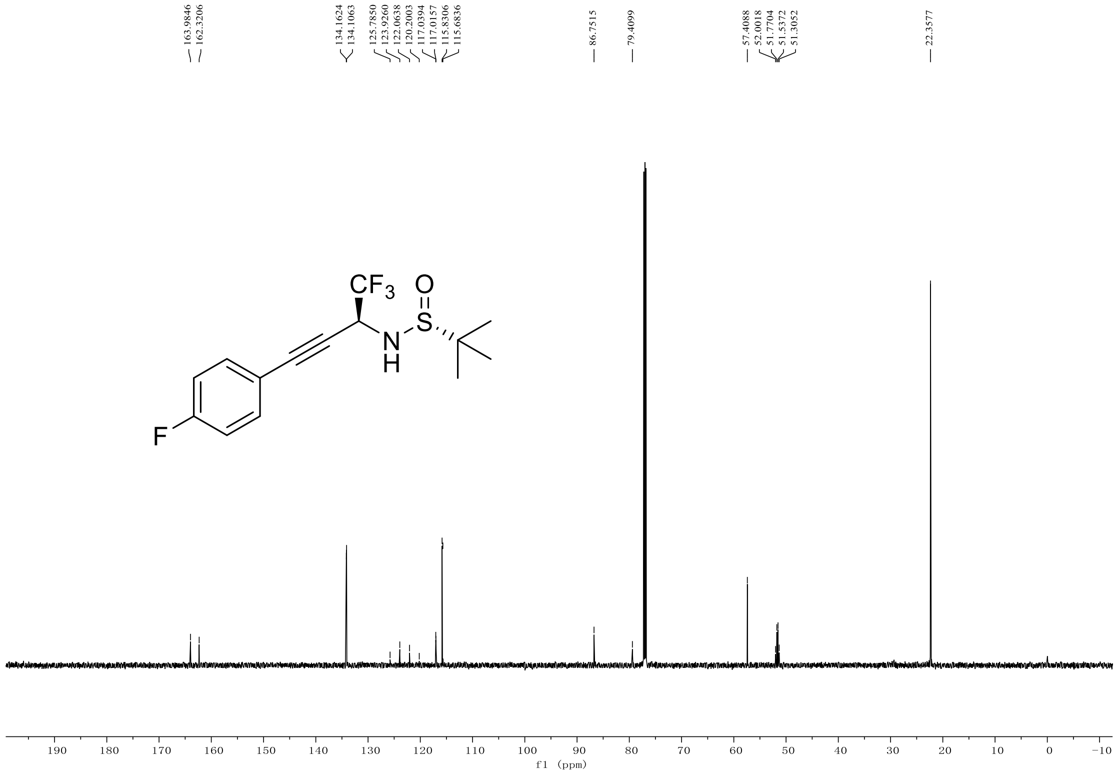

^{19}F NMR (565 MHz, $CDCl_3$) of (R_s, S)-**3i**:

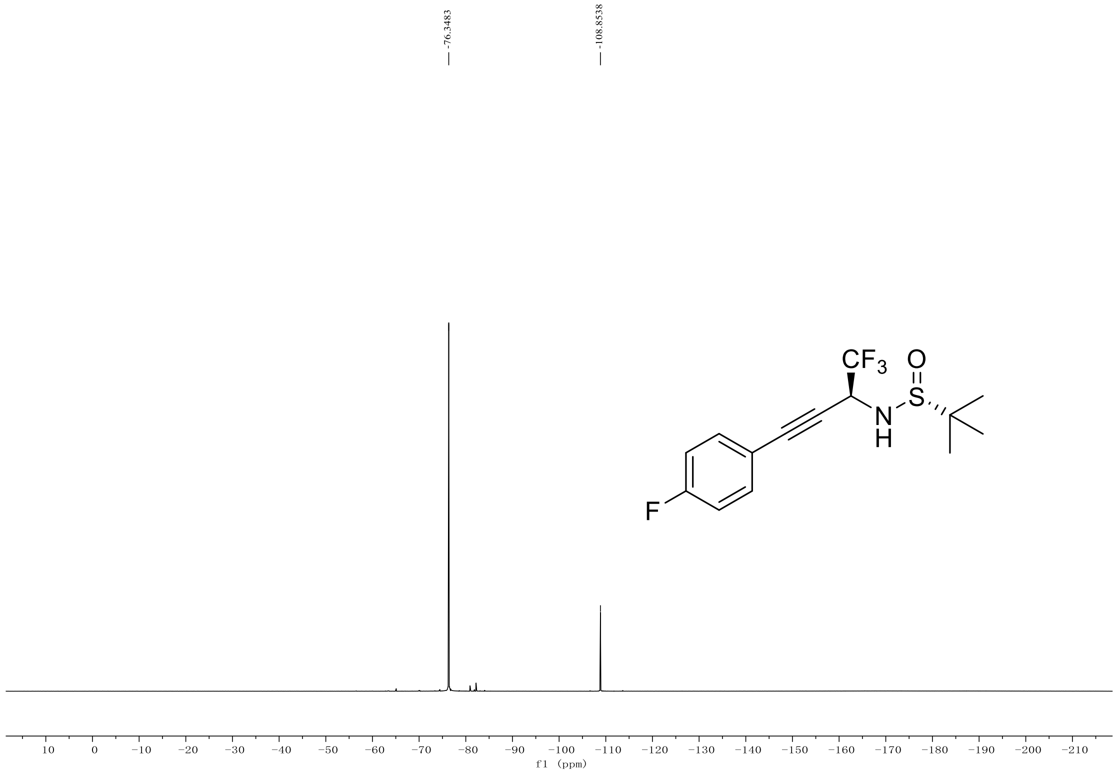

^{1}H NMR (600 MHz, $CDCl_3$) of (R_s, R)-**3j**:

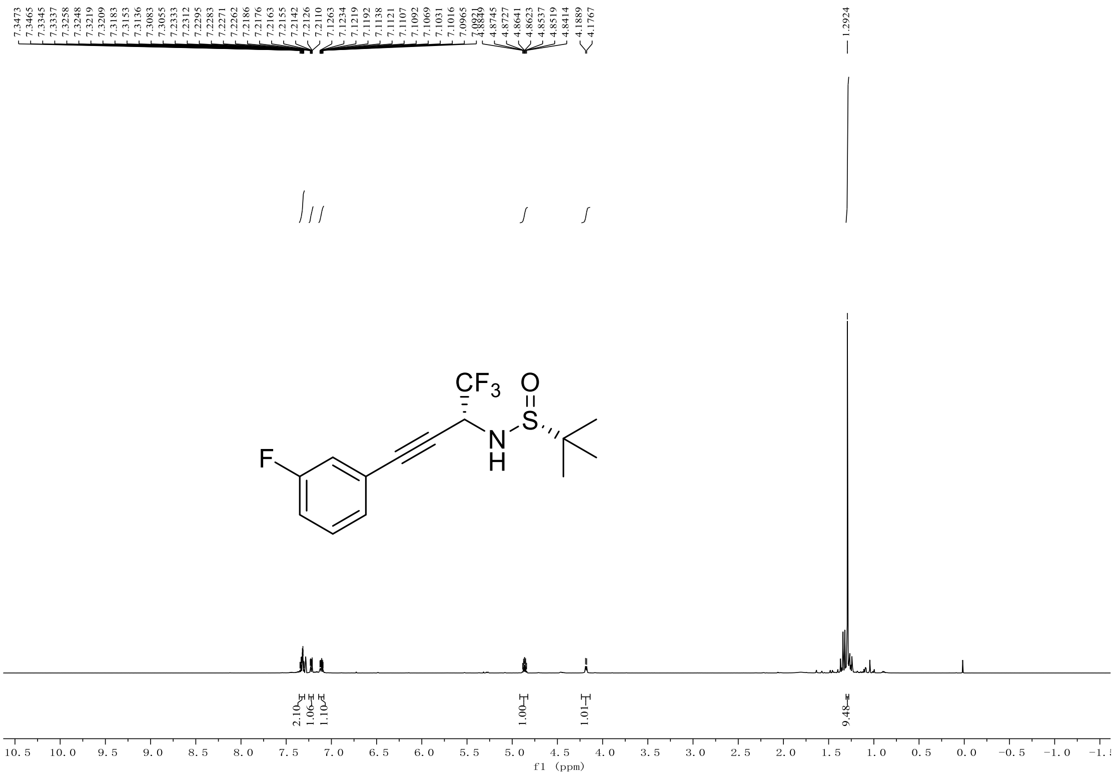

^{13}C NMR (150 MHz, $CDCl_3$) of (R_s, R)-**3j**:

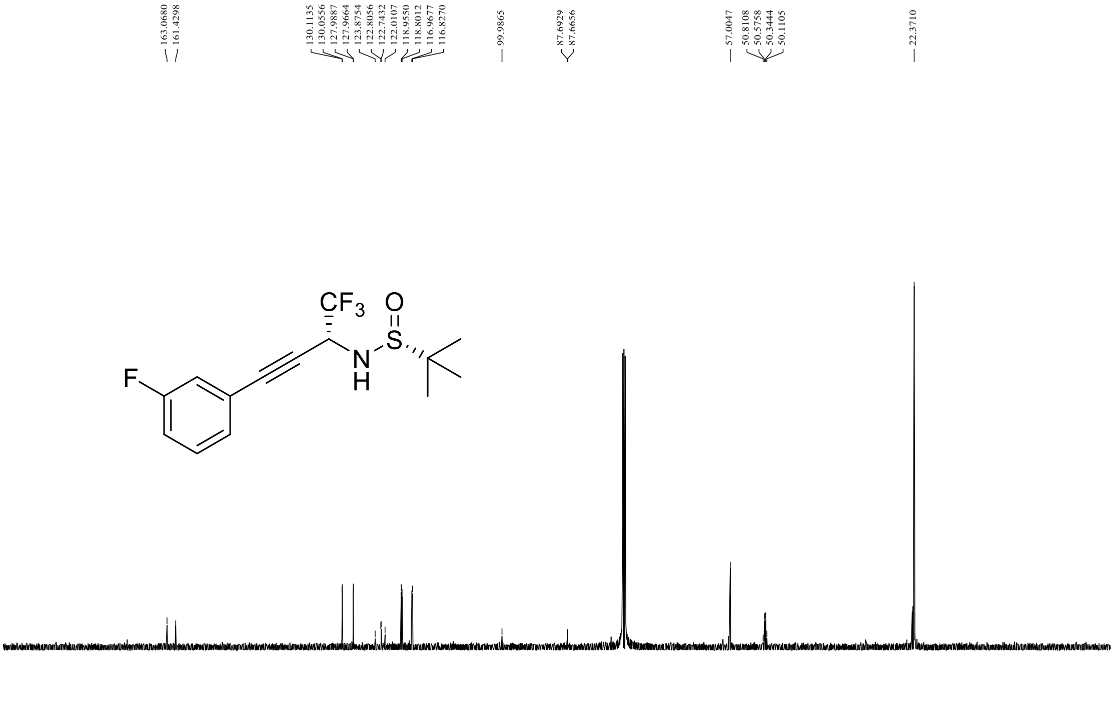

^{19}F NMR (565 MHz, $CDCl_3$) of (R_s, R)-**3j**:

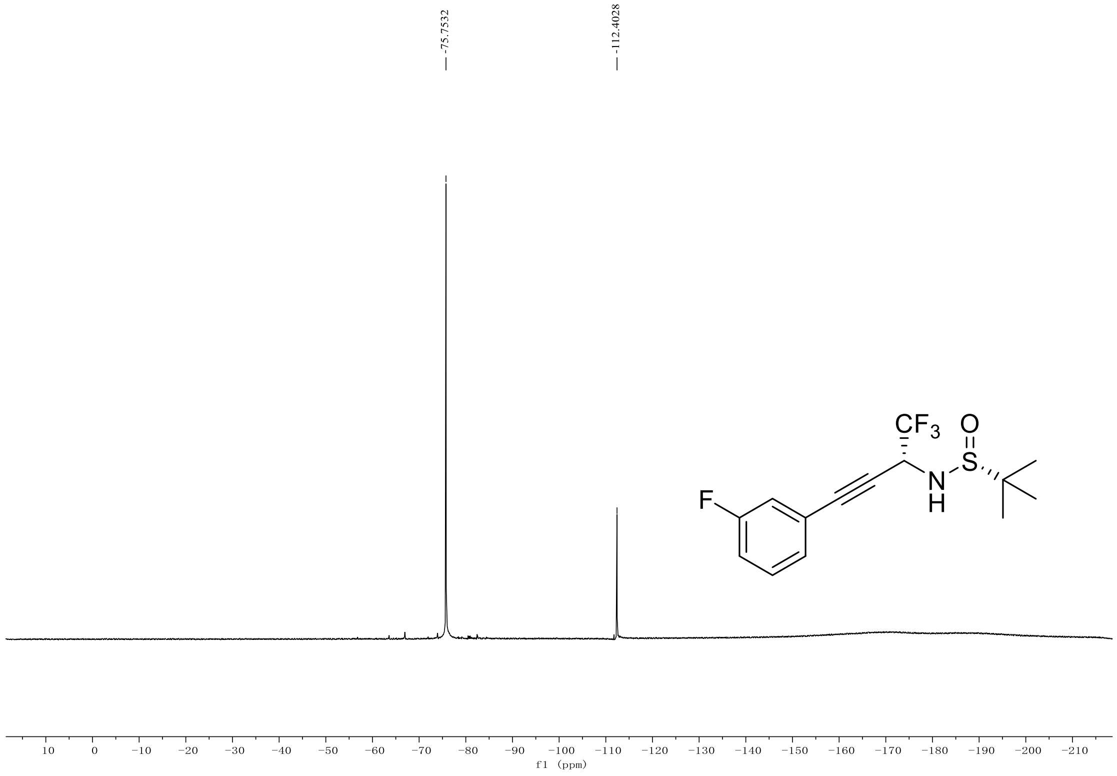

^{1}H NMR (600 MHz, $CDCl_3$) of (R_s, S)-**3j**:

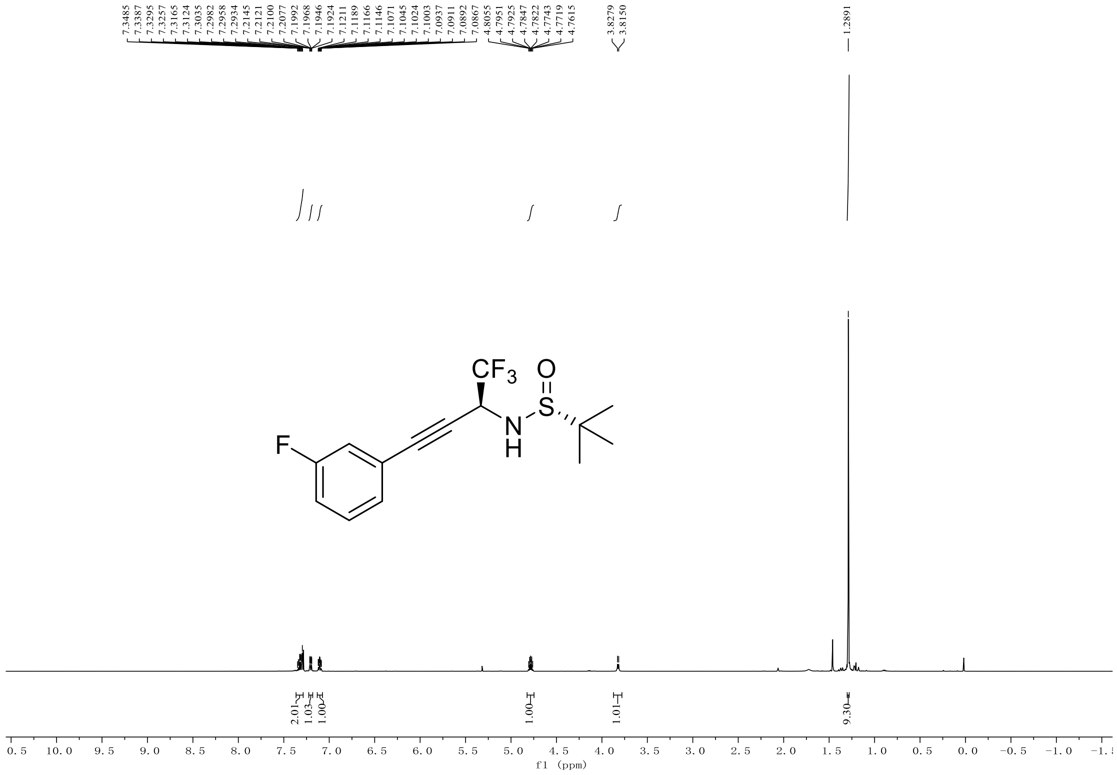

^{13}C NMR (150 MHz, $CDCl_3$) of (R_s, S)-**3j**:

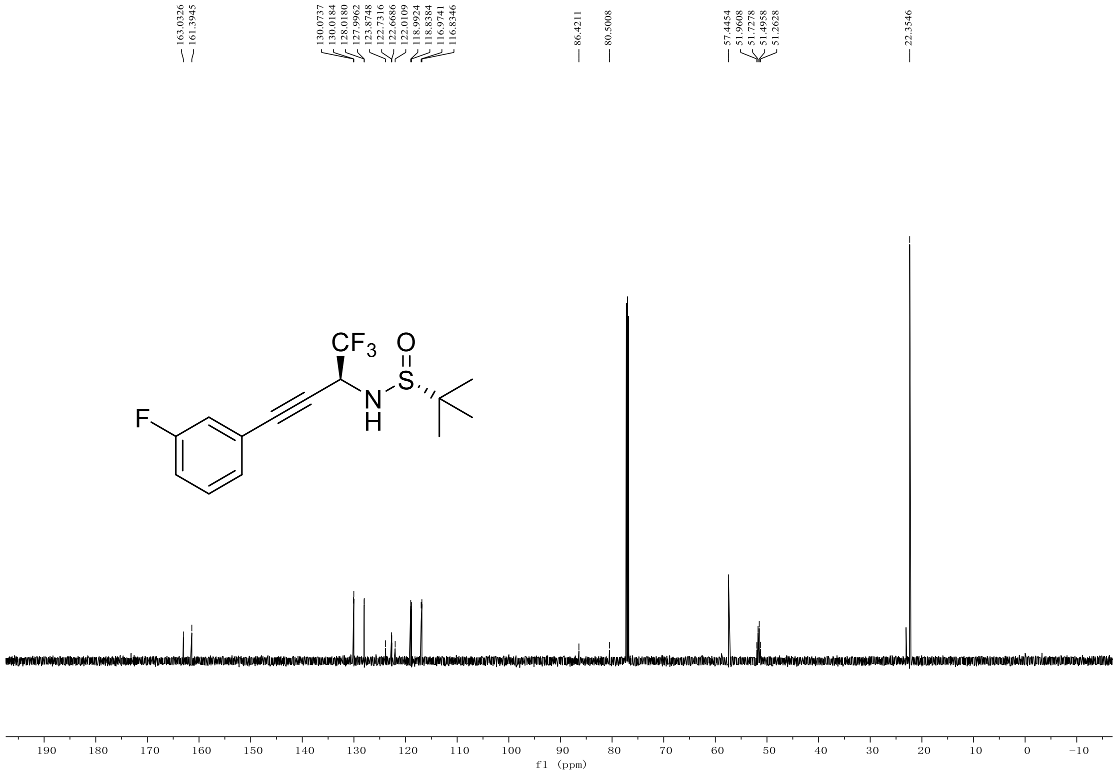

^{19}F NMR (565 MHz, $CDCl_3$) of (R_s, S)-**3j**:

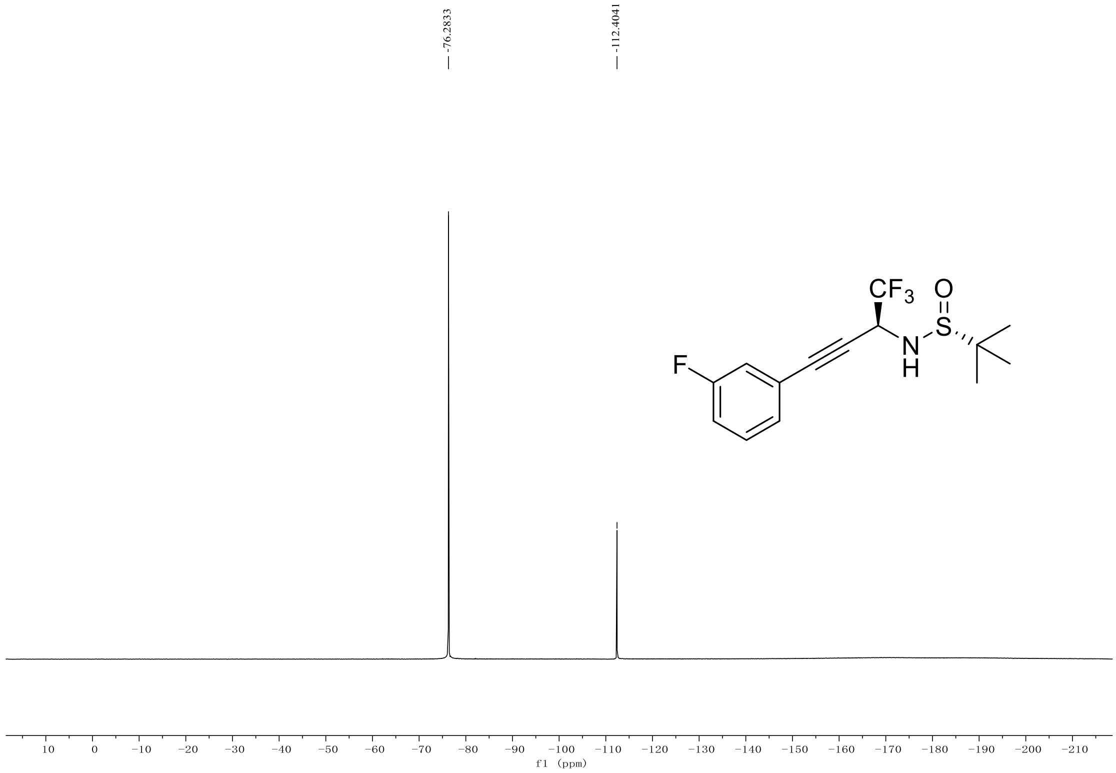

^{1}H NMR (600 MHz, $CDCl_3$) of (R_s, R)-**3k**:

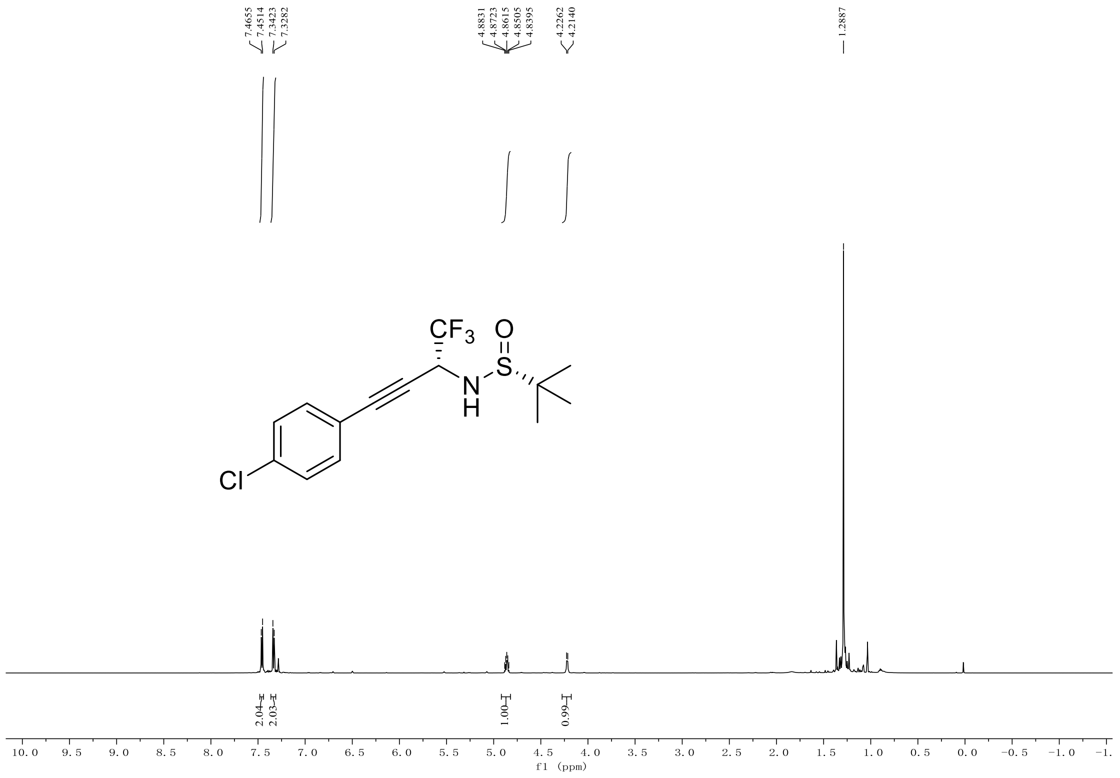

^{13}C NMR (150 MHz, $CDCl_3$) of (R_s, R)-**3k**:

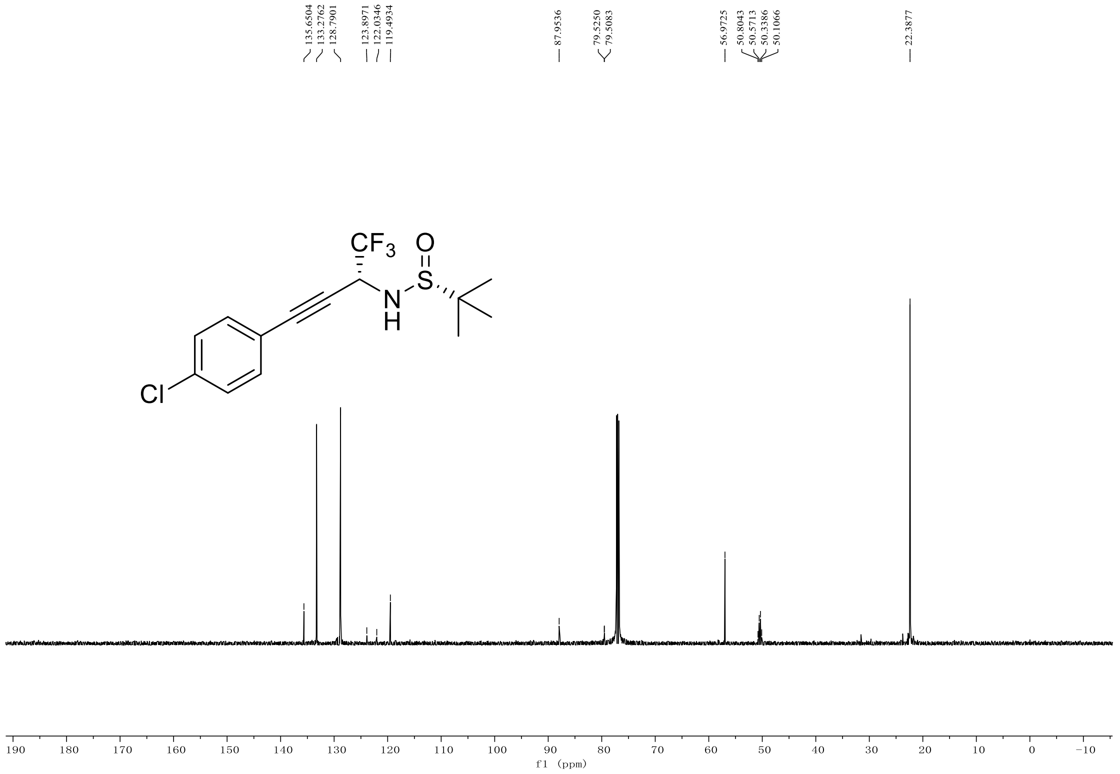

^{19}F NMR (565 MHz, $CDCl_3$) of (*R*s, *R*)-**3k**:

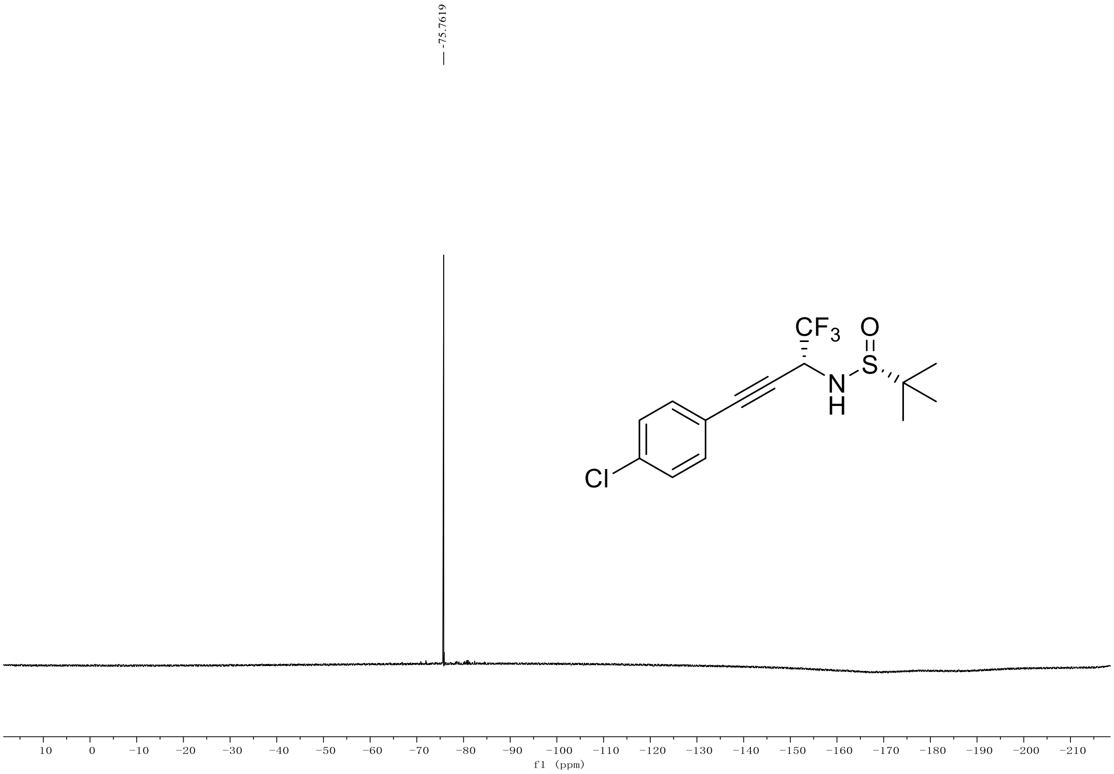

^{1}H NMR (600 MHz, $CDCl_3$) of (*R*s, *S*)-**3k**:

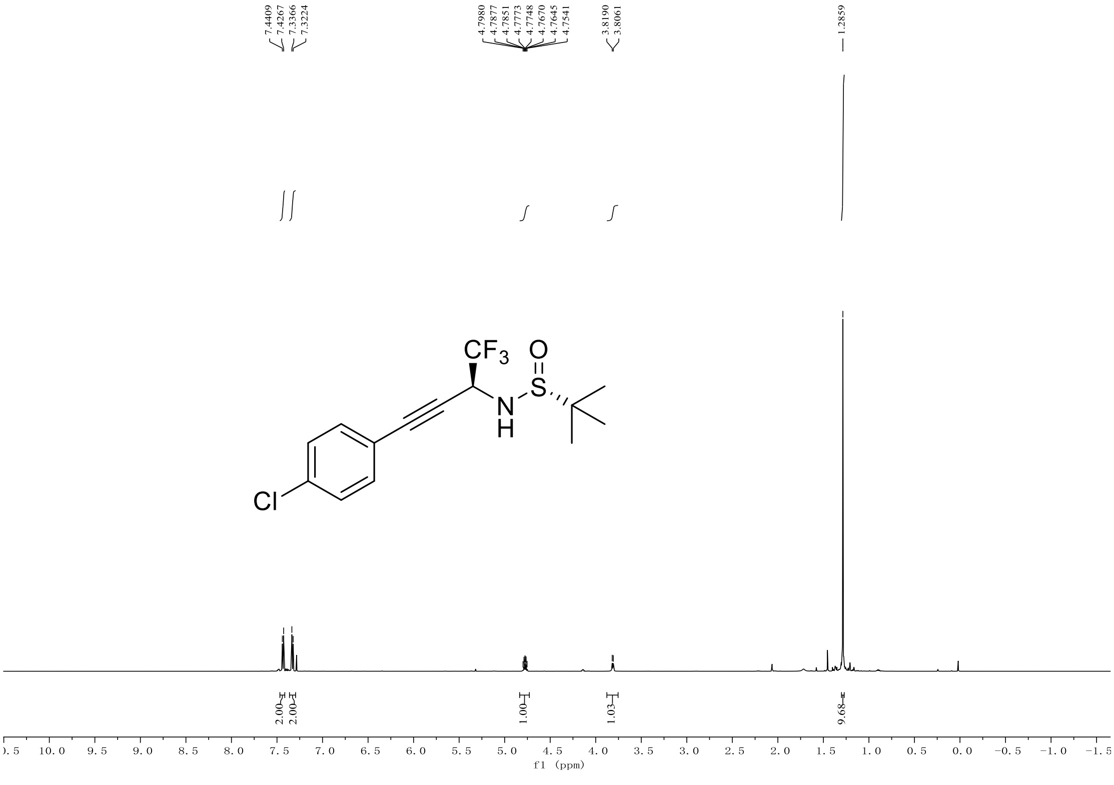

^{13}C NMR (150 MHz, $CDCl_3$) of (R_s, S)-**3k**:

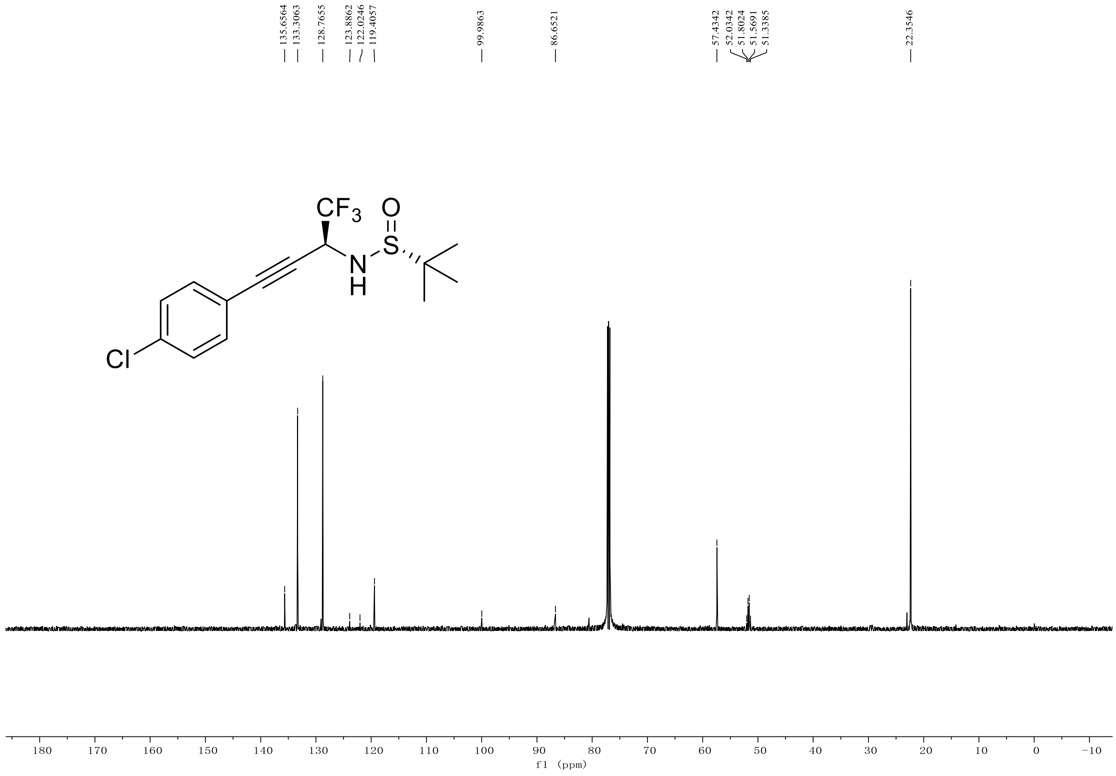

^{19}F NMR (565 MHz, $CDCl_3$) of (R_s, S)-**3k**:

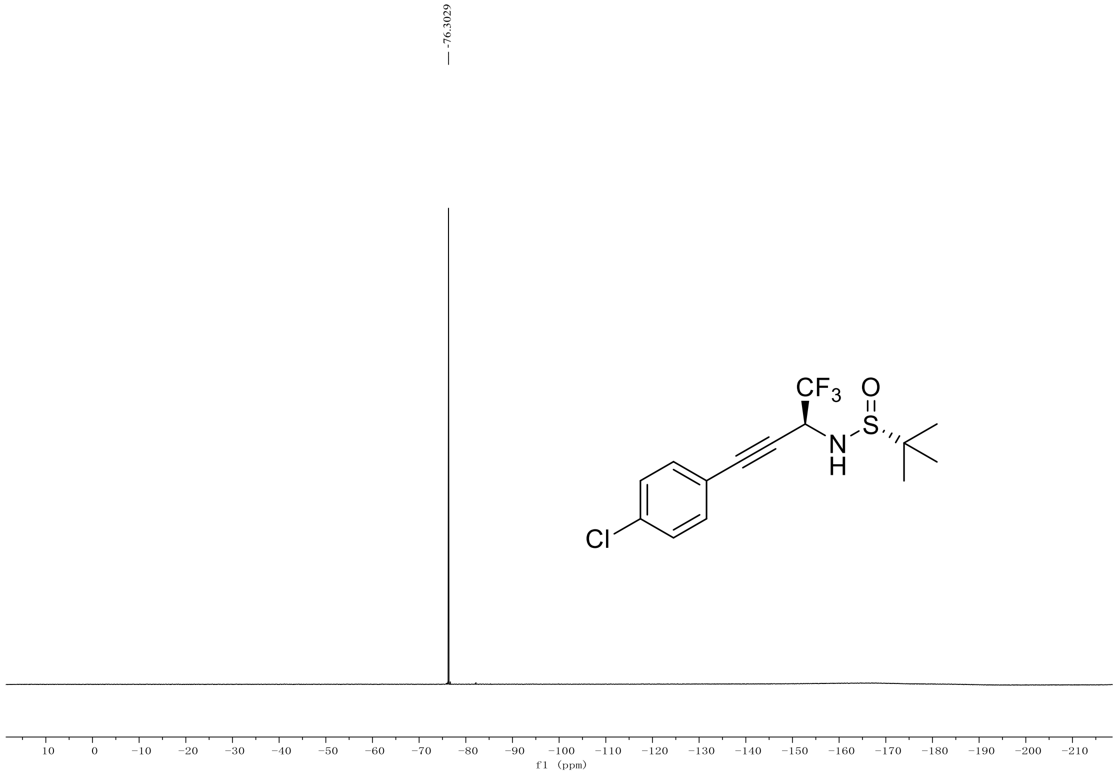

^{1}H NMR (400 MHz, $CDCl_3$) of (R_s, R)-**3l**:

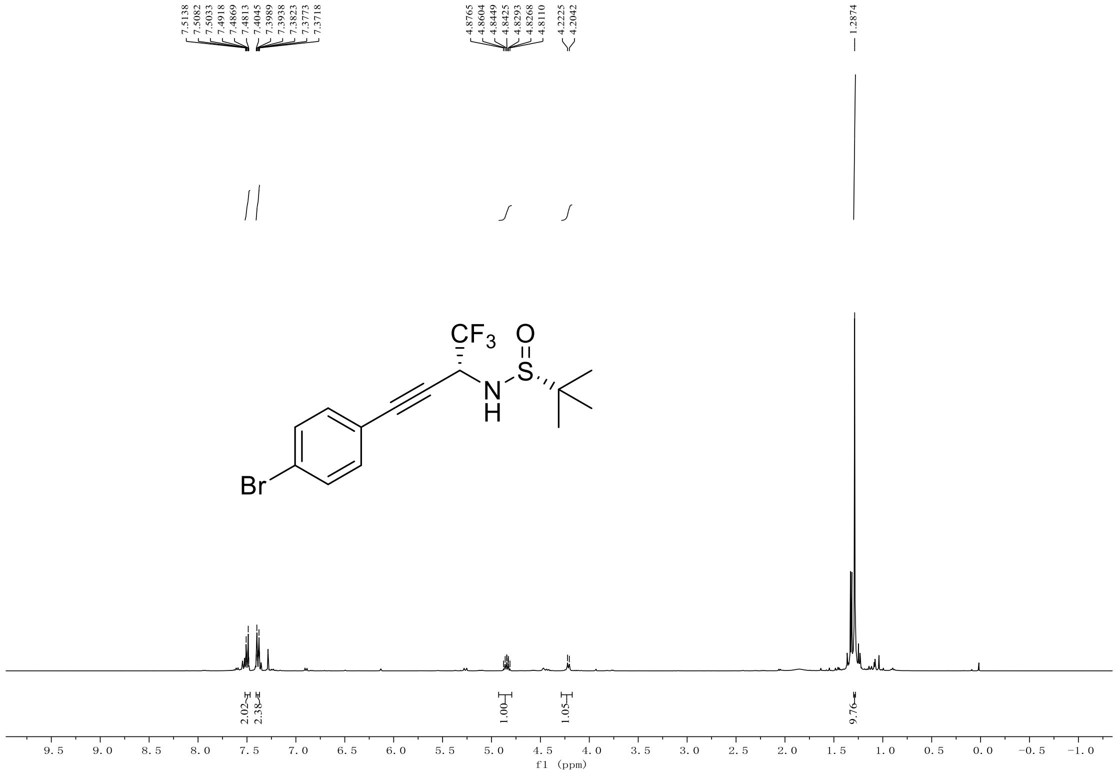

^{13}C NMR (150 MHz, $CDCl_3$) of (R_s, R)-**3l**:

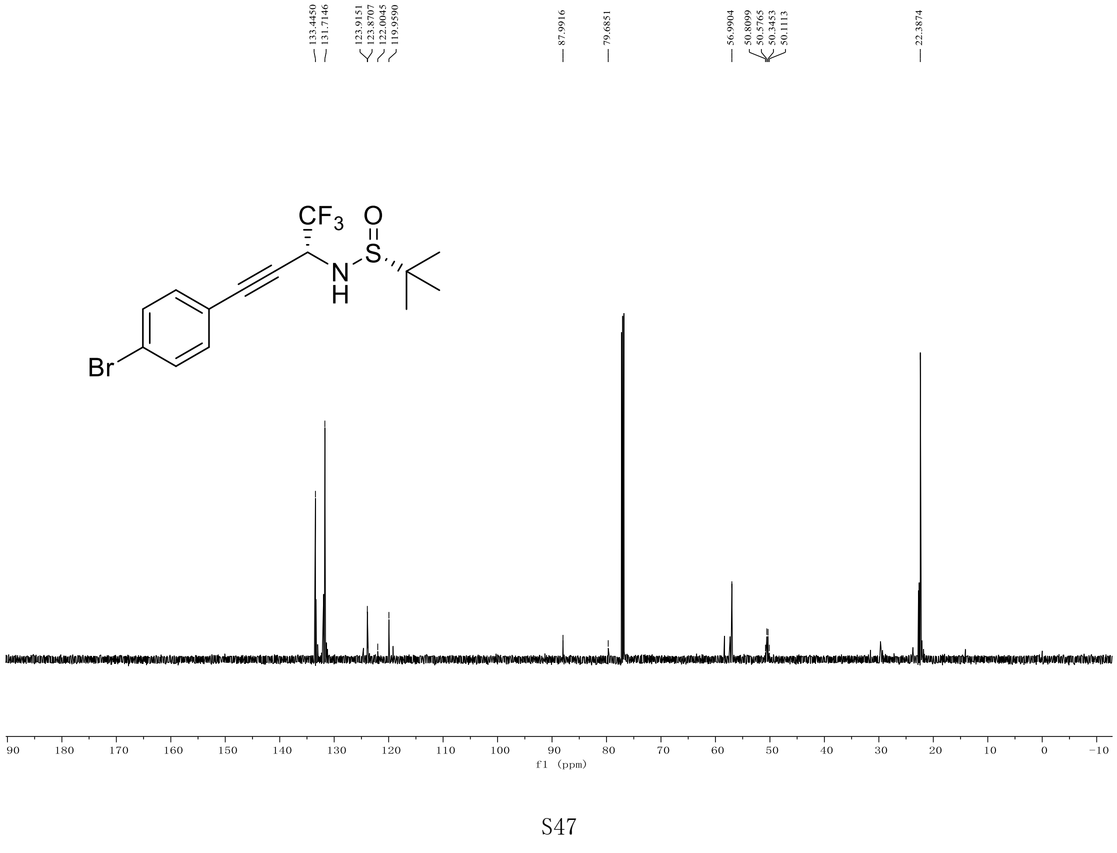

^{19}F NMR (565 MHz, $CDCl_3$) of (R_s, R)-**3l**:

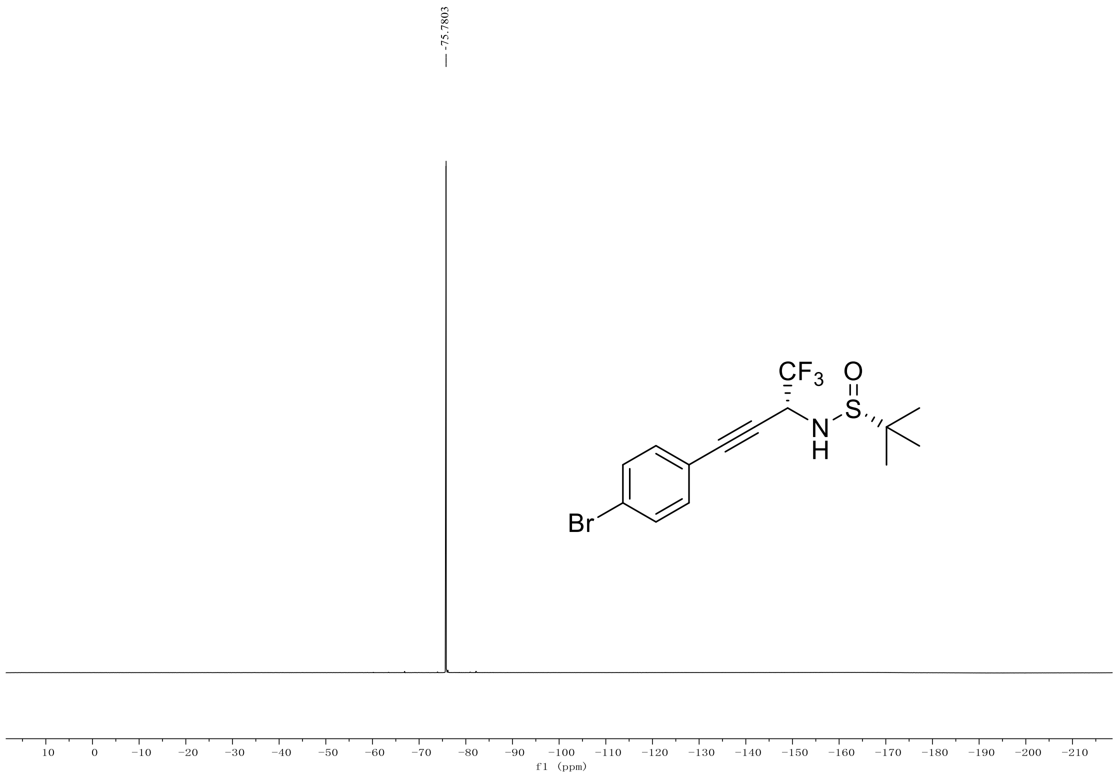

^{1}H NMR (400 MHz, $CDCl_3$) of (R_s, S)-**3l**:

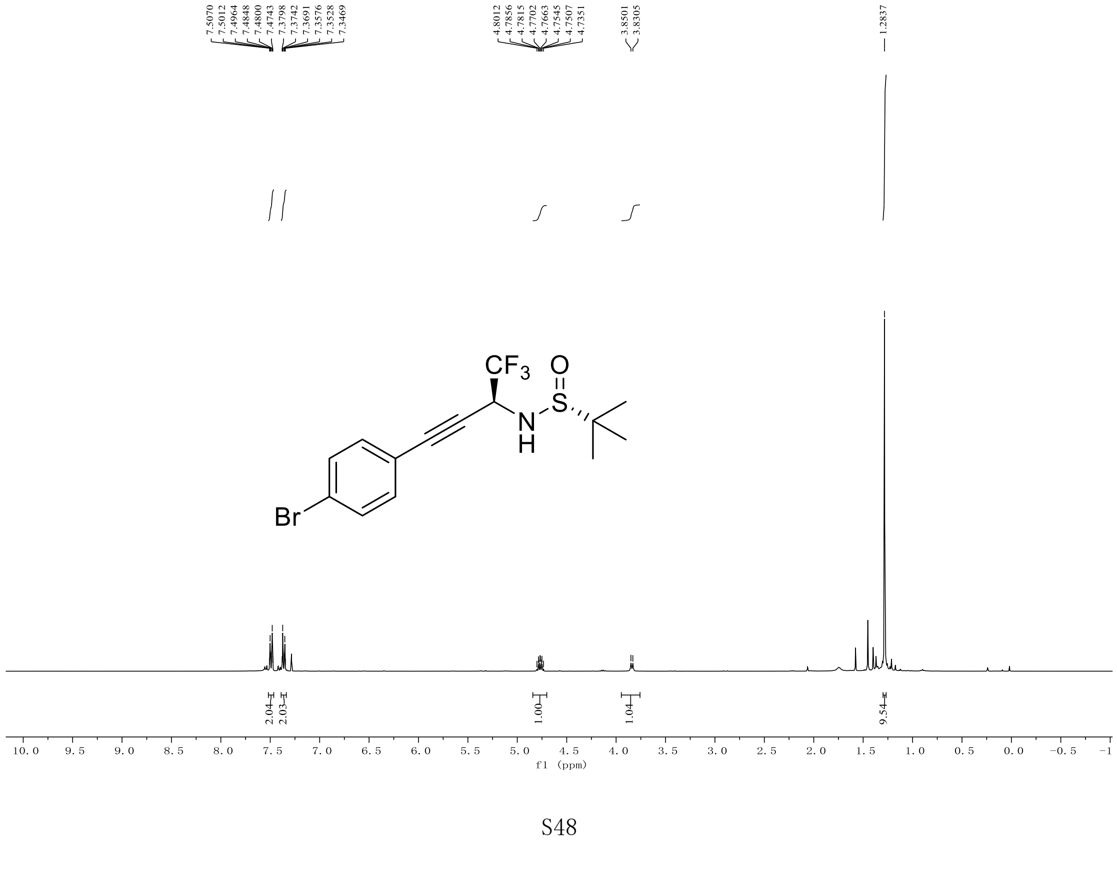

^{13}C NMR (150 MHz, $CDCl_3$) of (R_s, S)-**3l**:

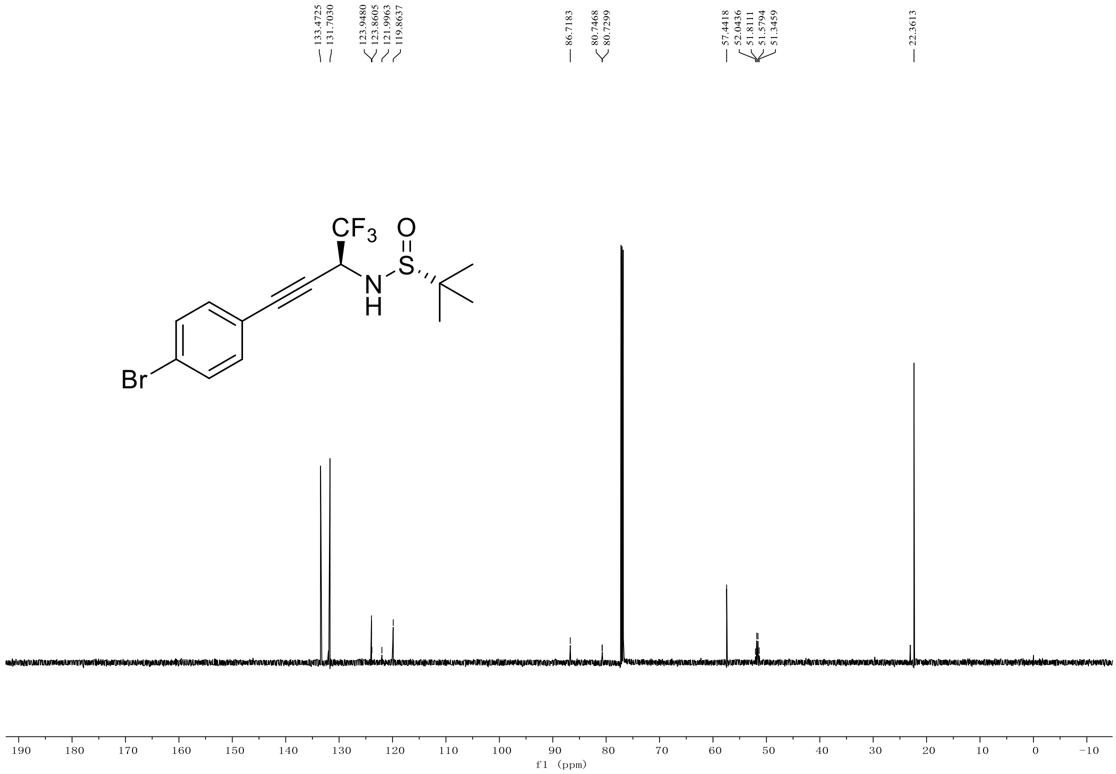

^{19}F NMR (565 MHz, $CDCl_3$) of (R_s, S)-**3l**:

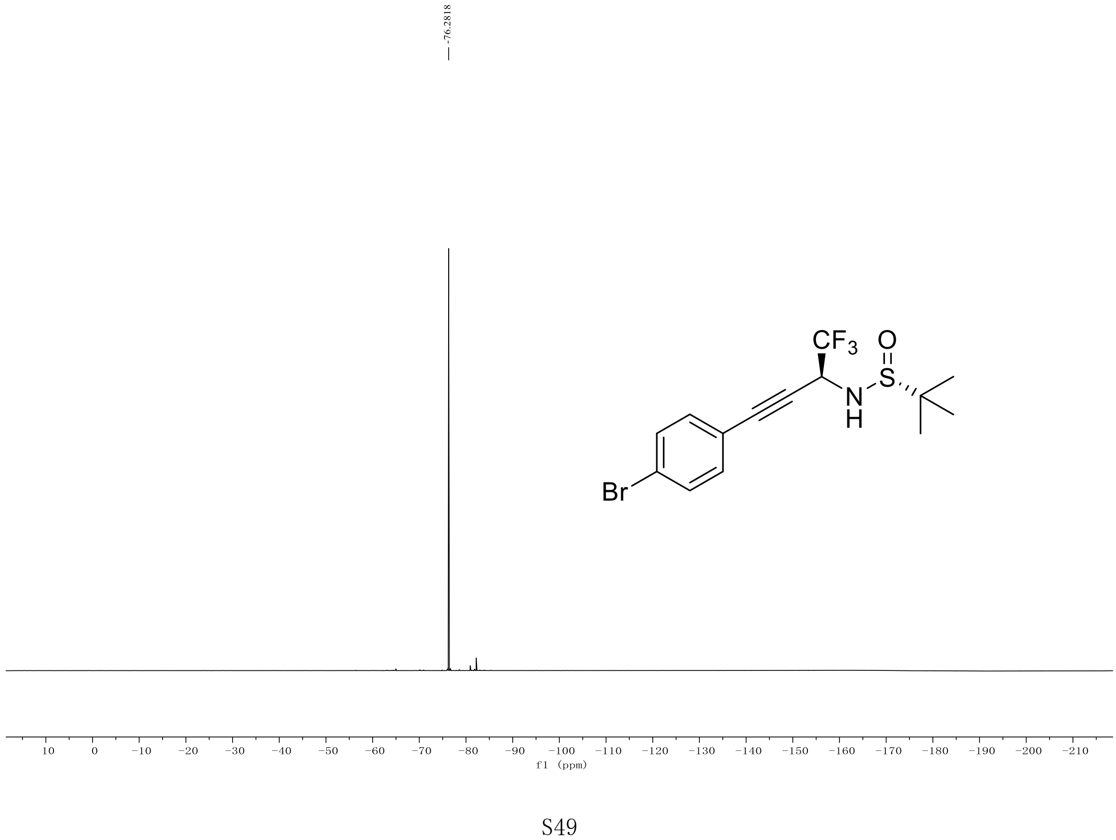

^{1}H NMR (400 MHz, $CDCl_3$) of (R_s, R)-**3m**:

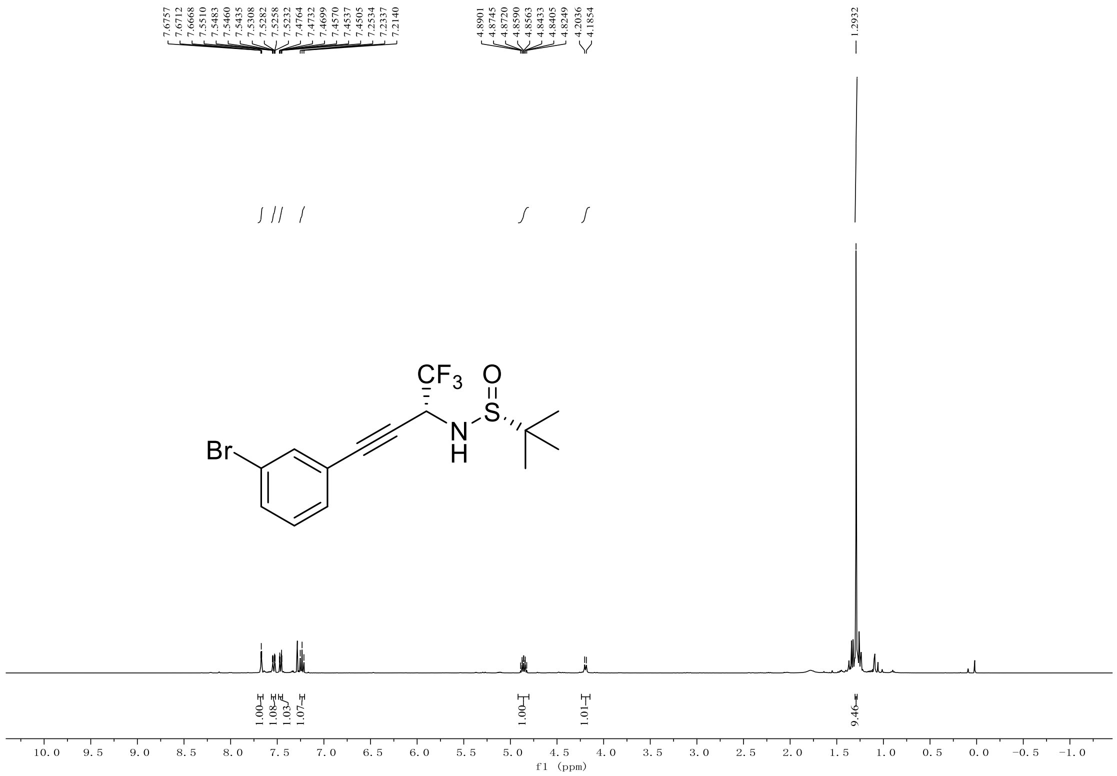

^{13}C NMR (150 MHz, $CDCl_3$) of (R_s, R)-**3m**:

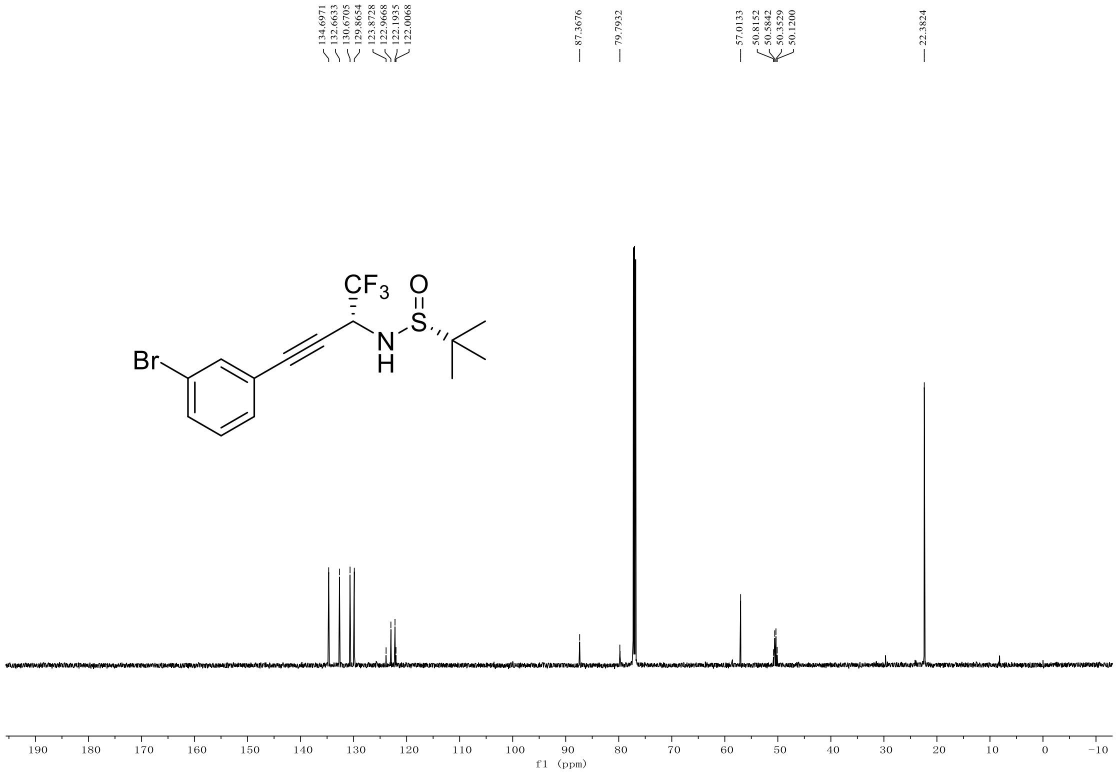

^{19}F NMR (565 MHz, $CDCl_3$) of (R_s, R)-**3m**:

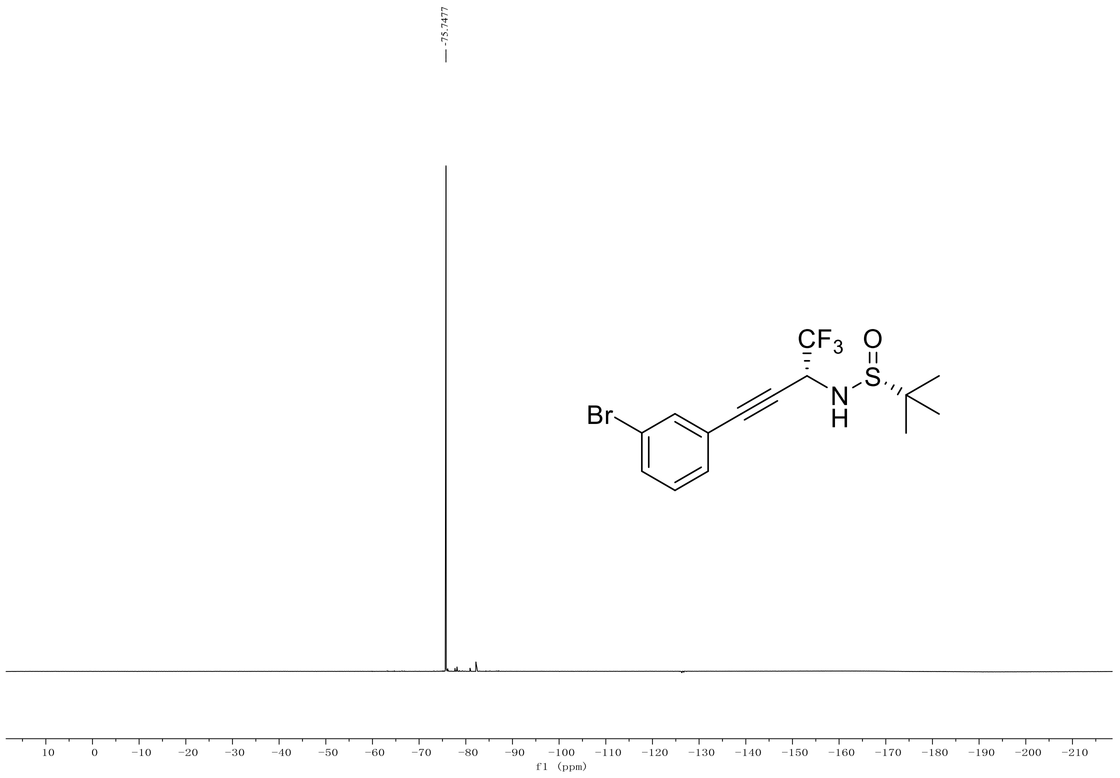

^{1}H NMR (400 MHz, $CDCl_3$) of (R_s, S)-**3m**:

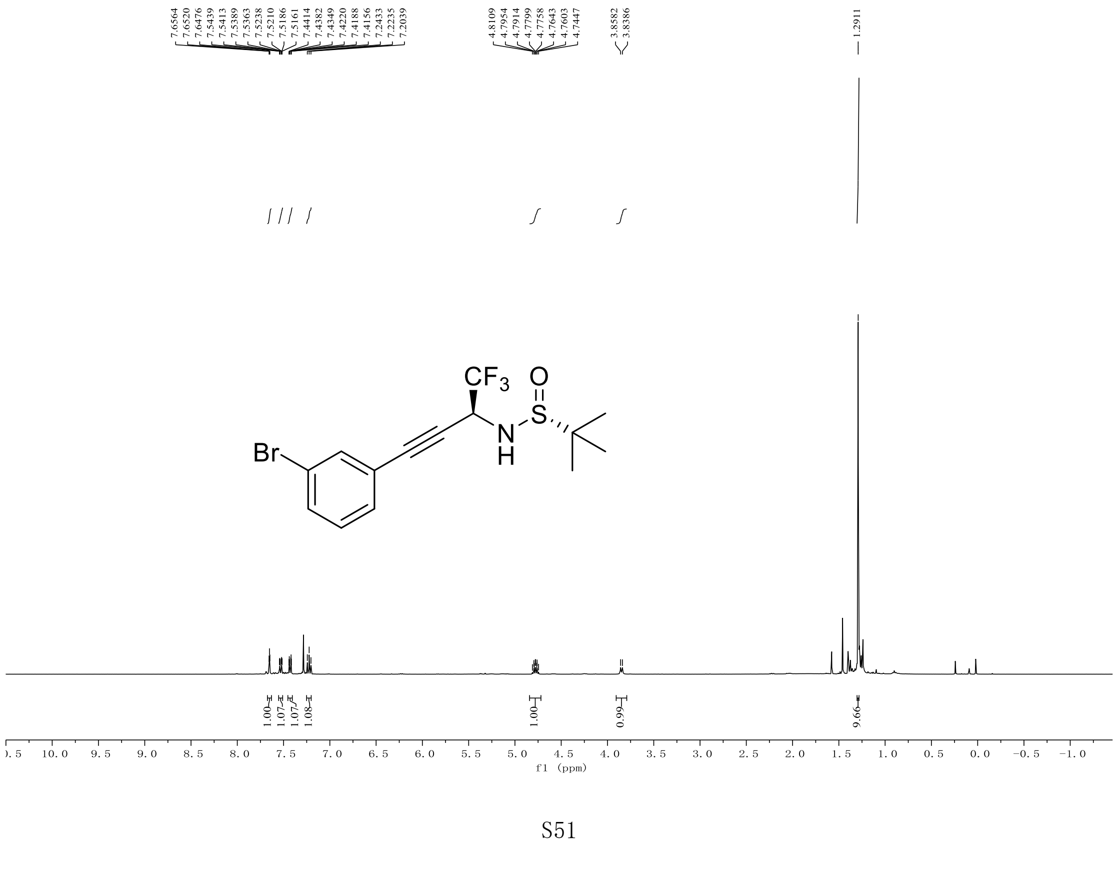

^{13}C NMR (150 MHz, $CDCl_3$) of (*R*s, *S*)-**3m**:

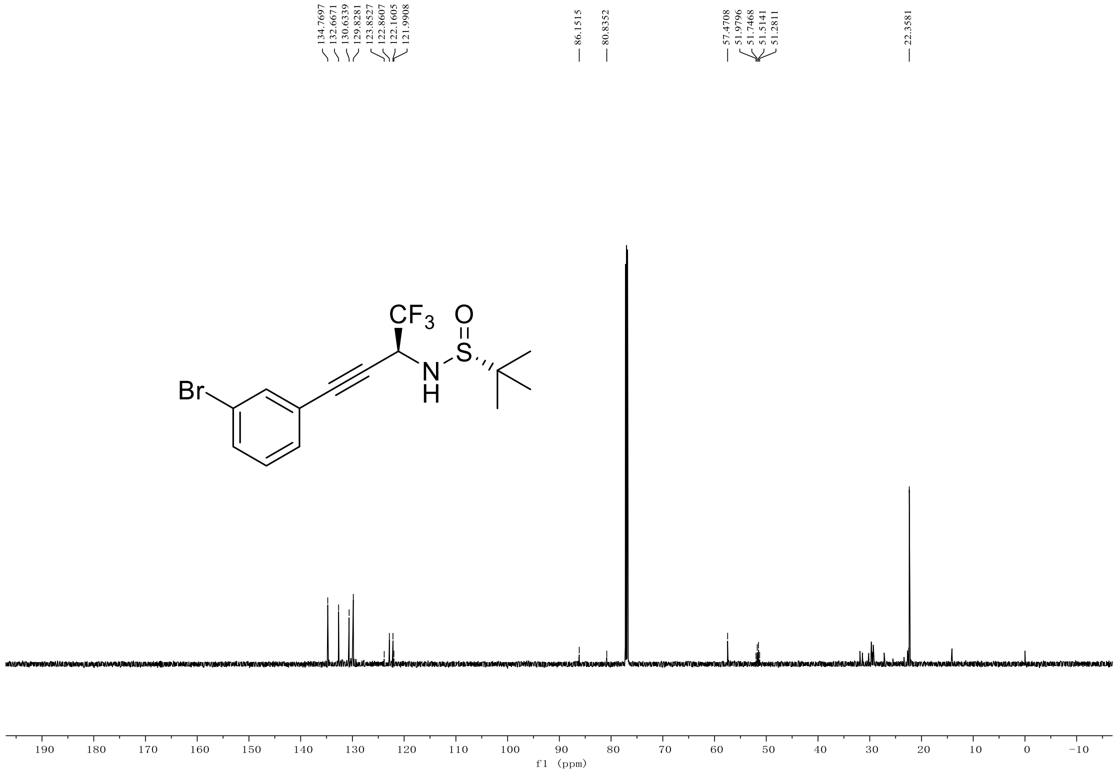

^{19}F NMR (565 MHz, $CDCl_3$) of (*R*s, *S*)-**3m**:

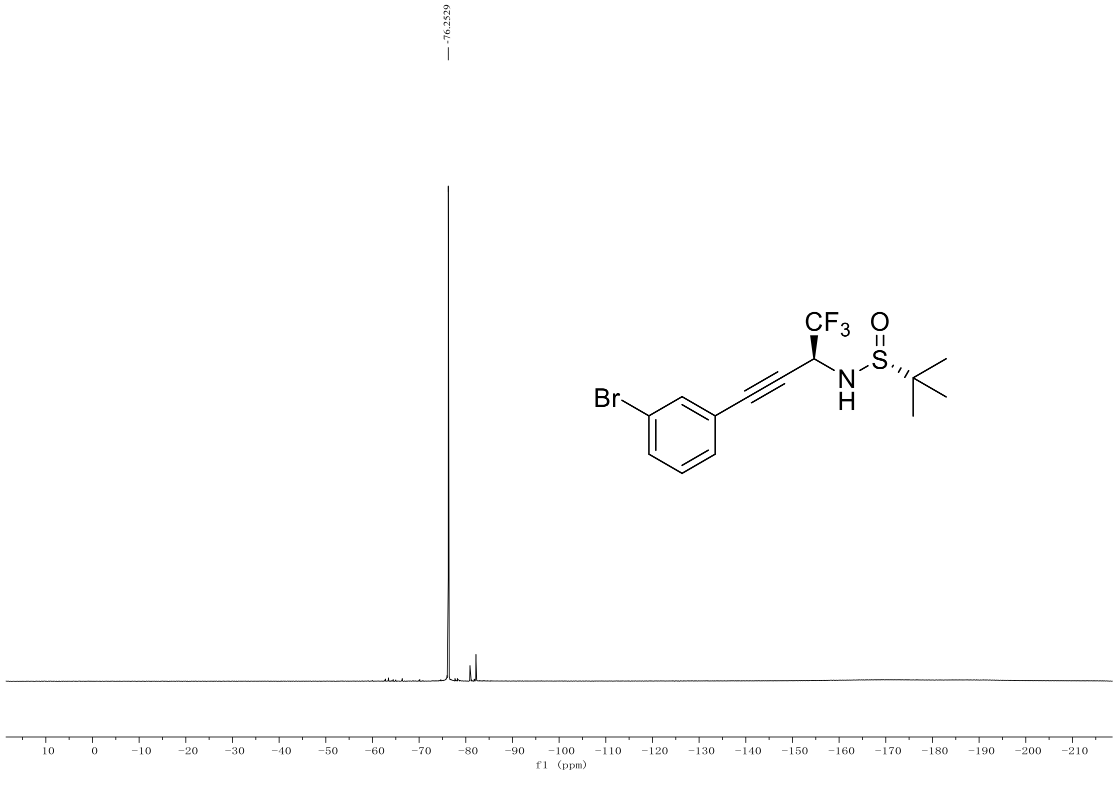

^{1}H NMR (600 MHz, $CDCl_3$) of (*R*s, *R*)-**3n**:

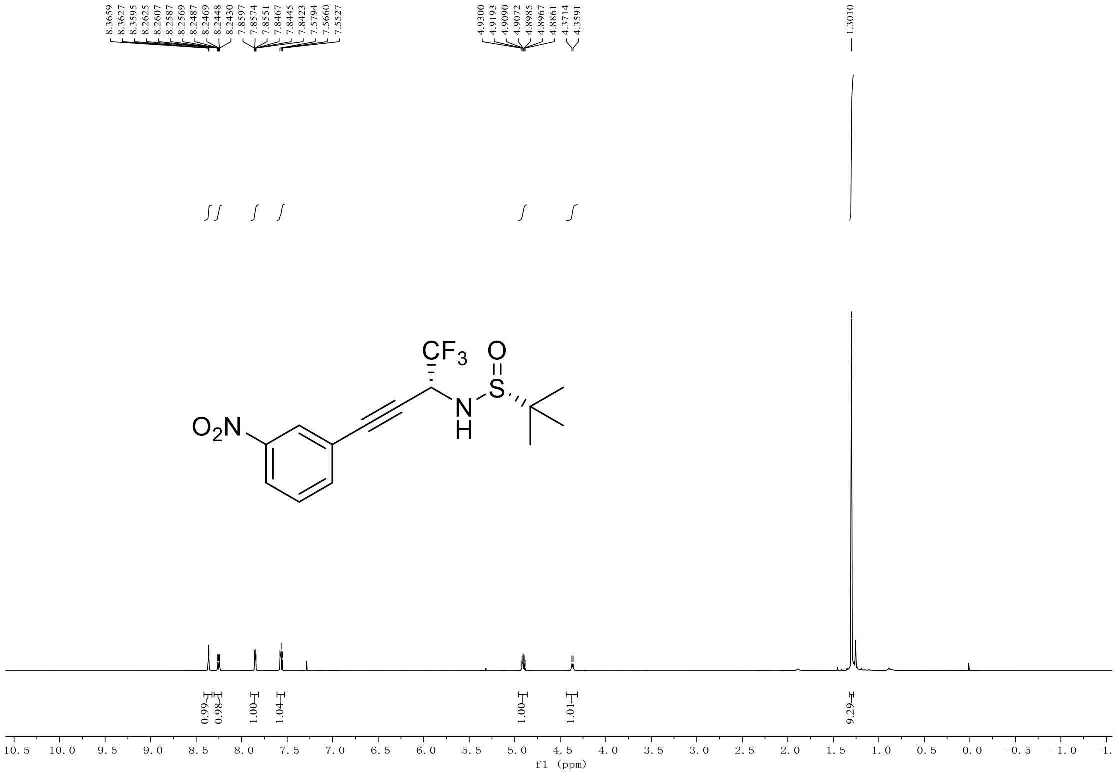

^{13}C NMR (150 MHz, $CDCl_3$) of (*R*s, *R*)-**3n**:

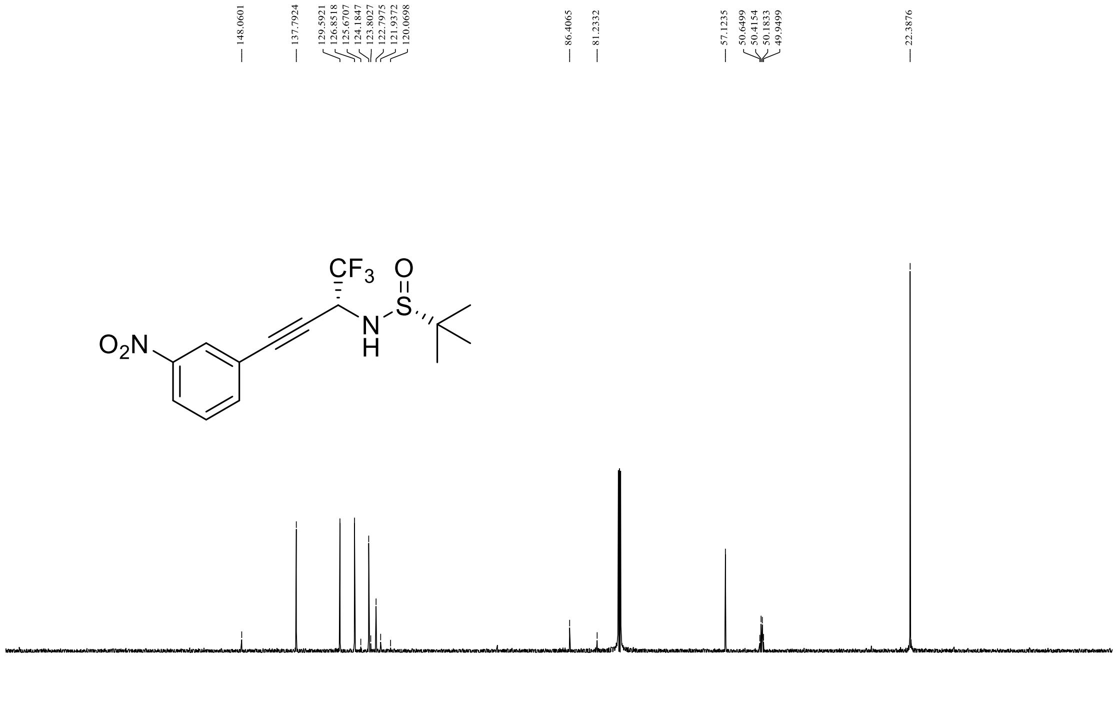

^{19}F NMR (565 MHz, $CDCl_3$) of (R_s, R)-**3n**:

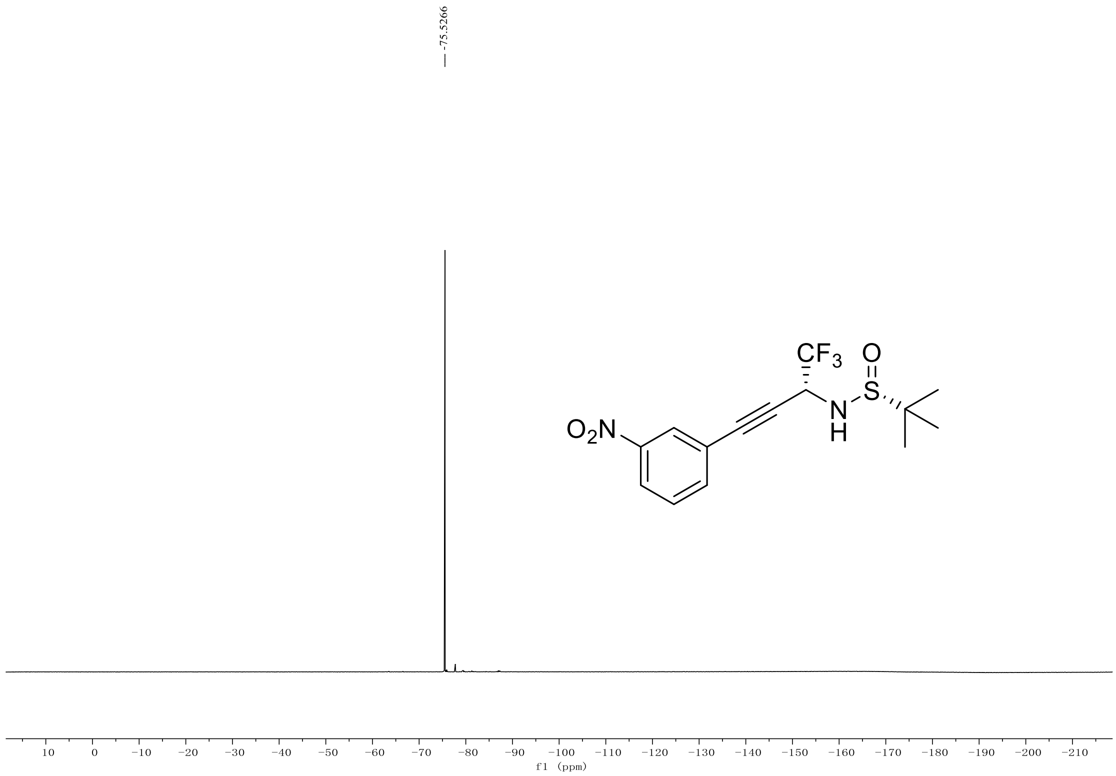

^{1}H NMR (600 MHz, $CDCl_3$) of (R_s, S)-**3n**:

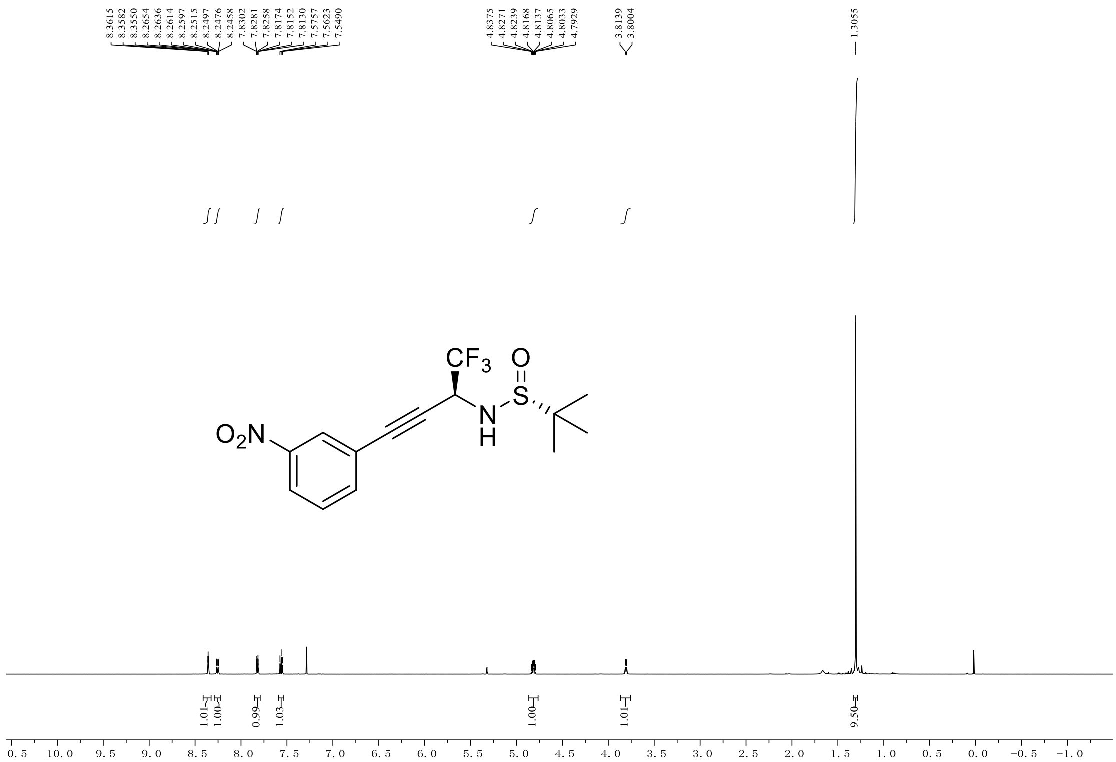

^{13}C NMR (150 MHz, $CDCl_3$) of (R_s, S)-**3n**:

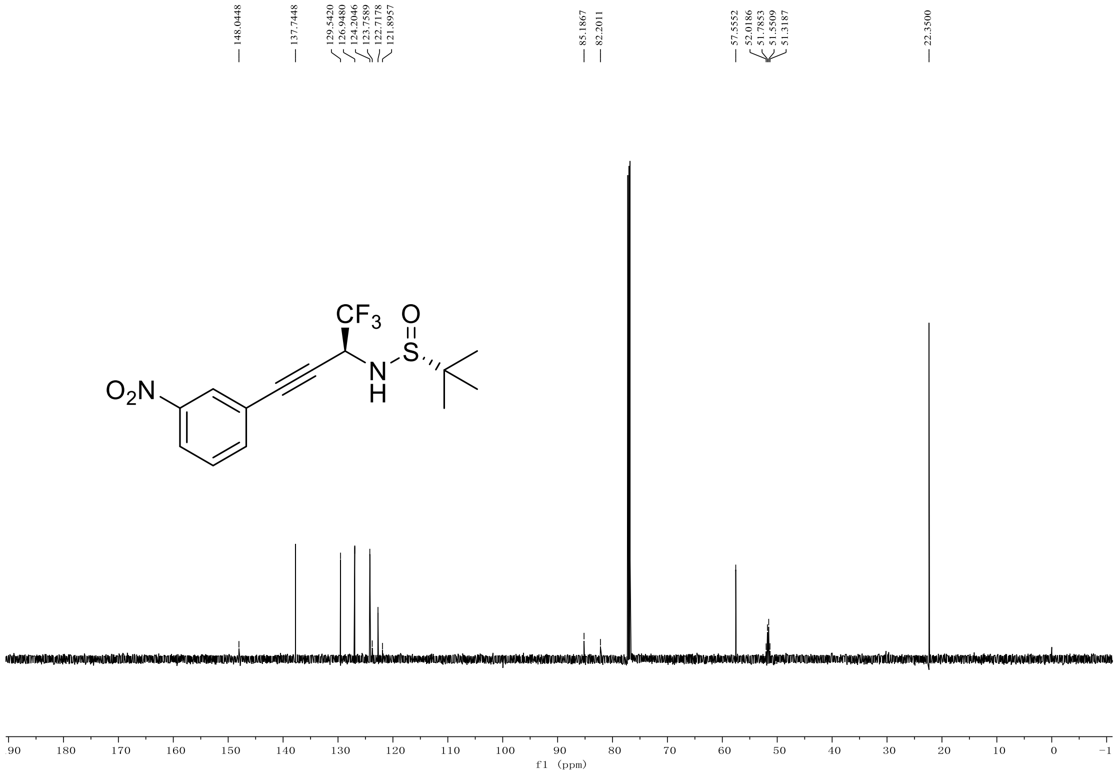

^{19}F NMR (565 MHz, $CDCl_3$) of (R_s, S)-**3n**:

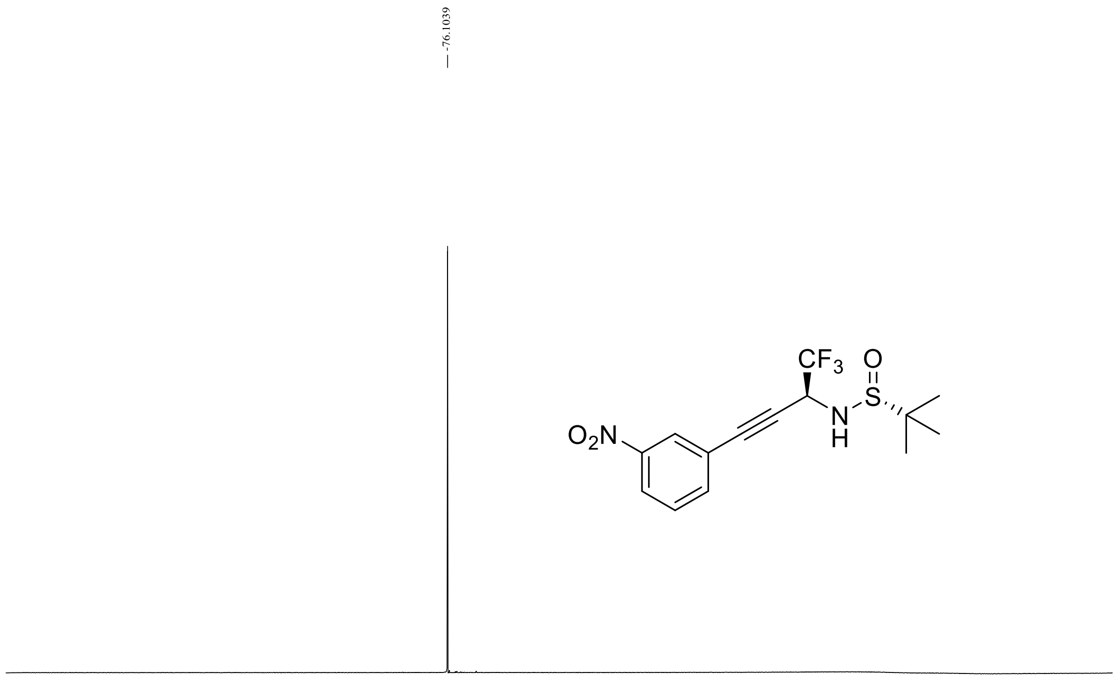

^{1}H NMR (400 MHz, $CDCl_3$) of (*R*)-**4**:

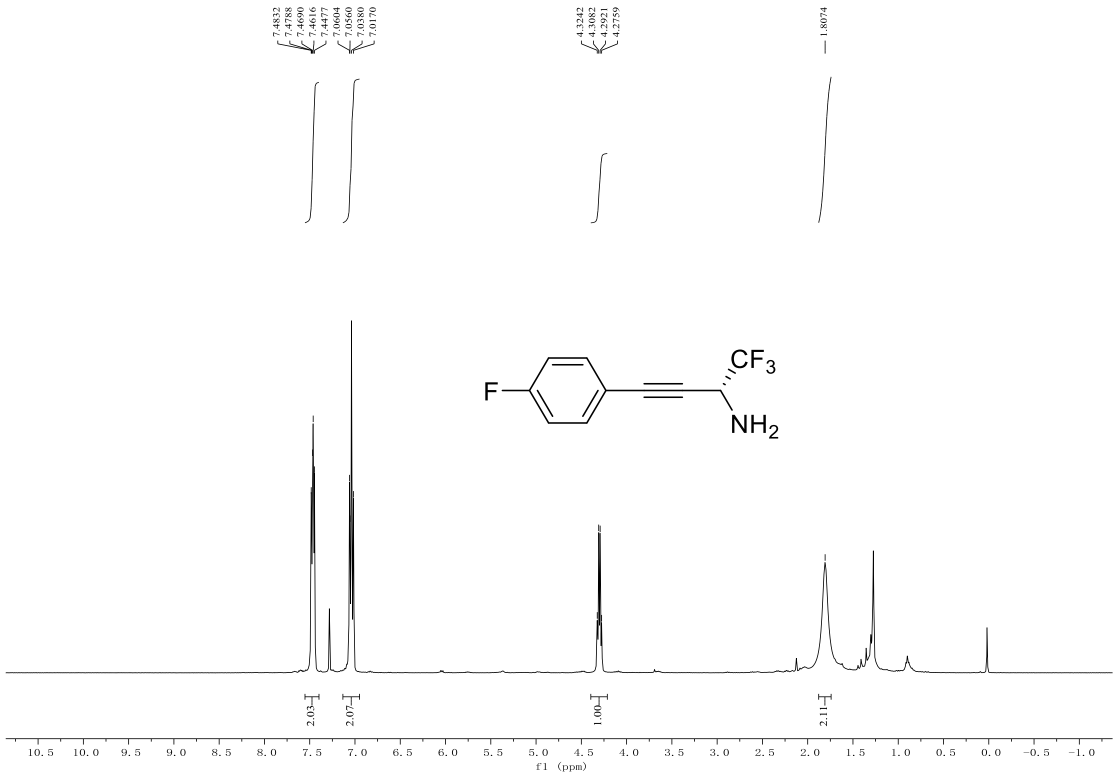

^{13}C NMR (150 MHz, $CDCl_3$) of (*R*)-**4**:

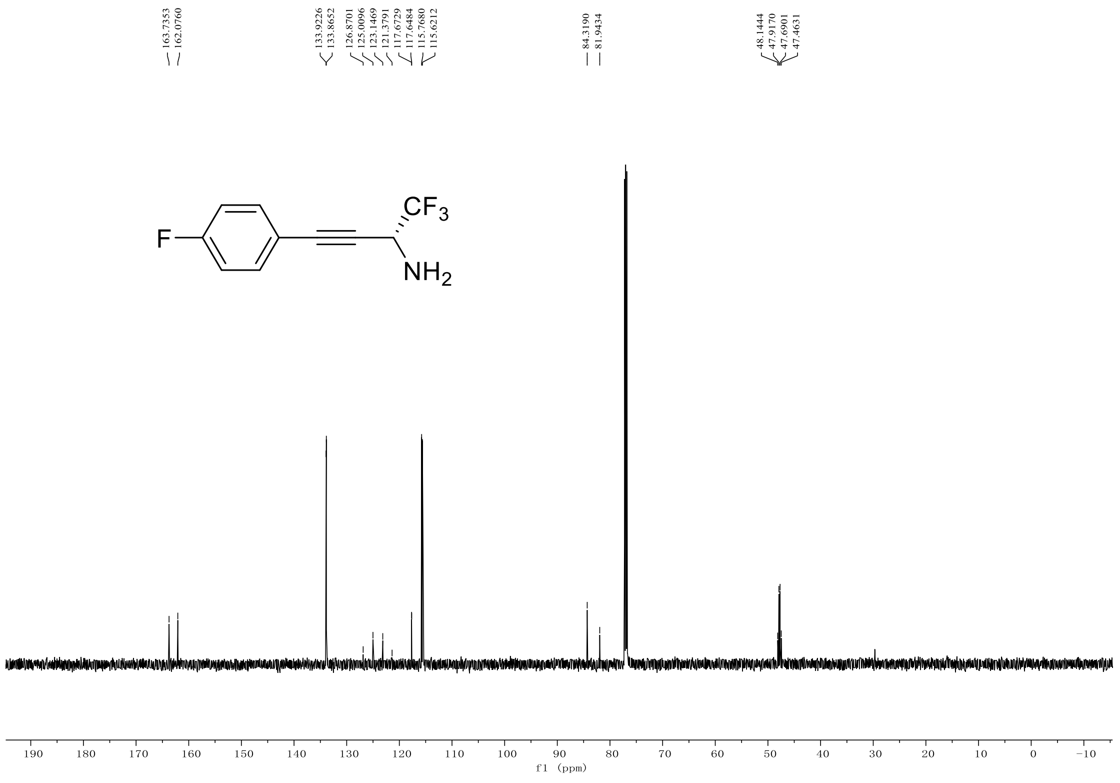

^{19}F NMR (565 MHz, $CDCl_3$) of (*R*)-**4**:

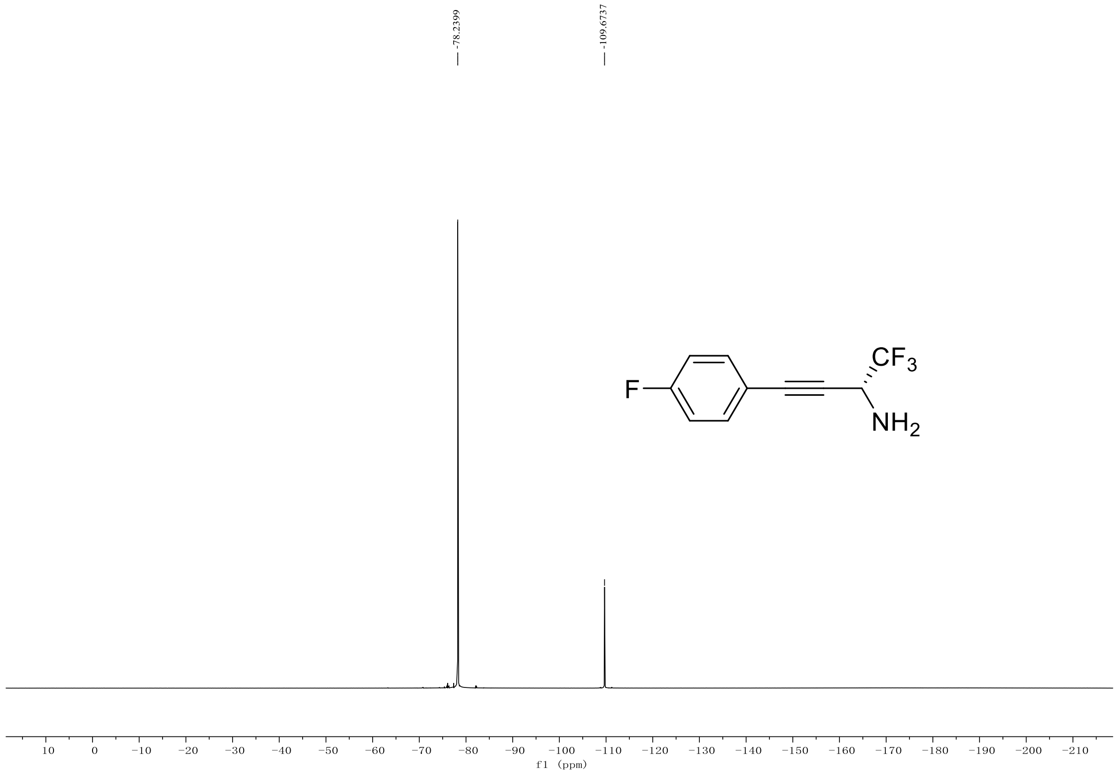

Supplement: File 1 — Experimental details and spectral data. [file Beilstein_J_Org_Chem-16-2671-s001.pdf]
